# Substrate Flexibility of the Flavin‐Dependent Dihydropyrrole Oxidases PigB and HapB Involved in Antibiotic Prodigiosin Biosynthesis

**DOI:** 10.1002/cbic.201900424

**Published:** 2019-10-21

**Authors:** Maxime Couturier, Hiral D. Bhalara, Suresh R. Chawrai, Rita Monson, Neil R. Williamson, George P. C. Salmond, Finian J. Leeper

**Affiliations:** ^1^ Department of Chemistry University of Cambridge Lensfield Road Cambridge CB2 1EW UK; ^2^ Department of Biochemistry University of Cambridge Tennis Court Road Cambridge CB2 1QW UK

**Keywords:** dihydropyrroles, FAD-dependent oxidase, mutasynthesis, prodigiosin, synthesis

## Abstract

In the biosynthesis of the tripyrrolic pigment prodigiosin, PigB is a predicted flavin‐dependent oxidase responsible for the formation of 2‐methyl‐3‐amylpyrrole (MAP) from a dihydropyrrole. To prove which dihydropyrrole is the true intermediate, both possibilities, 5‐methyl‐4‐pentyl‐3,4‐dihydro‐2*H*‐pyrrole (**5 a**, resulting from transamination of the aldehyde of 3‐acetyloctanal) and 2‐methyl‐3‐pentyl‐3,4‐dihydro‐2*H*‐pyrrole (**6**, resulting from transamination of the ketone), were synthesised. Only **5 a** restored pigment production in a strain of *Serratia* sp. ATCC 39006 blocked earlier in MAP biosynthesis. PigB is membrane‐associated and inactive when its transmembrane domain was deleted, but HapB, its homologue in *Hahella chejuensis*, lacks the transmembrane domain and is active in solution. Two colourimetric assays for PigB and HapB were developed, and the HapB‐catalysed reaction was kinetically characterised. Ten analogues of **5 a** were synthesised, varying in the C2 and C3 side chains, and tested as substrates of HapB in vitro and for restoration of pigment production in *Serratia* ΔpigD in vivo. All lengths of side chain tested at C3 were accepted, but only short side chains at C2 were accepted. The knowledge that **5 a** is an intermediate in prodigiosin biosynthesis and the ease of synthesis of analogues of **5 a** makes a range of prodigiosin analogues readily available by mutasynthesis.

## Graphical abstract

**Seeing red**: Prodigiosin has long caught researchers’ eyes due to its bright red colour. One of the biosynthetic enzymes in *Serratia*, PigB, and its homologue in *Hahella*, HapB, are shown to be FAD‐dependent oxidases that oxidise dihydropyrroles to the corresponding pyrrole. Their substrate specificity has been explored. Dihydropyrroles that are substrates turn *Serratia ΔpigD* red due to restoration of prodiginine biosynthesis.

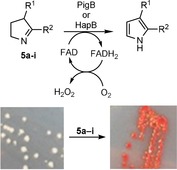

## Introduction

Prodiginines are a group of tripyrrolic secondary metabolites well known for their vivid red colour. In recent years, research on these compounds has intensified as they have displayed various useful biological activities. For instance, the most famous prodiginine, prodigiosin **1**, has shown anticancer,^1^ immunosuppressive,^2^ antimalarial,^3^ antimicrobial^4^ and antifungal^5^ activities. The potency of prodigiosin against Gram positive bacteria such as *Bacillus subtilis* 6 or *Staphylococcus aureus* 4 is also of interest.

**Figure 1:**
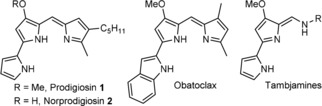
Structures of prodigiosin and related compounds.

Despite this promising activity, no drug derived from prodigiosin has made it to the market. The analogue obatoclax (Figure 1) went to phase II clinical trials against several types of cancer but its development was stopped due to the lack of significant response.^7^, ^8^, ^9^ Thus, discovery and synthesis of new prodiginines with improved biological properties is a potential approach toward new medicines.^10^, ^11^

Total chemical synthesis of prodigiosin and its analogues can be challenging. It is often achieved through the condensation of an analogue of monopyrrole 2‐methyl‐3‐amylpyrrole (MAP) **3** and the bipyrrole 4‐methoxy‐2,2′‐bipyrrole‐5‐carbaldehyde (MBC) **4**. MAP can be obtained in two steps from octan‐2‐one^12^ but, even though improvements have been made,^13^, ^14^ formation of MBC and its analogues still requires complex multistep synthesis. A better understanding of the biosynthetic process leading to the formation of prodigiosin could help to address this challenge.

**Scheme 1:**
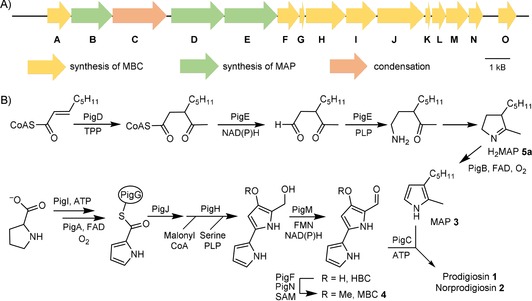
A) Prodigiosin biosynthetic cluster in *S*. 39006. B) Biosynthetic pathway to prodigiosin.

Prodigiosin is the product of a bifurcated pathway (Scheme 1 B) with the synthesis of MAP on one side and the synthesis of MBC on the other, with a final enzyme, PigC, catalysing the condensation of the two to form prodigiosin.^15^, ^16^, ^17^, ^18^ Even though the organisms producing prodigiosin are quite diverse,^18^, ^19^, ^20^ the genetic clusters coding for prodigiosin biosynthesis have shown a large degree of conservation. MBC is also an intermediate in the biosynthesis of the other prodiginines^21^ and the tambjamines^22^ (Figure 1). It is formed by a combination of PKS and NRPS enzymes and a number of studies have characterized the different enzymes involved.^23^ Studies of PigC have shown its flexibility toward analogues of both MAP^24^, ^25^, ^26^ and MBC.^27^

Unlike MBC, the pathway for formation of MAP is specific to prodigiosin;^18^ formation of the monopyrrole units in other prodiginines, such as undecylprodigiosin, follows an entirely different pathway.^21^ The first enzyme in the pathway, PigD, has been shown to be a thiamine diphosphate‐dependent enzyme adding an acetyl group from pyruvate onto the β‐position of an oct‐2‐enoyl thioester.^28^ The other two enzymes involved in MAP biosynthesis have not been characterized and little is known about their substrate specificity or kinetics. However, gene‐knockout experiments showed that PigB is responsible for the final step of the MAP pathway, which was deduced to be oxidation of a dihydroMAP (H_2_MAP) to MAP.^18^

Here, we report studies of PigB and HapB, its homologue from *Hahella chejuensis*. These include elucidation of the natural substrate, in vitro assays on HapB with H_2_MAP analogues, and in vivo substrate flexibility experiments on PigB in *Serratia* sp. ATCC 39006 (*S*. 39006) to characterize these oxidases.

## Results and Discussion

### Elucidation of the natural substrate

**Scheme 2:**
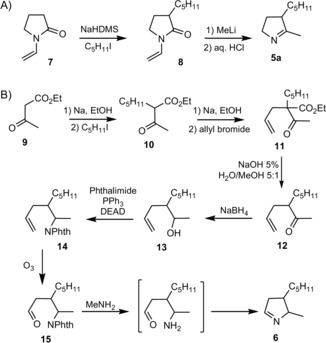
Synthesis of two potential substrates for PigB.

Using LC–MS analysis, Williamson et al.^18^ showed that the substrate of PigB was the result of the transamination of 3‐acetyloctanal followed by an intramolecular cyclisation. However, 3‐acetyloctanal contains two carbonyl groups and the transamination might happen at either of them, resulting in two possible isomers of H_2_MAP **5 a** and **6** (Scheme 2). In order to establish which isomer is the true biosynthetic intermediate, we synthesized both isomers.

Dihydropyrrole **5 a** was obtained in just two synthetic steps: the enolate of *N*‐vinylpyrrolidinone **7** was first reacted with 1‐iodopentane to give intermediate **8**, which was then reacted with methyl lithium to give, after deprotection, racemic **5 a** (Scheme 2 A).

The synthesis of the other potential substrate **6** was rather longer. The enolate of ethyl acetoacetate **9** was first reacted with 1‐iodopentane to give **10**. Then the enolate of **10** was reacted with allyl bromide, leading to **11**. Hydrolysis and decarboxylation gave **12** and reduction of the ketone gave alcohol **13**. In a Gabriel synthesis of primary amines, **13** was then reacted with phthalimide leading to **14** and ozonolysis afforded **15**. The amino‐ketone obtained after deprotection cyclised spontaneously to form dihydropyrrole **6** (Scheme 2 B) as a mixture of diastereomers.

**Figure 2:**
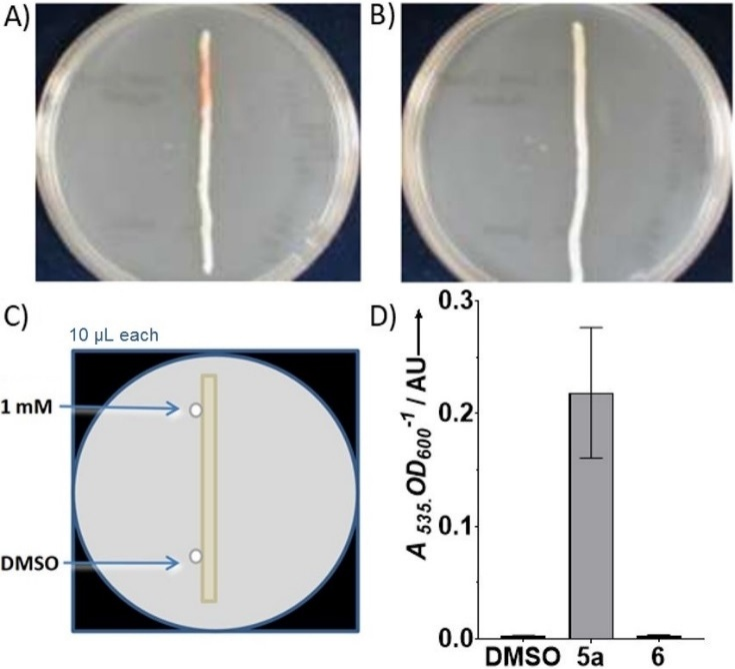
Results of feeding of the two potential isomers of PigB substrate A) *S39006 ΔpigD* fed with **5**. B) *S39006 ΔpigD* fed with **6**. C) Principle of the feeding assay on agar plate. D) Results of feedings in liquid culture (error bars: SD; *n*=3).

To test whether **5 a** and/or **6** can be precursors of prodigiosin, they were spotted (in DMSO) next to streaks on agar of *Serratia NW13* 18 (here called *S*. 39006 *ΔpigD*), a strain with an in‐frame deletion in *pigD*, which therefore presents a white phenotype. As illustrated in Figure 2 A–B, pigmentation was restored with **5 a** but not with **6**. In addition, cultures of *S*. 39006 *ΔpigD* in liquid media were supplemented with 100 μm of the two potential substrates and incubated for 24 h before extracting the pigment into acidified ethanol for quantification (Figure 2 D). Again, **5 a** led to a restoration of pigmentation whereas **6** did not. Thus, only **5 a** can be accepted as a substrate by PigB and converted into MAP. No pigment was observed when *S*. 39006 *ΔpigB* was treated with **5 a** (see Figure S9 in the Supporting Information), showing that the presence of PigB is necessary for pigmentation, and so **5 a** does not spontaneously oxidize in air to give MAP.

Finally, both H_2_MAP accumulated by a *ΔpigB* strain (*Serratia* NW14 in ref. 13) and synthetic **5 a** showed a peak of the correct mass at the same retention time (1.7 min) by LC–MS (Supporting Information). We can conclude that the structure of the biosynthetic intermediate, dihydroMAP (H_2_MAP), is **5 a** and not **6**. This means that the PigE‐catalysed transamination of 3‐acetyloctanal is on the aldehyde and not the ketone (Scheme 1 B).

### Synthesis of dihydroMAP analogues

**Scheme 3:**
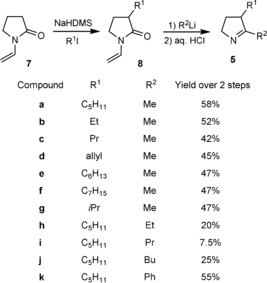
H_2_MAP analogues synthesised.

Ten analogues **5 b**–**k** of H_2_MAP **5 a** with various substituents on the C2 and C3 positions were obtained via the same steps as described above for the synthesis of **5 a** (Scheme 3). The enolate of *N*‐vinyl‐pyrrolidinone **7** was first alkylated with various alkyl halides to give the intermediates **8 a**–**g**, which were then reacted with various alkyllithium compounds to give, after deprotection, H_2_MAP analogues **5 b**–**k** in overall yields between 7.5 and 60 %.

### Characterisation of HapB

BLAST analysis^29^ of HapB and PigB indicated homology with flavin‐dependent amine oxidases. PHYRE^30^ identified them as most similar to protoporphyrinogen IX oxidases, which are *trans*‐membrane proteins, involved in the biosynthesis of haem, that catalyse the six‐electron oxidation of protoporphyrinogen IX to protoporphyrin IX.^31^ The homology model generated by PHYRE is shown in Figure S2.

Direct comparison using Artemis ACT^32^ and EBI′s Clustal Omega^33^ between HapB and PigB indicated that the two proteins share 41 % identity. In particular, amino acids 41–54 of HapB (residues 155–167 in PigB) were highly conserved and were predicted to be part of the flavin binding site. Two other highly conserved regions are residues 255–296 and 517–533 in HapB (368–409 and 630–646 in PigB), which could indicate that those regions form part of the active site (see Figure S1).

A major difference between PigB and HapB is that PigB is 125 residues longer and the first 113 residues of PigB have no equivalents in HapB, but a protein homologous to this N‐terminal region of PigB is encoded by a separate gene *HCH_*06023 in the *H. chejuensis* cluster. It is predicted to be a transmembrane domain by TMHMM (v2.0)^34^ and PredictProtein programs.^35^

**Figure 3:**
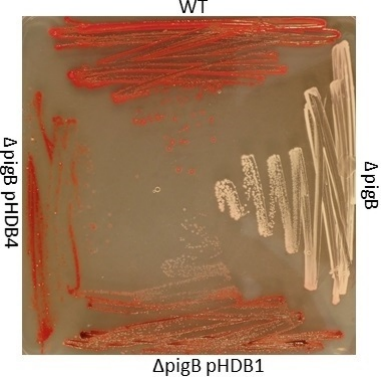
Mutants of *S39006* grown on PGM agar plates with 0.1 mm IPTG Top: wild‐type; right: *ΔpigB* mutant; bottom: *ΔpigB* (pHDB1); right; *ΔpigB* (pHDB4).

The gene *hapB* was amplified by PCR from *H. chejuensis* genomic DNA and cloned into the pQE80‐L vector, giving plasmid pHDB1, encoding HapB with an N terminal His_6_‐tag. After transformation of *S*. 39006 *ΔpigB* with pHDB1, pigmentation was restored by complementation (Figure 3).

Similarly, two plasmids expressing PigB were constructed using *S*. 39006 gDNA and a pQE80::*oriT* vector, leading to the plasmids pHDB3 and pHDB4 which code for PigB with His_6_ tags at the N and C termini, respectively. Transformation of *S*. 39006 *ΔpigB* with pHDB3 or pHDB4 again restored pigmentation in both cases (shown for pHDB4 in Figure 3). A truncated version of *pigB*, without the sequence that encodes for the transmembrane domain of the protein was also cloned into a pQE80‐L vector, forming plasmid pHDB2. However, when this vector was used to transform *S*. 39006 *ΔpigB*, no pigmentation was recovered, which suggests that the truncated protein is inactive.

**Figure 4:**
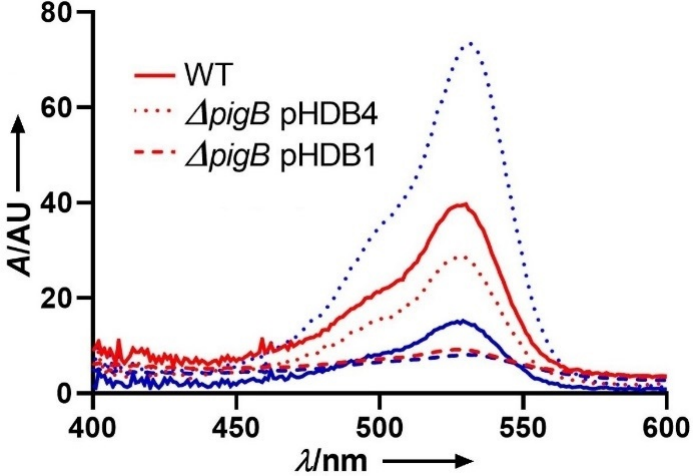
UV spectra of prodigiosin extracts from *S39006 ΔpigB* complemented either with PigB or HapB; red: no IPTG, blue [IPTG]=1 mm.

The pigments extracted^36^ from *S*. 39006 wild‐type, *ΔpigB* (pHDB1), and *ΔpigB* (pHDB4) all gave the same UV/Vis spectra (Figure 4). With induction by IPTG, the quantity of pigment produced by *ΔpigB* (pHDB4) was approximately twice that of the WT. The pigmentation obtained with *S*. 39006 *ΔpigB* (pHDB1) was lower than with the other strains, even with addition of IPTG. This unexpected result might be due to poor solubility of HapB or to the absence of the transmembrane domain mentioned above.

To prove that the pigment observed was indeed prodigiosin, the extracts obtained without IPTG induction were analysed by high resolution MS. This showed a mixture of prodigiosin **1** and norprodigiosin **2** (Figure 1) in all three cases (Figure S5). Norprodigiosin results from the same biosynthetic pathway but instead of MBC **4**, PigC catalyses the condensation of MAP with HBC, the previous intermediate lacking the O‐methyl group (Scheme 1). Thus, even without induction by IPTG, the plasmids did restore production of MAP, proving that HapB, like PigB, does indeed catalyse the oxidation of H_2_MAP to MAP.

Expression of PigB in *Escherichia coli* BL21 led to very low levels of the desired protein but active PigB could be prepared by transforming pHDB4 into *E. coli* C43, a mutant of BL21 which is less sensitive to toxicity.^37^ After lysis, ultracentrifugation was used to obtain the membrane‐fraction containing PigB. Using the Ehrlich's assay described below, this fraction was found to be active, but protein purity was only around 50 %. As purification of membrane‐bound proteins can be problematic, we chose to focus our efforts on characterisation of the soluble HapB. Plasmid pHDB1 was used to overexpress HapB in *E. coli* BL21 cells, and the protein was purified by Ni‐NTA affinity chromatography.

As explained above, the sequence of HapB is consistent with a flavin‐dependent oxidase. The purified protein is yellow in colour and its UV/Vis spectrum is consistent with the presence of a flavin (*λ*_max_=378 and 450 nm). The nature of the cofactor was elucidated by comparing the cofactor released upon denaturation of active protein with standards of FAD and FMN on reversed‐phase HPLC.^38^ FMN and FAD gave peaks at 5.3 and 4.3 min, respectively. Denatured HapB gave a single peak eluting at 4.4 min, indicating that the cofactor of HapB was FAD. Repeated purification of HapB produced a colourless solution of protein, lacking the flavin absorbance peaks, showing that the flavin was noncovalently bound to the protein and could be lost. Finally, site directed mutagenesis using overlap extension PCR^39^ of Asp69 (predicted in the homology model to form hydrogen bonds with the ribose hydroxy groups of FAD, Figure S2) to alanine resulted in a colourless inactive protein. This indicates that the FAD cofactor is essential and does not bind to the mutated protein.

**Scheme 4:**
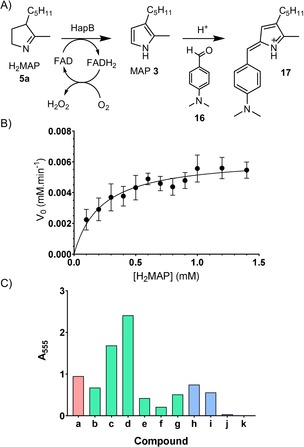
A) Principle of the Ehrlich's assay. B) Kinetic characterization of HapB (error bars show the standard deviation of three separate measurements). C) Flexibility of HapB for substrates **5 a**–**k** as determined by Ehrlich's assay and normalised relative to the *A*_555_ value obtained with **5 a**: **5 a** (red) natural substrate H_2_MAP; **5 b**–**g** (green) analogues with modifications at C3, **5 h**–**k** (blue) analogues with modifications at C2.

As MAP is an α‐unsubstituted pyrrole, the reaction of HapB can be assayed colourimetrically by using Ehrlich's reagent (Scheme 4 A). HapB and H_2_MAP were incubated together at 30 °C and at different time points aliquots were treated with trifluoroacetic acid and HgCl_2_ followed by *p*‐dimethylaminobenzaldehyde **16** (Ehrlich's reagent). MAP reacts with **16** (20 min, room temperature) to form the condensation product **17**, which has a *λ*_max_ at 555 nm. When this assay was performed using HapB (0.457 μm) and H_2_MAP (0.1–1.4 mm), the absorbance increased linearly with time. Plotting the rate of reaction against concentration of H_2_MAP gave a Michaelis–Menten curve (Scheme 4 B) for which the best‐fit parameters were *V*_max_ 6.3±0.6 μm min^−1^ and *K*_M_ 0.22±0.08 mm. This gives a turnover number *k*_cat_ of 13.7±1.2 min^−1^.

The substrate specificity of HapB was studied by incubating it with various analogues of H_2_MAP (0.5 mm, 5 min, 30 °C) and then performing the above Ehrlich's assay (Scheme 4 C). It appeared that all substitutions in the C3 position (compounds **5 b**–**f**) could be accepted as substrates. Interestingly, the most positive results were obtained with the linear C_3_ chains (**5 c** and **d**), but further decrease in size to an ethyl group **5 b** led to a sudden decrease in activity. Similarly, elongation of the chain (**5 e** and **f**) led to a diminution of the activity by more than a factor of 2. Modification on the C2 substituent showed that elongations of up two carbons (**5 h**,**i**) could be accommodated, but longer chains (**5 j**,**k**) were hardly accepted at all.

**Scheme 5:**
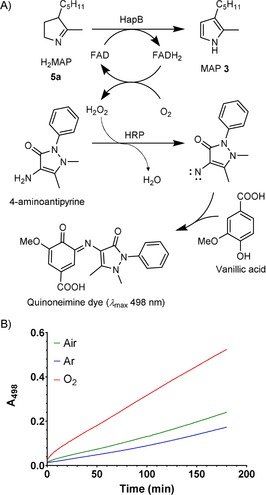
A) Principle of HRP‐coupled assay. B) Monitoring of the H_2_O_2_ production during H_2_MAP oxidation after bubbling the reaction mixture with different gases, red: O_2_, green: air, blue: argon.

Flavin dependent amine oxidases generally consume oxygen and release H_2_O_2_. To check that H_2_O_2_ is produced, a coupled assay was performed, in which the H_2_O_2_ released oxidises 4‐aminoantipyrine, catalysed by horseradish peroxidase (HRP), and the oxidised 4‐aminoantipyrine reacts with vanillic acid to produce a red quinoneimine dye absorbing at 498 nm^40^ (Scheme 5 A). On performing this assay with **5 a** and HapB, the absorbance at 498 nm showed a continuous increase, proving that H_2_O_2_ is a by‐product of the reaction. This HRP assay provides an alternative to the Ehrlich's assay for measurement of HapB activity. The HRP assay was then used to show that the reaction is oxygen‐dependent. The assay solution was bubbled with either oxygen or argon or air for 10 min before addition of HapB. In all three cases, the absorbance at 498 nm increased linearly with time, with the highest rate for the O_2_‐bubbled assay, then air and finally argon (Scheme 5 B). These results indicate that O_2_ is necessary to reoxidise FADH_2_ to FAD, and that this reoxidation is at least partially rate‐limiting.

### Mutasynthesis of prodigiosin analogues

**Figure 5:**
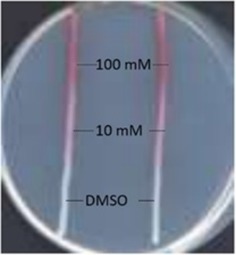
Example of the agar plate test for prodiginine production. 5 μL of H_2_MAP solution at 100 mm. 10 or 0 mm in DMSO was spotted next to the streaks of *S*. 39006 *ΔpigD*.

Similar to the feeding experiments performed with **5 a** and **6** (Figure 2), initial tests on the production of prodigiosin analogues from H_2_MAP analogues **5 b**–**k** employed growth on a solid medium. Two streaks of *S*. 39006 *ΔpigD* were grown on agar plates.^41^ In three wells next to the streaks, 5 μL of DMSO (as a negative control), and H_2_MAP **5 a** (as the positive control) or analogues **5 b**–**k** at 10 mm or 100 mm in DMSO were spotted (Figure 5). The ability of PigB and PigC to use these substrates could then be detected by the red colouration that appeared in the adjacent streak. Modification of H_2_MAP in the C3 position (**5 b**–**g**) led to pigmentation in all six cases (see the Supporting Information). This was consistent with the in vitro assays of HapB described above and additionally shows that the MAP analogue resulting from the oxidation of **5 b**–**g** by PigB was accepted as a substrate for PigC. Both increasing (**5 e**,**f**) and decreasing the size of the chain (**5 b**–**d**) led to a result very similar to the natural substrate.

In contrast to the flexibility toward different substituents at C3, it seemed that hardly any substitution in the C2 position could be accommodated. Increasing the chain‐length to an ethyl group (compound **5 h**) still led to pigmentation but only at 100 mm. With longer chains attached to C2 (compounds **5 i**–**k**) the cells remained white.

**Figure 6:**
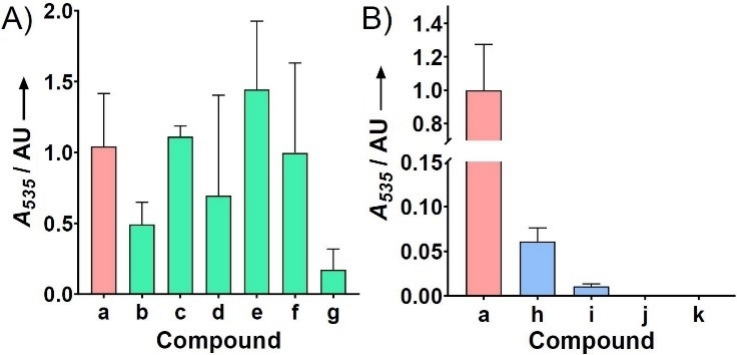
Pigment production of *S39006 ΔpigD* fed with analogues of H_2_MAP **5 a**–**k** normalised relative to the *A*_535_ value obtained with **5 a**: A) Modification on the C3 position. B) Modification on the C2 position (error bars: SD; *n*=3).

To quantify the pigment production, liquid cultures of *S*. 39006 *ΔpigD* were fed with H_2_MAP analogues **5 a**–**k** (10 μm, 30 °C, 16 h), then centrifuged, and the pellets extracted with 4 % HCl (1 m) in EtOH. UV/Vis spectra of the extracts were recorded.^41^ Due to the presence of other coloured metabolites (e.g., carotenoids^42^), a sloping background absorbance in the region 450–570 nm was always observed even in the case of the negative control. To assess the part of the absorbance due to prodigiosin, a baseline value was subtracted from the absorbance at 535 nm. The results, normalised to the positive control (H_2_MAP **5 a**), are shown in Figure 6.

As with the qualitative assay on agar plates, all the substitutions in the C3 position led to pigmentation. Compound **5 g** led to a low level of pigment, however. This might be due to the branched side chain at C3 being a poor substrate for PigC. No major difference could be observed between the natural substrate **5 a** and compounds **5 e** and **5 f**, which present a longer chain on the C3 position. Many of the unnatural prodiginines produced here have previously been produced by feeding MAP analogues to strains blocked in MAP biosynthesis^24^, ^25^, ^26^ but the prodiginine with the branched isopropyl chain starting with **5 g** has not been described before.

Unsurprisingly, compounds **5 j** and **5 k** did not allow the recovery of any pigment. As shown above HapB cannot accommodate the larger side chains at C2 and it is probable that PigB has the same limitation. However, compounds **5 h** and **5 i** did allow recovery of a very small amounts of pigment.

It appears that the compounds that showed positive results with the in vitro assay with HapB also led to a restoration of pigmentation in the feeding experiments. This indicates that the substrate flexibility of HapB and PigB for these substrates is similar. Also, it seems that, for the production of prodigiosin analogues from H_2_MAP analogues, the substrate‐specificity of PigB is the more important factor and the substrate‐specificity of PigC is either similar to that of PigB, or more relaxed.

## Conclusion

This work has shown that the dihydropyrrole intermediate in the biosynthesis of prodigiosin **1** is **5 a**, the product of transamination of the aldehyde group of 3‐acetyloctanal. This dihydropyrrole **5 a** is a substrate for PigB, which oxidises it to the pyrrole MAP **3**. We have also characterized in vitro for the first time HapB, the homologue of PigB from *H. chejuensis*, and determined the kinetic parameters of this FAD‐dependent oxidase. Finally, we combined in vitro and in vivo assays, to confirm that HapB and PigB have similar substrate selectivities and produce a variety of analogues of prodigiosin. In particular, we report here the first example of a prodiginine with a branched side chain obtained by mutasynthesis.

## Experimental Section

**Prodiginine production and extraction**: *Strains producing pigment* [*S*. 39006 *WT, ΔpigB* (pHDB4) and *ΔpigB* (pHDB1)]. In triplicate, a solution of Luria‐Bertani broth (LB: tryptone 10 g L^−1^, yeast extract 5 g L^−1^, NaCl 10 g L^−1^) supplemented with 0.25 m sorbitol was inoculated with an overnight culture of the strain. The culture was incubated at 30 °C, 250 rpm for 16 h. Cells (25 mL) were then pelleted (2219 *g*, 20 min, 4 °C) and the supernatant was discarded. The pellets were vortexed for 1 min in 5 mL of acidified EtOH (4 % 1 m HCl). After centrifugation the supernatant gave the desired prodiginine extract.

*Strain not producing prodigiosin*: In triplicate *S*. 39006 *ΔpigD*. was grown in LB supplemented with 0.25 m sorbitol at 30 °C, 250 rpm until OD_600_ reached 4. H_2_MAP or one of its analogues (10 μm final concentration) was then added and the cells were grown for a further 16 h. Cells were then pelleted and prodiginine extracted as described above.

*Prodigiosin quantification*: A spectrum of the extract described above was recorded on a Cary 300‐Bio UV/Vis spectrophotometer. If the A_535_>2, the sample was diluted ten times in EtOH (4 % HCl), and the recorded absorbance was subsequently multiplied by 10. Prodigiosin maximum absorbance is at 535 nm, but to correct for the absorption of other extracted compounds, *A*_535_ was corrected using the formula (1)

(1) Acorr535=A535-(85×A570+35×A450)/120

as pure prodigiosin has negligible absorbance at 450 and 570 nm, whereas extracts from strains not producing prodigiosin showed a fairly linear decrease in absorbance across this region, with the result that Acorr535 for a nonproducing strain was close to zero. Using a value for the extinction coefficient of prodigiosin of *ϵ*_535_=139 800 m ^−1^ cm^−1^,^12^ the average quantities of prodiginine produced from a 25 mL culture starting, from the H_2_MAP analogue shown, were **5 b**, 11 nmol; **5 c**, 55 nmol; **5 d**, 34 nmol; **5 e**, 72 nmol; **5 f**, 22 nmol; **5 g**, 1.2 nmol; **5 h**, 21 nmol; **5 i**, 4 nmol: **5 j** and **5 k**, 0 nmol. These experiments were run on four different occasions and on each occasion cultures fed with H_2_MAP **5 a** were included as the positive control. The amounts of prodigiosin **1** produced varied (from 7 to 347 nmol) and the production of prodigiosin analogues shown in Figure 6 is relative to the prodigiosin produced in the parallel cultures fed with **5 a**.

*Compound feeding on agar plates*: 1 L of medium contained peptone (5 g), glycerol (10 mL) and agar (15 g). The strain was streaked along a straight line on the agar and incubated at 30 °C for 48 h. A solution (5 μL) of the compound in DMSO was then spotted next to the streak and the plate incubated at 30 °C overnight. In some cases a shorter time was sufficient to see pigmentation.

**Ehrlich's assay**: Solution A (MES pH 6.0 10 mm, HapB 29 μg mL^−1^) and B (MES pH 6.0 10 mm, H_2_MAP 0.1 to 1.4 mm, DMSO 8 %) were prepared and incubated separately for 10 and 5 min, respectively. The reaction was then initiated by mixing 25 μL of each per well in a 96‐well plate. The reaction was stopped by addition of 50 μL of stop reagent (HgCl_2_ 100 mm, TCA 10 % in H_2_O) followed by 100 μL of Ehrlich's reagent (2 % *p*‐dimethyl‐aminobenzaldehyde in acetic acid with 16 % perchloric acid). The plate was incubated for a further 20 min at room temperature and an absorbance spectrum was recorded. The baseline was subtracted using the formula (2)

(2) Acorr535=A555-(A460+A650)/2

as the pigment **17** derived by reaction of synthetic MAP with Ehrlich's reagent has negligible absorbance at 460 and 650 nm, while negative controls (with no added MAP) showed a fairly linear decrease in absorbance across this region, with the result that Acorr535 for a negative control was close to zero.

**Horseradish peroxidase (HRP) assay**: To 0.2 mL of chromogenic solution (4‐aminoantipyrine 0.5 mm, vanillic acid 1 mm, HEPES pH 7.4 100 mm, HRP 4 U mL^−1^) was added H_2_MAP (2 mm final conc.) and H_2_O was added to a final volume of 0.8 mL. The mixture was incubated 10 min at 30 °C. During that incubation period, gases (O_2_, air or argon) could be bubbled through the solution. HapB (10 μg) was then added and the mixture incubated at 30 °C while recording the absorbance at 498 nm.

## Conflict of interest

*The authors declare no conflict of interest*.

## Supporting information

As a service to our authors and readers, this journal provides supporting information supplied by the authors. Such materials are peer reviewed and may be re‐organized for online delivery, but are not copy‐edited or typeset. Technical support issues arising from supporting information (other than missing files) should be addressed to the authors.

Supplementary
