# Supplementary material for: Substrate Flexibility of the Flavin‐Dependent Dihydropyrrole Oxidases PigB and HapB Involved in Antibiotic Prodigiosin Biosynthesis
Source: Chembiochem. 2019 Oct 21;21(4):523–30. doi: 10.1002/cbic.201900424 (PMC7065143; doi:10.1002/cbic.201900424)
Supplement: Supplementary file 1 — Supplementary [file CBIC-21-523-s001.pdf]

CHEMBIOCHEM

Supporting Information

Substrate Flexibility of the Flavin-Dependent Dihydropyrrole Oxidases PigB and HapB Involved in Antibiotic Prodigiosin Biosynthesis

Maxime Couturier,[a] Hiral D. Bhalara,[a] Suresh R. Chawrai,[a] Rita Monson,[b] Neil R. Williamson,[b] George P. C. Salmond,[b] and Finian J. Leeper*[a]

cbic_201900424_sm_miscellaneous_information.pdf

SUPPORTING INFORMATION

General considerations

Standard solution-phase chemistry was used for the synthesis of the various biosynthetic intermediates and their analogues. All reactions were carried out under N_2 in pre-dried glassware and all organic solvents used were freshly distilled. Solvents and reagents for anhydrous reactions were dried by conventional methods prior to use. Milli-Q deionised water was used in all chemical reactions and biochemical work. High temperature reactions were carried out using a silicone oil bath. Yields refer to chromatographically and spectroscopically pure compounds. Microanalyses were performed by the University of Cambridge Microanalytical Laboratory in the Department of Chemistry, and are quoted to the nearest 0.1% for all elements except for hydrogen, which is quoted to the nearest 0.05%. Reported atomic percentages are within the error limits of ± 0.3%.

Nuclear Magnetic Resonance spectroscopy

NMR Spectra were recorded in deuterated solvents (as specified) using a Bruker AM/DPX-400 (^{1}H NMR at 400 MHz, ^{13}C NMR at 100 MHz). Chemical Shifts (δ) are quoted in parts per million (ppm) and referenced to solvent peaks. Coupling constants (J) are reported in Hz. The multiplicities and general assignments of the spectroscopic data are denoted as: singlet (s), doublet (d), triplet (t), quartet (q), quintet (quin), double doublet (dd), double double doublet (ddd), double doublet triplet (ddt), double triplet (dt), triple triplet (tt), double quartet (dq), unresolved multiplet (m), and broad (br).

LC-MS

All LC-MS analyses were performed with an Alliance HT Waters 2795 Separations Module on a Waters Atlantis dC18 4.6 x 30 mm, 3 µm column eluted with a gradient of 10 mM aqueous ammonium acetate containing 0.1% formic acid to 95% acetonitrile over 8 min, coupled to a Waters Micromass ZQ Quadrupole Mass Analyser (using electrospray ionisation (ESI)) with accuracy no greater than 0.4 Da.

Mass Spectrometry

Electron impact (EI) ionization mass spectra were recorded using a Kratos Concept spectrometer and electrospray ionization (ESI) spectra on a Micromass Q-Tof spectrometer. Accurate masses were obtained with a Waters LCT Premier high resolution mass spectrometer and/or a Bruker Daltonics FTLCR BioApex II 4.7e using ESI.

Infra-Red (IR) Spectroscopy

IR spectra were recorded neat on a diamond/ZeSe plate using a Perkin-Elmer Spectrum One FT-IR Universal Attenuated Transmittance Reflectance (ATR) sampling accessory spectrometer with internal referencing. Characteristic absorption maxima (λ_{max}) are reported in wavenumbers (cm^{-1}) and the following abbreviations are used: w, weak; m, medium; s, strong; br, broad.

Silica Gel Chromatography

Flash Column chromatography was performed using 230-400 mesh Kieselgel 60 silica. Analytical thin layer chromatography (TLC) was performed on commercial silica gel plates (Merck glass- or aluminium-backed plates coated with a 0.20 mm layer of silica gel 60 with fluorescent indicator UV254). These plates were visualised using either ultraviolet light (254 or 365 nm), or by staining the plates with potassium permanganate or vanillin solutions or with Ehrlich's reagent.

Ultraviolet-Visible (UV-Vis) spectrophotometry

All the UV-Vis spectra for the various compounds were taken on a Varian Cary 100 Bio Spectrophotometer with a 6 x 6 peltier multicell holder using 1 cm path length using either 1 ml quartz cuvettes or polystyrene disposable cuvettes.

Bioinformatics of PigB and HapB

```
PigB      MIIQRLFGILYMLAGLAKAFPQFENVPAVLRQAAIANQGTWYAAASIWLGAHGDVINILV      60
HapB      ------------------------------------------------------------      0

PigB      GVVLFGSGVILMLNPLWTTLVIYAQLLMMAVFVVILHQSQPQVMLLDGVFALAALYMLRG      120
HapB      -------------------------------------------------------MESQD      5

PigB      QYHRKPKPRTFPTTSFSLPTPSSES--SFSAPLGDEYDVVIIGGGASGLTAASEFTHERV      178
HapB      NSQYAGEAPVFPSKDFECNQGSFDDTDTSPPQHAGDYDVIVVGGGISGLSSAWKLRERKL      65

PigB      LVLEKSSTFGGNARYHTFNRLKHPTAGVCFQEPFPGSNMLRLLKKIGLEGKYKSNEKDTL      238
HapB      LVLDQRDRFGGAARLEQRDGLLYASGASCFQLPTGHNEVSHLLQDLDLWNQWRSTAEDTL      125

PigB      VFFDTFLLLKCLGEIVVGFIKQPRYLLKLSVWGLTSQLFLHAIIGKPYVVAAKQLGDPIF      298
HapB      VIFDTKRLMKGLGEVTAALLKQPKELLKPAVWGLTANLLYSAMSGKPFISAEKKLGDPMF      185

PigB      ADLYTFLDKFSPRGDFYPRLPWTPNGSWSKAHMELLDNISLYTYLFEPDKLGRLPEQLRP      358
HapB      ADLFQYLNRFTPDSGKHPAMPWREGCDWTREEMELFDSVSLHDLLFDPATRRSLPQDLIP      245

PigB      PARLGKLVENAVSTTLRVECLDIHDVSAYVGLHFLVGYLRGNLVTLPGGNGSISAGLCKY      418
HapB      RHRFGSLVKDAVETTLRVECLAIKDVSAYVGLHFLVGYLYRPLVTFPGGNGYIADRIRQR      305

PigB      LSHQRNVTLQNHVQLTAVEPQHNGTCIQFTINGQPRQVQAQQIIWAAPKTQLATWLPGLP      478
HapB      LMSTGSCKFKAGSRAYSITQNQEGVKVCFQQEGKNYYANANALIWAGAKHAAVSVVDGLP      365

PigB      AKQLAAIKNIRHEDYYLANVFLSKPVLGHSFGGYMIEPDS-NKDPFSWCKAGTCLVANWM      537
HapB      QQQKAAIAEIEHRDYAIAGVYLKKAALANYFGGYVIEGDIGGRYPNSWCRSGVCLAANWK      425

PigB      DDHADVDVGVLTLLKPTTRSERQDRTAQNAFLALQQQTYAEIAKVLRNIGIGAEVIEDIQ      597
HapB      DPSYAGGLGVLTLLKPISGAADQGKLGKADFRNLQQTAYGEVRDMLVATGHSPDLIEDIK      485

PigB      IWYWPAGLVTSVVGQQAEGVFETARQSFENIHFANQDSVGVGNIESAILSGIDAANAVKA      657
HapB      LWRWPHGLVVSKVGQMKHDVFVNASQPVGAVFFANQDSVGMGNMESAIWAGCHAAEQVRR      545

PigB      QLMDTENVVEVAG-------     670
HapB      HFRSHSSVVSHIGDYAATQS     565
```

Figure S1. Alignment of PigB and HapB by EBI Clustal Omega. In orange is the conserved region predicted to form part of the FAD binding site. In red are two other highly conserved regions, Asp69 of HapB is in bold

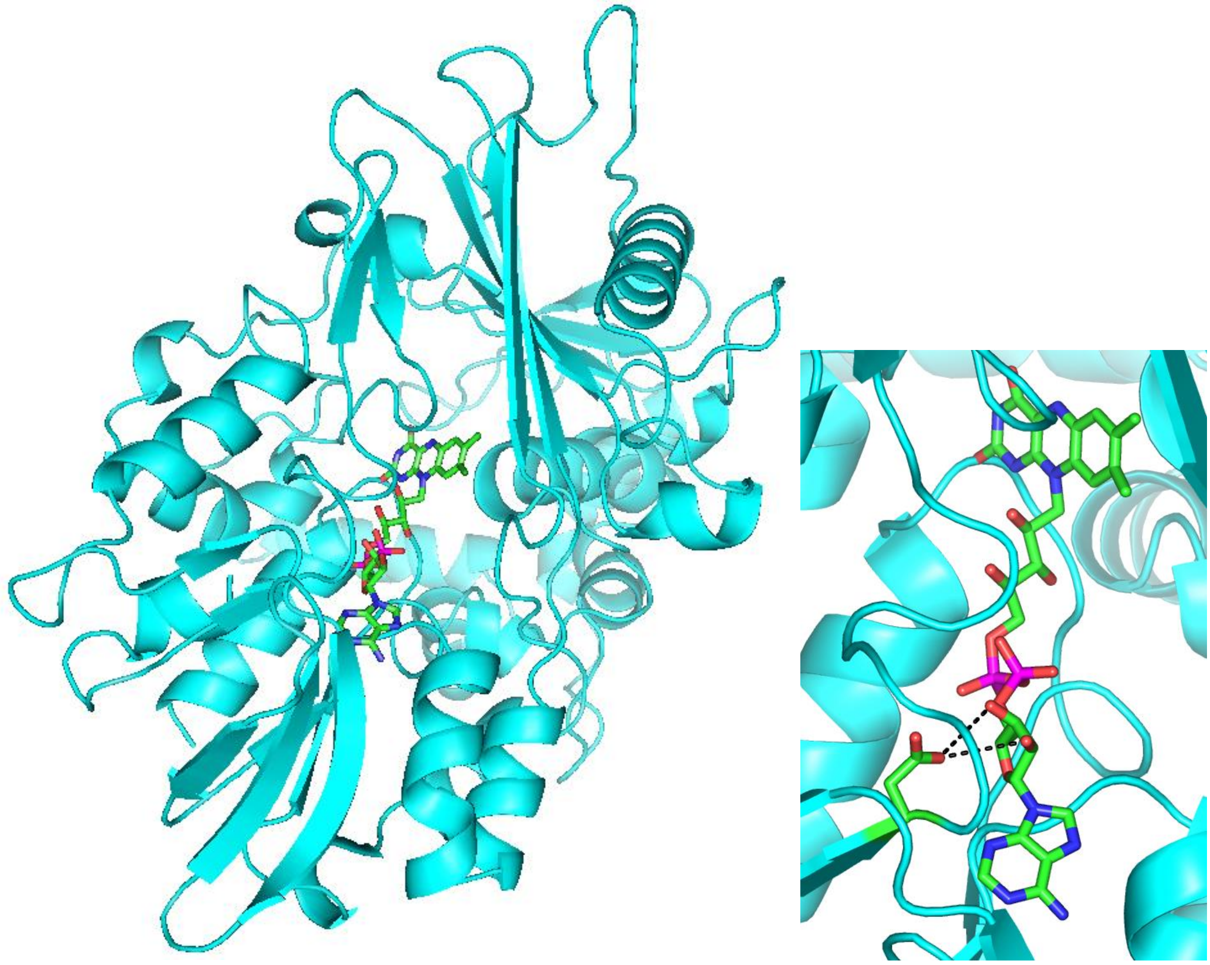

Figure S2. Left: homology model for HapB generated by the PHYRE website from the crystal structure of protoporphyrinogen oxidase from *Myxococcus xanthus* (PDB entry 2ive). The pose of the FAD is from overlay of residues 55-542 of the homology model (which lacks the FAD) with the 2ive crystal structure (which contains FAD). Right: detail of the binding site for FAD in the model, showing the hydrogen bonds from Asp69 to the 2'- and 3'-OH groups of the adenosine part of FAD. Images generated by PyMol.

PCR

Name	Sequence 5'-3'	Restriction enzyme	Usage
HB1	GCG**GGATCC**GAATCACAAGACAACTCACAG	*Bam*HI	*hapB* amplification upstream
HB2	GCG**GCATGC**CTAGCTCTGGGTCGCTGCGTAG	*Sph*I	*hapB* amplification downstream
HB3	CCC**GGATCC**CCTAAGCCGAGAACCTTCCCGA C	*Bam*HI	Truncated *pigB* amplification upstream
HB4	GCG**CTGCAG**TCATCCCGCCACCTCCACGAC	*Pst*I	*pigB* amplification (N-terminal His_6 tag) downstream
HB5	CCC**GGATCC**ATTATTCAACGGCTCTTCGGCATC	*Bam*HI	*pigB* amplification (N-terminal His_6 tag) upstream
HB6	GGG**GAATTC**CGGTTTAAGGGGAACATCATC	*Eco*RI	*pigB* amplification (C-terminal His_6 tag) downstream
HB7	CCC**CTGCAG**TTAGTGATGGTGATGGTGATGTCCCGCCACCTCCACGAC	*Pst*I	*pigB* amplification (C-terminal His_6 tag) upstream
HB8	CGCCGAAGCGATCCCGTTGAGCCAGCACCAATAGCTTGCGCTC		*hapB* site directed mutagenesis upstream
HB9	TCAACGGGATCGCTTCGGCG		*hapB* site directed mutagenesis downstream

Table S1: PCR oligonucleotides (restriction site in bold)

Compound	Quantity (µl)
gDNA template	1
Oligonucleotide 1 (10 µM)	2.5
Oligonucleotide 2 (10 µM)	2.5
dNTP (10 µM)	0.5
5x HF Buffer	5
Phusion® Hi-Fidelity DNA polymerase	0.2
SD-H_2O	Up to 50 µl

Table S1: Composition of PCR mixture

PCR cycle

PCR cycling parameters included initial denaturation (5 min, 95 °C), then 30 cycles of denaturation (30 s, 95 °C), annealing (30 s, 55 °C) and extension (2 min, 72 °C), and a final extension (10 min, 72 °C). The amplified PCR products were analysed by agarose gel electrophoresis. 0.8% (w/v) agarose gel containing ethidium bromide was run at 85 V in 1×TAE buffer.

Plasmid construction (pHDB1-4)

PCR amplification was conducted in the conditions described above. *Hahella chejuensis* gDNA was amplified using oligonucleotide pair HB1 & HB2 (pHB1). Three separate PCRs were carried out to amplify *S.* 39006 gDNA using oligonucleotide pair (HB3 & HB4, pHDB2), (HB4 & HB5, pHDB3) and (HB6 & HB7, pHDB4). After purification of the PCR product of the correct size by agarose gel electrophoresis, it was digested with *Bam*HI/*Sph*I (pHDB1), *Bam*HI/*Pst*I (pHDB2-3) or *Eco*RI/*Pst*I (pHDB4) restriction enzymes for 2 h at 37 °C and ligated together with a compatibly digested pQE80L (pHDB1-2) or pQE80::*oriT* (pHDB3-4). The generated plasmids were transformed into *E. coli* DH5α and sequenced to confirm the absence of mutation.

Site-directed mutagenesis by overlap extension PCR

Asp69 of HapB was replaced by alanine by site-directed mutagenesis. PCR was performed using a DNA Engine® Peltier Thermal Cycler using PCR cycling parameters similar to those above. Two separate PCRs were carried out using oligonucleotide pairs (HB1 & HB7) and (HB2 & HB8). These PCR products were purified by agarose gel electrophoresis. A second PCR was then carried out using these two PCR products as primers. After purification of the PCR product of the correct size by agarose gel electrophoresis, it was digested with *Bam*HI/*Sph*I restriction enzymes for 2 h at 37 °C and ligated together with a compatibly digested pQE80-L vector to generated plasmid pHDB5. This was subsequently transformed into *E. coli* DH5α. pHDB5 was sequenced by the DNA Sequencing Facility, Department of Biochemistry, University of Cambridge, to confirm the desired point mutation. The plasmid was transformed into protein expression strain *E. coli* BL21 (DE3) and the mutant HapB protein was overexpressed and purified by Ni-NTA affinity chromatography.

**Strain**	Genotype/phenotype	Source or reference
Serratia		
ATCC 39006	Wild type (Car+, Pig+)	Bycroft *et al*.[1]
NW14 (noted *ΔpigB*)	In-frame *pigBΔ* (3270–4773, 501 aa Δ)	Williamson *et al*.[2]
NW13 (noted *ΔpigD*)	In-frame *pigDΔ* (7895–9446, 517 aa Δ)	Williamson *et al*.
E. coli		
DH5α	*supE*44, *hsdR17*(r_K^- m_K^-), *thi-1, recA1, gyrA96* (Nal^R), *relA1, Δlac* (*lacIZYA-argF*) *U169 deoR* (ϕ80 *lacZ*ΔM15)	Grant *et al.*[3]
BL21 (DE3)	*B* F^- *ompT gal dcm lon* $hsdS_B(r_B^- m_B^-)$ *λ(DE3 [lacI lacUV5-T7p07 ind1 sam7 nin5]) [malB⁺]* $_{K\text{-}12}$ (λ^S)	Studier *et al*.[4]
C43	F^- *ompT gal dcm* $hsdS_B(r_B^- m_B^-)$*(DE3)*	NEB
**Plasmid**		
pQE80-L	6xHis fusion expression vector, Amp^R	Qiagen
pQE80::*oriT*	6xHis fusion expression vector containing *oriT* gene for bacterial conjugation, Amp^R	Monson *et al.*[5]
pHDB1	1698 bp *Bam*HI/*Sph*I containing N-His_6-*hapB* ligated into pQE80L	This study
pHDB2	1639 bp *Bam*HI/*Pst*I containing N-His_6-*pigB* ligated into pQE80L (truncated)	This study
pHDB3	2013 bp *Bam*HI/*Pst*I containing N-His_6-*pigB* ligated into pQE80::*oriT*	This study
pHDB4	2013 bp *Eco*RI/*Pst*I containing C-His_6-pig*B* ligated into pQE80::*oriT*	This study

Table S2: List of bacterial strains and plasmids

[1] B. W. Bycroft, C. Maslen, S. J. Box, A. G. Brown, J. W. Tyler, *J. Chem. Soc. Chem. Commun.* **1987**, *0*, 1623.

[2] N. R. Williamson, H. T. Simonsen, R. A. A. Ahmed, G. Goldet, H. Slater, L. Woodley, F. J. Leeper, G. P. C. Salmond, *Mol. Microbiol.* **2005**, *56*, 971–989.

[3] S. G. Grant, J. Jessee, F. R. Bloom, D. Hanahan, *Proc. Natl. Acad. Sci.* **1990**, *87*, 4645–4649.

[4] F. W. Studier, B. A. Moffatt, *J. Mol. Biol.* **1986**, *189*, 113–30.

[5] R. Monson, D. S. Smith, M. A. Matilla, K. Roberts, E. Richardson, A. Drew, N. Williamson, J. Ramsay, M. Welch, G. P. C. Salmond, *Front. Microbiol.* **2015**, *6*, 1442.

Protein purification

Lysis buffer (pH 8.0)	50 mM NaH_2PO_4
	300 mM NaCl
	20 mM imidazole
Wash buffer (pH 8.0)	50 mM NaH_2PO_4
	300 mM NaCl
	20 mM imidazole
	20% glycerol (v/v)
Elution buffer (pH 8.0)	50 mM NaH_2PO_4
	300 mM NaCl
	250 mM imidazole
Storage buffer (pH 8.0)	20 mM Tris-HCl (pH 7.0)
	250 mM NaCl
	1 mM EDTA
	1 mM DTT

Table S3: Protein purification buffers

Preparation of cell lysates: Cells transformed with suitable vector-constructs were grown at 37 °C on a shaker at 250 rpm to obtain an OD_{600} of 0.6. Cultures were then induced with 1 mM isopropyl-β-D-thiogalactopyranoside (IPTG) at 16 °C for 14-16 h. Aliquots were collected before and after the induction by IPTG to monitor the expression of protein and analyzed by Sodium Dodecyl Sulphate-Polyacrylamide Gel Electrophoresis (SDS-PAGE). Cells were harvested by centrifuging at 5000 rpm for 20 min at 4 °C. The cell pellet was resuspended in the lysis buffer including complete-mini-EDTA-free protease inhibitor cocktail (1 tablet per 10 ml lysis buffer) from Roche.

For protein purification from soluble fraction: The above lysis buffer was supplemented with 1% Triton-X100. The ice-water jacketed suspension was sonicated and then centrifuged at 10,000 rpm at 4 °C for 30 min. The clarified lysate was loaded onto a Ni- NTA (Ni^{2+}-nitrilotriacetate) column (Qiagen) for affinity purification. The column was washed twice with wash buffer and the bound protein was eluted (1 ml fractions) with elution buffer and the fractions analysed by SDS-PAGE. The fractions containing purified protein were pooled and dialyzed in storage buffer.

For protein isolation as membrane fraction: Cell pellets resuspended in lysis buffer without detergents were subjected to lysis using the EmulsiFlex (Avestin) at 4 °C. The cell lysate was then ultra-centrifuged at 28,000 rpm (Beckman Coulter - Optima™ L-100XP, rotor SW28) at 4 °C for 2 h to separate into three layers - the cell debris, membrane fraction and clarified lysate. The membrane fraction after dilution with 50% glycerol was used for SDS-PAGE analysis, activity determination and kinetic studies.

Characterization of HapB

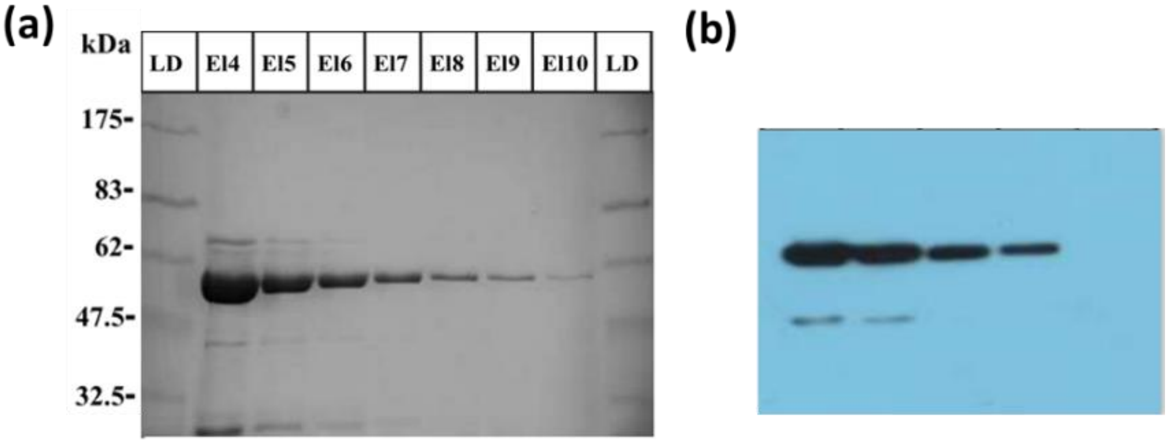

Figure S3: Analysis of HapB by **(a)** SDS-PAGE electrophoresis **(b)** Western blot of a serial dilution of HapB (anti His_6 antibody), LD: ladder, E14-E110: elution fractions

Determination of HapB cofactor

HPLC characterization of HapB cofactor

Chromatography was performed on a Varian ProStar HP-LC system fitted with a Varian ProStar model 410 autosampler and a Varian ProStar Model 320 UV/Vis detector, observing at 254 nm using reversed phase column (Whatman Partisil PXS 10/25 ODS-2) with octadecylsilane chemically bonded to porous silica run at 1 ml/min flow-rate. Eluents: A, 10 mM NH_4OAc in water (pH 7.0) + 0.1% formic acid; B, 95% CH_3CN/H_2O + 0.1% formic acid. Gradient: 0-5 min, 85% A, 15% B; 5-25 min, 85-25% A, 15-75% B; 25-30 min, 25-0% A, 75-100% B; 30-40 min, 0% A, 100% B; 40-45 min, 85% A, 15% B.

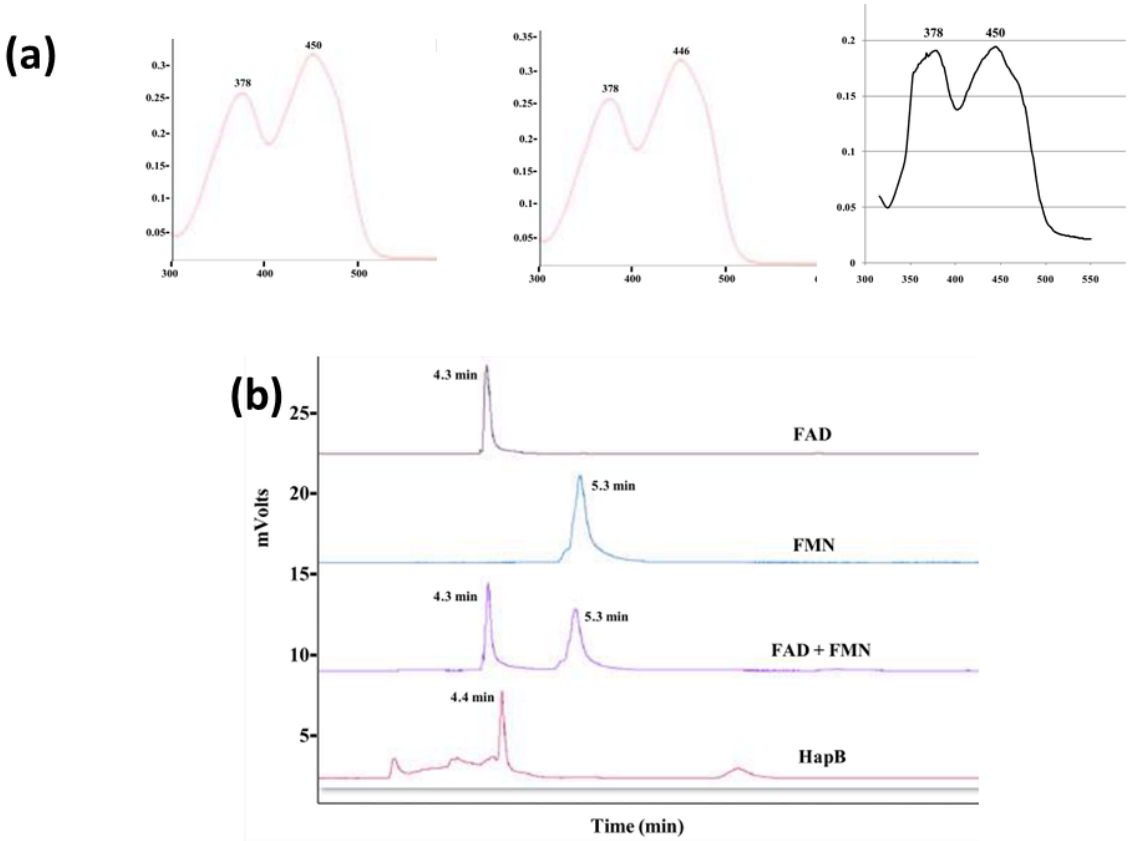

Figure S4: **(a)** Spectra of flavonoids: from left to right FAD, FMN, HapB; **(b)** HPLC analysis of HapB cofactor

Mass spectrometry

Sample preparation for MS

Prodigiosin: 20 ml of LB broth + sorbitol 0.25 M were inoculated with 5 ml of an overnight culture of *Serratia* WT, *ΔpigB* (pHDB4) or *ΔpigB* (pHDB1). In the case of *ΔpigB* (pHDB1) and *ΔpigB* (pHDB4), Ampicillin (0.1 mg.ml^{-1}) was added.

After growing the cell overnight at 30 °C at 250 rpm, pigment was extracted following the method described in the paper. The solvent was then removed under reduced pressure in a pre-weighed flask. The extracts were redissolved in CH_3CN/H_2O 1:1 at 10 mg/ml. When some solid was still visible after vortexing, the sample was filtered through cotton wool.

H_2MAP: 200 ml of LB broth + sorbitol 0.25 M in a 2 L flask were inoculated with a 5 ml overnight culture of *Serratia ΔpigB* (or *ΔpigD* for negative control). The cells were cultured at 30 °C for 16 h. The cultures were centrifuged, and the cell pellets extracted with 100 ml of acetone/Et_2O 4:1. The extracts were concentrated to 5 ml under reduced pressure. 300 µl were taken and diluted to 1 ml in CH_3CN/H_2O 1:1

MS method

H_2MAP samples were run on a Water Xevo G2-S . Eluent A: H_2O+ 0.1% formic acid, eluent B: 95% ACN/ H_2O+0.1 % formic acid. Gradient: 0-0.38 min, 95% A, 5% B; 0.38-3 min, 95-5% A, 5-95% B; 3-4 min, 5% A, 95% B. The chromatograms at 154.16 (H_2MAP+H^+) are then extracted.

Prodigiosin samples were run on a Water LCT Premier. Eluent A H_2O+0.05 % formic acid, eluent B CH_3CN. Flow: 50% A, 50% B for 3 min.

Mass Spectra

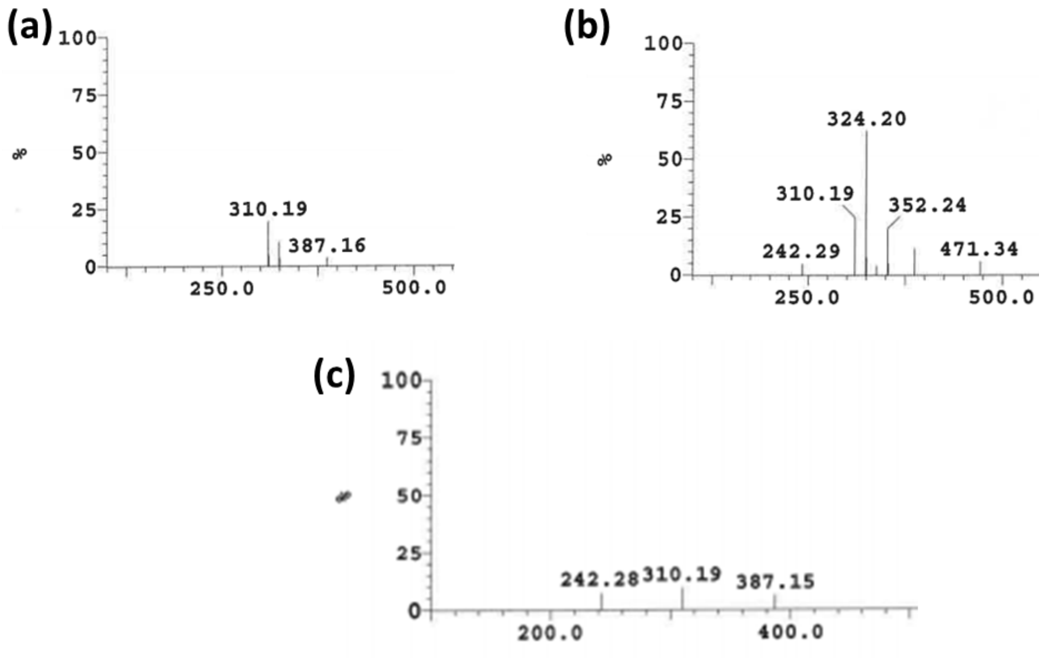

Figure S5: MS of ethanolic extract of **(a)** *Serratia* WT, **(b)** *Serratia* ΔpigB pHDB4 (expressing PigB)**, (c)** *Serratia* ΔpigB pHDB1 (expressing HapB). The peak at 310 has *m/z* 310.19 (calc. M+H$^+$ for norprodigiosin **2**, 310.1914). The peak at 324 has *m/z* 324.2030 (calc. M+H$^+$ for prodigiosin **1,** 324.2070).

Chromatograms

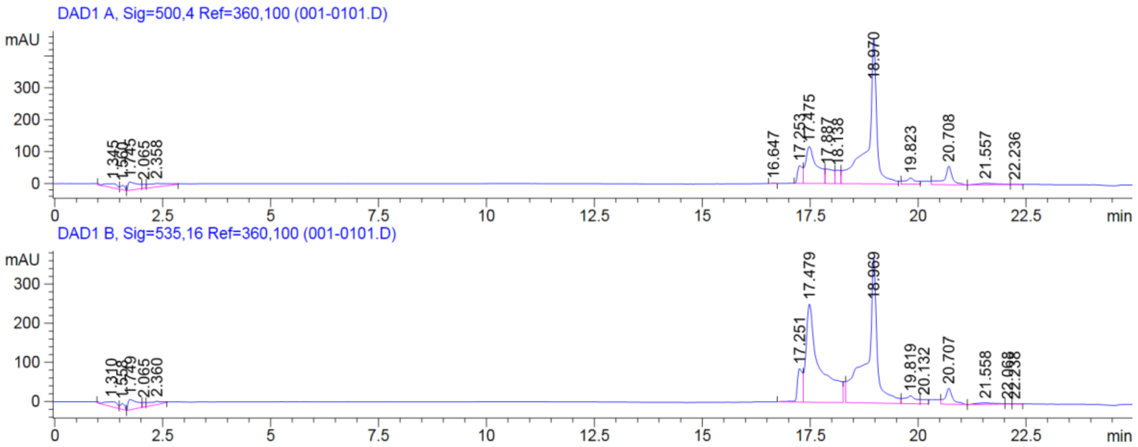

Figure S6: HPLC trace of an ethanolic extract from *Serratia* WT, UV detection at 500 nm (top) and 535 nm (bottom); MS analysis showed that the peak at 17.5 min was norprodigiosin and the one at 18.9 was prodigiosin

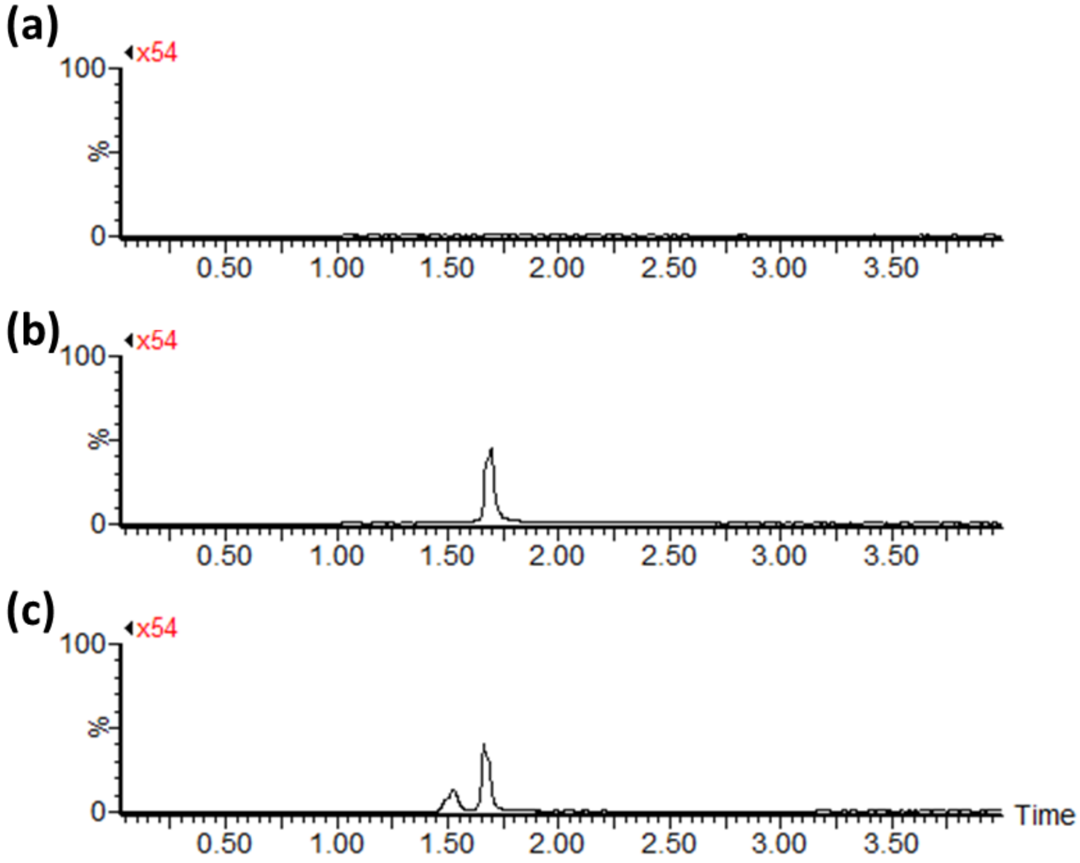

Figure S7: Comparison of synthetic and biological samples of H_2MAP: extracted ions traces at 154.16 (H_2MAP+H^+ requires 154.16) **(a)** extract from *Serratia* ΔpigD, **(b)** extract from *Serratia* ΔpigB, **(c)** synthetic sample of H_2MAP **5**.

Purification of Prodigiosin Analogues

Prodiginine samples were prepared from 1 L of culture of Serratia ΔpigD complemented with **5e** or **5h**. The ethanolic extracts were sterile-filtered (0.22 µm pores) and concentrated under reduced pressure to dryness. The residue was resuspended in 1 ml of H_2O/CH_3CN and purified by HPLC (Agilent Zorbax SB-C18, flow: 3 ml/min, gradient 5 to 100 % CH_3CN in water over 28 min then 100% for 2 min). Fractions containing the prodiginine were concentrated under reduced pressure and resuspended in acidified EtOH (50 µl). 50 µl of H_2O/ CH_3CN (50 µl) was added and the sample was analysed by LC-MS on a Waters Xevo G2-S. HRMS (ESI): *m/z* calcd for $C_{21}H_{27}N_3O{+}H^+$: 338.2227 [*M*+H]$^+$; found: 338.2224 for prodiginine **E** and 338.2228 for prodiginine **H**.

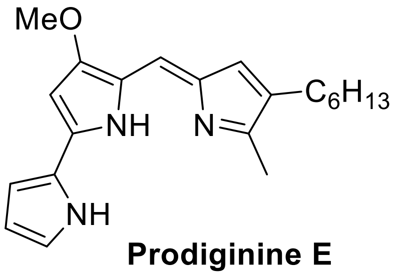

Prodiginine E

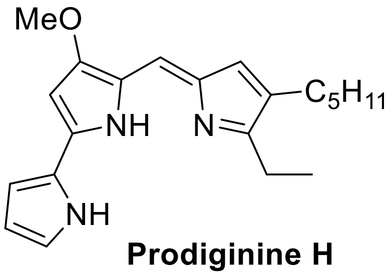

Prodiginine H

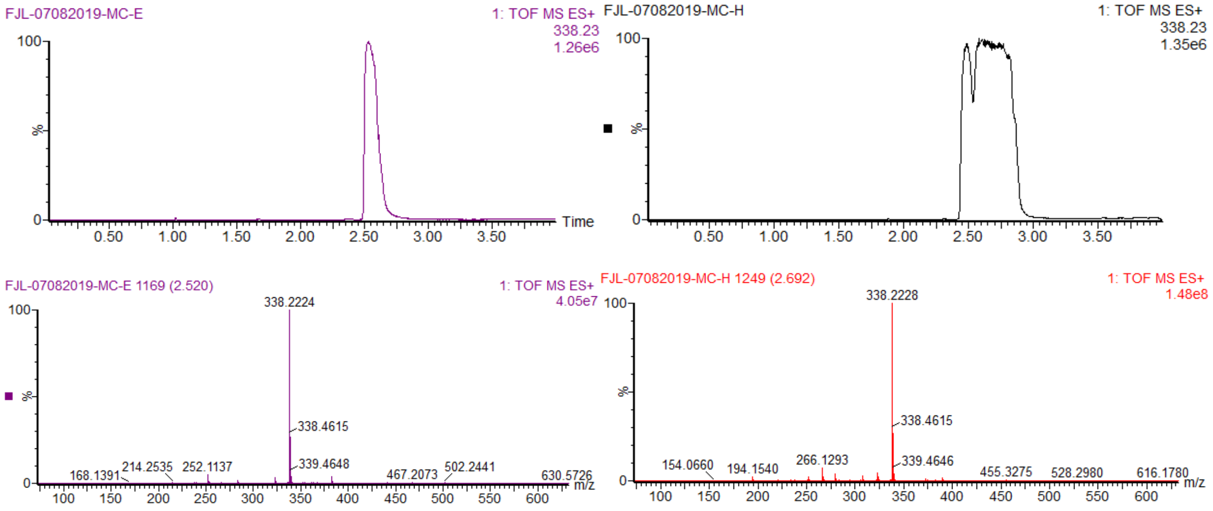

Figure S8: LC-MS of the purified prodigiosin analogues **E** and **H**. Extracted ion chromatogram for *m/z* 338.23 (top) and mass spectrum of peak (bottom) for prodiginine **E** (left) and **H** (right).

Substrate specificity assays

Agar feeding assays

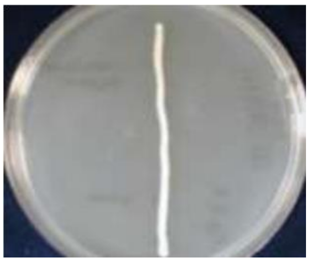

Figure S9: *Serratia ΔpigB* supplemented with H_2MAP shows negligible formation of prodigiosin.

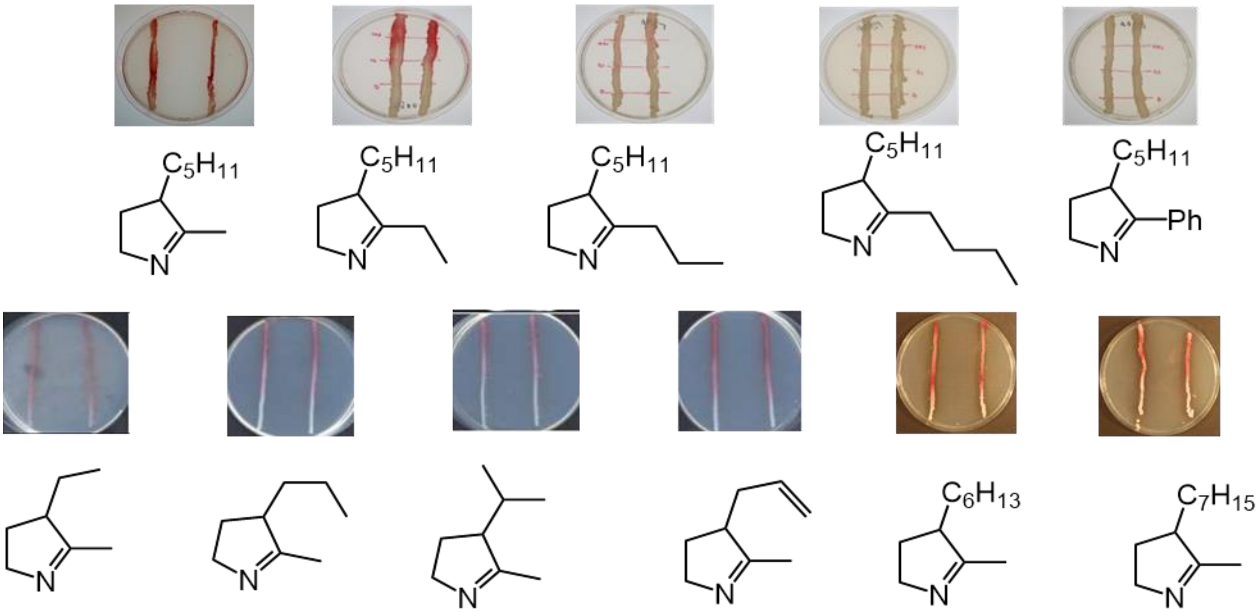

Figure S10: Results of agar plate feeding assay using compounds **5a-k**.

Determination of the extinction coefficient for Ehrlich's assay

MAP was synthesized following the procedure described by Williamson et al.[2] Ehrlich's assay was performed on solution of MAP ranging between 0.1 and 0.8 mM, leading to the results shown in Fig. S8. As expected, a linear increase was obtained. The assay was performed in a 96 well microplate, with 50 µl of MAP solution, 50 µl of stop reagent and 100 µl of Ehrlich's reagent.

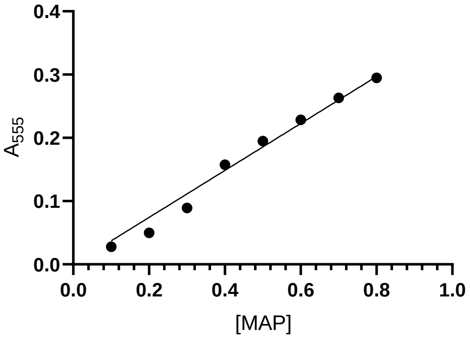

Figure S11: Standard curve for calculation of ε_{555} in the Ehrlich's assay. MAP concentration is in mM.

Synthesis

3-Pentyl-1-vinylpyrrolidin-2-one 8a

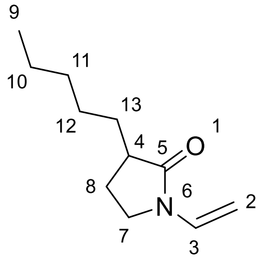

A solution of N-vinylpyrrolidin-2-one **7** (5 ml, 50 mmol, 1 eq.) in dry THF (20 ml) was added dropwise to a stirred solution of NaHDMS (2 M in THF, 26 ml, 52 mmol, 1.04 eq.) at -78 °C under N_2. After 1 h a solution of 1-iodopentane (7.15 ml, 55 mmol, 1.1 eq.) in dry THF (20 ml) was added dropwise. The mixture was allowed to warm to room temperature (RT), stirred for 16 h, diluted with diethyl ether (20 ml), filtered and evaporated under reduced pressure. Purification by column chromatography (petroleum ether/EtOAc, 85:15) gave **8a** (5.6 g, 30 mmol, 60%) as a colourless oil. ^{1}H NMR (400 MHz, $CDCl_3$): δ 0.89 (3H, t, *J* 6.9 Hz, H_9), 1.20-1.40 (7H, m, H_{10}, H_{11}, H_{12}, H_{13a}), 1.74 (1H, m, H_{8a}), 1.87 (1H, m, H_{13b}), 2.28 (1H, *m,* H_{8b}), 2.50 (1H, m, H_4), 3.38 (1H, m, H_{7a}), 3.49 (1H, td, *J* 9.7 & 3.5 Hz, H_{7b}), 4.38 (1H,d, *J* 16 Hz, H_{2a}), 4.42 (1H,d, *J* 9.0 Hz, H_{2b}), 7.10(1H, dd, *J* 16 & 9 Hz, H_3); ^{13}C NMR (100 MHz, $CDCl_3$): δ 14.02 (C_9), 22.52 (C_{10}), 24.41 (C_8), 26.83 (C_{12}), 31.11 (C_{13}), 31.71 (C_{11}), 42.44 (C_4), 42.88 (C_7), 93.97 (C_2), 129.63 (C_3), 175.32 (C_5); IR (neat): ν_{max} 2954m, 2928m, 2858m, 1704s (C=O) cm^{-1}; HRMS: *m/z* calcd for $C_{11}H_{19}NO+H^+$: 182.1545; found: 182.1551.

3-Ethyl-1-vinylpyrrolidin-2-one 8b

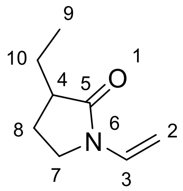

Product **8b** (17 mmol, 70%) was obtained by a similar procedure as for **8a**. ^{1}H NMR δ_H (400 MHz, $CDCl_3$): 0.97 (3H, t, *J* 7.5 Hz, H_9), 1.45 (1H, m, H_{10a}), 1.75 (1H, m, H_{8a}), 1.90 (1H, m, H_{10b}), 2.28 (1H, *m,* H_{8b}), 2.46 (1H, m, H_4), 3.39 (1H, m, H_{7a}), 3.49 (1H, td, *J* 9.6 & 3.4 Hz, H_{7b}), 4.38 (1H, d, *J* 16 Hz, H_{2a}), 4.42 (1H,d, *J* 9 Hz, H_{2b}), 7.10 (1H, dd, *J* 16 & 9 Hz, H_3); ^{13}C NMR (100 MHz, $CDCl_3$): 11.40 (C_9), 23.80 (C_8), 24.14 (C_{10}), 42.88 (C_4), 43.78 (C_7), 94.02 (C_2), 129.60 (C_3), 175.13 (C_5); IR (neat): ν_{max} 2954m, 2928m, 2858m, 1704s (C=O) cm^{-1}; HRMS: *m/z* calcd for $C_8H_{13}NO+H^+$: 140.1070; found 140.1068.

3-Propyl-1-vinylpyrrolidin-2-one 8c

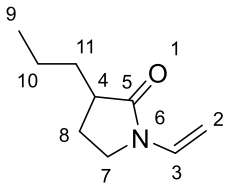

Product **8c** (15 mmol, 60%) was obtained by a similar procedure as for **8a**. ^{1}H NMR (400 MHz, $CDCl_3$): 0.94 (3H, t, 7.2 Hz, H_9), 1.33-1.46 (3H, m, H_{10}, H_{11a}), 1.74 (1H, m, H_{8a}), 1.86 (1H, m, H_{11b}), 2.28 (1H, *m,* H_{8b}), 2.51 (1H, m, H_4), 3.37 (1H, m, H_{7a}), 3.49 (1H, td, *J* 9.6 & 3.5 Hz, H_{7b}), 4.38 (1H, d, *J* 16 Hz, H_{2a}), 4.42 (1H, d, *J* 9 Hz, H_{2b}), 7.10(1H, dd, *J* 16 & 9 Hz, H_3); ^{13}C NMR (100 MHz, $CDCl_3$): 13.95 (C_9), 20.36 (C_{10}), 24.40 (C_8), 33.28 (C_{11}), 42.21 (C_4), 42.88 (C_7), 93.97 (C_2), 129.62 (C_3), 175.32 (C_5); IR (neat): ν_{max} 2956m, 2928m, 2860m, 1702s (C=O) cm^{-1}; HRMS: *m/z* calcd for $C_9H_{15}NO+H^+$: 154.1226; found: 154.1227.

3-Allyl-1-vinylpyrrolidin-2-one 8d

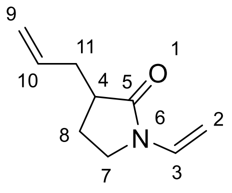

Product **8d** (16 mmol, 64%) was obtained by reacting **7** with allylbromide in the same conditions as for **8a**. ^{1}H NMR (400 MHz, $CDCl_3$): δ 1.80 (1H, m, H_{8a}), 2.23 (2H, m, H_{8b} and H_{11a}), 2.61 (2H, m, H_4 and H_{11b}), 3.39 (1H, m, H_{7a}), 3.49 (1H, td, *J* 9.6 & 3.5 Hz, H_{7b}), 4.38 (1H, d, *J* 16 Hz, H_{2a}), 4.42 (1H,d, *J* 9 Hz, H_{2b}), 5.10 (2H, m, H_9), 5.78 (1H, m, H_{10}) 7.10 (1H, dd, *J* 16 & 9 Hz, H_3); ^{13}C NMR (100 MHz, $CDCl_3$): 23.52 (C_8), 35.27 (C_{11}), 41.90 (C_4), 42.86 (C_7), 94.30 (C_2), 117.20 (C_9), 129.54 (C_3), 135.50 (C_{10}); IR (neat): ν_{max} 2954m, 2928m, 2859m, 1700s (C=O) cm^{-1}; HRMS: *m/z* calcd for $C_9H_{13}NO+H^+$: 152.1070; found: 152.1068.

3-Hexyl-1-vinylpyrrolidin-2-one 8e

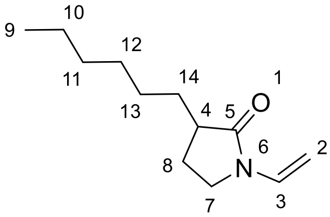

Product **8e** (18 mmol, 70%) was obtained by a similar procedure as for **8a**. ^{1}H NMR (400 MHz, $CDCl_3$): δ 0.87 (3H, t, *J* 5.8 Hz, H_9), 1.26-1.42 (9H, m, H_{10}, H_{11}, H_{12}, H_{13}, H_{14a}), 1.73 (1H, m, H_{8a}), 1.88 (1H, m, H_{14b}), 2.28 (1H, *m,* H_{8b}), 2.49 (1H, m, H_4), 3.37 (1H, m, H_{7a}), 3.49 (1H, td, *J* 9.7 & 3.4 Hz, H_{7b}), 4.38 (1H, d, *J* 16.4 Hz, H_{2a}), 4.42 (1H, d, *J* 9.0 Hz, H_{2b}), 7.10 (1H, dd, *J* 16 & 9 Hz, H_3); ^{13}C NMR (100 MHz, $CDCl_3$): δ 14.07 (C_9), 22.60 (C_{10}), 24.40 (C_8), 27.12 (C_{13}), 29.18 (C_{12}), 31.16 (C_{14}), 31.70 (C_{11}), 42.44 (C_4), 42.88, 93.98 (C_2), 129.62 (C_3), 175.32 (C_5); IR (neat) ν_{max} 2956m, 2928m, 2860m, 1703s (C=O) cm^{-1}; HRMS, *m/z* calcd for $C_{12}H_{21}NO+H^+$: 196.1696; found 196.1694.

3-Heptyl-1-vinylpyrrolidin-2-one 8f

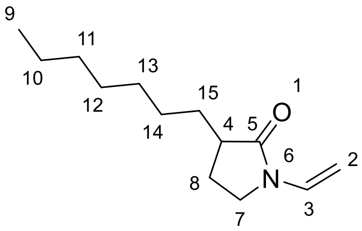

Product **8f** (19 mmol, 73%) was obtained by a similar procedure as for **8a**. ^{1}H NMR (400 MHz, $CDCl_3$): 0.88 (3H, t, *J* 7.1 Hz, H_9), 1.20-1.40 (11H, m, H_{10}, H_{11}, H_{12}, H_{13}, H_{14}, H_{15a}), 1.74 (1H, m, H_{8a}), 1.87 (1H, m, H_{15b}), 2.28 (1H, *m,* H_{8b}), 2.49 (1H, m, H_4), 3.38 (1H, m, H_{7a}), 3.49 (1H, td, *J* 9.7 & 3.2 Hz, H_{7b}), 4.38 (1H,d, *J* 16 Hz, H_{2a}), 4.42 (1H, d, *J* 9.0 Hz, H_{2b}), 7.10 (1H, dd, *J* 16 & 9 Hz, H_3); ^{13}C NMR (100 MHz, $CDCl_3$): δ 14.07 (C_9), 22.64 (C_{10}), 24.40 (C_8), 27.17(C_{14}), 29.17(C_{12}), 29.49 (C_{13}), 31.16 (C_{15}), 31.81 (C_{11}), 42.44 (C_4), 42.88 (C_7), 93.97 (C_2), 129.62 (C_3), 175.32 (C_5); IR (neat): ν_{max} 2954m, 2930m, 2858m, 1705s (C=O) cm^{-1}; HRMS: *m/z* calcd for $C_{13}H_{23}NO+H^+$: 210.1852; found: 210.1853,.

3-Isopropyl-1-vinylpyrrolidin-2-one 8g

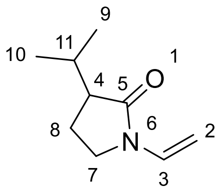

Product **8g** (3.8 mmol, 60%) was obtained by a similar procedure as for **8a**. ^{1}H NMR (400 MHz, $CDCl_3$): δ 0.87 (3H, d, *J* 6.7 Hz, H_9), 1.01 (3H, d, *J* 6.7, H_{10}), 1.87 (1H, m, H_{8a}), 2.10 (1H, m, H_{8b}), 2.23 (1H, m, H_{11}), 2.50 (1H, m, H_4), 3.38 (1H, m, H_{7a}), 3.46 (1H, td, *J* 9.7 & 4.0 Hz, H_{7b}), 4.37 (1H, d, *J* 16 Hz, H_{2a}), 4.42 (1H, d, *J* 9 Hz, H_{2b}), 7.11 (1H, dd, *J* 16 &9 Hz, H_3); ^{13}C NMR (100 MHz, $CDCl_3$): δ 17.71 (C_9), 19.32 (C_8), 20.52 (C_{10}), 28.46 (C_{11}), 43.04 (C_7), 48.20 (C_4), 93.99 (C_2), 129.55 (C_3), 174.65 (C_5); IR (neat): ν_{max} 2954m, 2930m, 2858m, 1704s (C=O) cm^{-1}; HRMS: *m/z* calcd for $C_9H_{15}NO+H^+$: 154.1226; found: 154.1225.

5-Methyl-4-pentyl-3,4-dihydro-2H-pyrrole (H_2MAP) **5a**

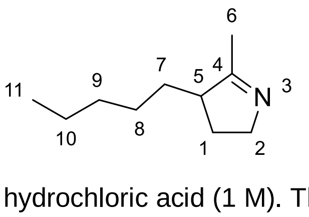

A solution of 3-pentyl-1-vinylpyrrolidin-2-one **8a** (1 g, 5.5 mmol, 1 eq) in Et_2O (2 ml) was added dropwise to a stirred solution of MeLi (1.6 M in Et_2O, 4.5 ml, 7.2 mmol, 1.3 eq) at -10 °C. The mixture was stirred at -10°C for 1 h and at RT for 1 h. It was then cooled to 0 °C and hydrochloric acid (1 M) was added slowly to pH 2. The mixture was stirred at RT for 40 min. The two phases were separated, and the organic phase was extracted with hydrochloric acid (1 M). The combined aqueous phases were washed with DCM. Aq. NaOH (1 M) was added to pH 11 and the mixture was extracted with DCM. The organic layer was dried ($MgSO_4$) and evaporated. Purification by column chromatography (DCM/MeOH, 9:1) gave **5a** (658 mg, 78%) as an oil. ^{1}H NMR (400 MHz, $CDCl_3$) δ 0.87 (3H, t, *J* 6.8, H_{11}), 1.28 (7H, m, H_{7a}, H_8, H_9 and H_{10}), 1.50 (1H, m, H_{1a}), 1.65 (1H, m, H_{7b}), 1.94 (3H, s, H_6), 1.98-2.08 (1H, m, H_{1b}), 2.51-2.65 (1H, m, H_5), 3.54-3.66 (1H, m, H_{2a}), 3.67-3.82 (1H, m, H_{2b}); ^{13}C NMR (125 MHz, $CDCl_3$, APT used for assignments): δ 13.9 (C_{11}), 18.1 (C_6), 22.6 (C_{10}), 27.3 (C_1), 29.1, 31.8 and 31.9 (C_7, C_8 and C_9), 50.7 (C_5), 58.8 (C_2), 177.6 (C_4); IR (neat): ν_{max} 2955m, 2928s, 2860m, 1649s (C=N) cm^{-1}; HRMS: *m/z* calcd for $C_{10}H_{19}N+H^+$: 154.1596; found: 154.1595.

5-Methyl-4-ethyl-3,4-dihydro-2H-pyrrole 5b

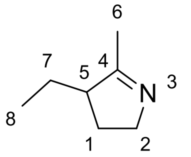

Product **5b** (5.3 mmol, 75%) was obtained by a similar procedure as for **5a**. Purification by column chromatography was not necessary. ^{1}H NMR (400 MHz, $CDCl_3$): δ 0.91 (3H, t, *J* 7.2 Hz, H_8), 1.28 (1H, m, H_{7a}), 1.52 (1H, m, H_{1a}), 1.73 (1H, H_{7b}), 1.97 (3H, s, H_6), 2.05 (1H, m, H_{1b}), 2.59 (1H, m, H_5), 3.64 (1H, m, H_{2a}), 3.74 (1H, m, H_{2b}); ^{13}C NMR (100 MHz, $CDCl_3$): δ 11.73 (C_8), 17.96 (C_6), 24.63 (C_7), 28.52 (C_1), 52.20 (C_5), 58.90 (C_2), 177.94 (C_4); IR (neat): ν_{max} 2957m, 2929s, 2860m, 1649s (C=N) cm^{-1}; HRMS: *m/z* calcd for $C_7H_{13}N+H^+$: 112.1121; found: 112.1121.

5-Methyl-4-propyl-3,4-dihydro-2H-pyrrole 5c

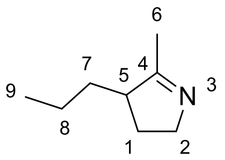

Product **5c** (4.6 mmol, 71%) was obtained by a similar procedure as for **5a**. Purification by column chromatography was not necessary. ^{1}H NMR (400 MHz, $CDCl_3$): δ 0.87 (3H, t, *J* 7.5Hz, H_9), 1.14-1.42 (3H, m, H_8, H_{7a}), 1.50 (1H, m, H_{1a}), 1.65 (1H, m, H_{7b}), 1.97 (3H, s, H_6), 2.05 (1H, m, H_{1b}), 2.62 (1H, m, H_5), 3.62 (1H, m, H_{2a}), 3.74 (1H, m, H_{2b}); ^{13}C NMR (100 MHz, $CDCl_3$): δ 14.10 (C_9), 17.97 (C_6), 20.94 (C_8), 29.12 (C_1), 34.05 (C_7), 50.63 (C_5), 58.78 (C_2), 178.04 (C_4); IR (neat): ν_{max} 2955m, 2929s, 2862m, 1650s (C=N) cm^{-1}; HRMS: *m/z* calcd for $C_8H_{15}N+H^+$: 126.1217; found: 126.1215.

5-Methyl-4-allyl-3,4-dihydro-2H-pyrrole 5d

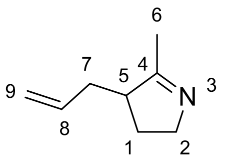

Product **5d** (4.5 mmol, 70%) was obtained by a similar procedure as for **5a**. Purification by column chromatography was not necessary. ^{1}H NMR (400 MHz, $CDCl_3$): δ 1.58 (1H, m, H_{1a}), 2.0 (3H, s, H_6), 2.05 (2H, m, H_{1b}, H_{7a}), 2.42 (1H, m, H_{7b}), 2.74 (1H, m, H_5), 3.70 (2H, m, H_2), 5.06 (2H, m, H_9), 5.72 (1H, m, H_8); ^{13}C NMR (100 MHz, $CDCl_3$): δ 18.07 (C_6), 28.67 (C_1), 36.05 (C_7), 50.09 (C_5), 58.91 (C_2), 116.71 (C_9), 135.74 (C_8), 176.88 (C_4); IR (neat): ν_{max} 2955m, 2927s, 2859m, 1649s (C=N) cm^{-1}; HRMS: *m/z* calcd for $C_8H_{13}N+H^+$: 124.1121; found: 124.1123.

5-Methyl-4-hexyl-3,4-dihydro-2H-pyrrole 5e

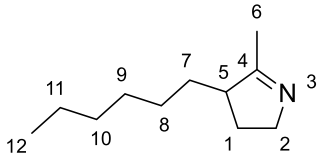

Product **5e** (3.4 mmol, 68%) was obtained by a similar procedure as for **5a**. ^{1}H NMR (400 MHz, $CDCl_3$): δ 0.89 (3H, t, *J* 6.8 Hz, H_{12}), 1.20-1.34 (9H, m, H_{11}, H_{10}, H_9, H_8, H_{7a}), 1.50 (1H, m, H_{1a}), 1.67 (1H, m, H_{7b}), 1.97 (3H, s, H_6), 2.05 (1H, m, H_{1b}), 2.62 (1H, m, H_5), 3.63 (1H, m, H_{2a}), 3.76 (1H, m, H_{2b}); ^{13}C NMR (100 MHz, $CDCl_3$): δ 14.07 (C_{12}), 18.01 (C_6), 22.62 (C_{11}), 27.71 (C_8 or C_9), 29.17 (C_1), 29.35 (C_8 or C_9), 31.77 (C_7), 31.86 (C_{10}), 50.83 (C_5), 58.89 (C_2), 177.88 (C_4); IR (neat): ν_{max} 2957m, 2930m, 2861m, 1649s (C=N) cm^{-1}; HRMS: *m/z* calculated for $C_{12}H_{21}NO+H^+$: 196.1696; found: 196.1694.

5-Methyl-4-heptyl-3,4-dihydro-2H-pyrrole 5f

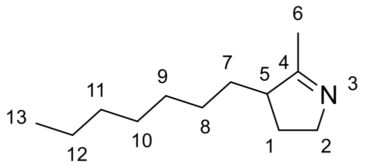

Product **5f** (3.1 mmol, 65%) was obtained by a similar procedure as for **5a**. Purification by column chromatography was not necessary. ^{1}H NMR (400 MHz, $CDCl_3$): δ 0.88 (3H, t, *J* 6.5 Hz, H_{13}), 1.20-1.34 (11H, m, H_{12}, H_{11}, H_{10}, H_9, H_8, H_{7a}), 1.50 (1H, m, H_{1a}), 1.65 (1H, m, H_{7b}), 1.97 (3H, s, H_6), 2.05 (1H, m, H_{1b}), 2.61 (1H, m, H_5), 3.63 (1H, m, H_{2a}), 3.75 (1H, m, H_{2b}); ^{13}C NMR (100 MHz, $CDCl_3$): δ 14.08 (C_{13}), 17.99 (C_6), 22.64 (C_{12}), 27.7 (C_8), 29.14 (C_1), 29.22 (C_{10}), 29.64 (C_9), 31.83 & 31.84 (C_7 and C_{11}), 50.82 (C_5), 58.84 (C_2), 177.96 (C_4); IR (neat): ν_{max} 2955m, 2928s, 2860m, 1650s (C=N) cm^{-1}; HRMS: *m/z* calcd for $C_{12}H_{23}N+H^+$: 182.1903; found: 182.1906.

5-Methyl-4-isopropyl-3,4-dihydro-2H-pyrrole 5g

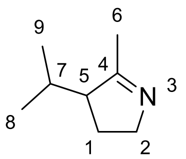

Product **5g** (3.9 mmol, 60%) was obtained by a similar procedure as for **5a**. ^{1}H NMR (400 MHz, $CDCl_3$): δ 0.71 (3H, d, *J* 5.5 Hz, H_8), 0.96 (3H, d, *J* 6.6 Hz, H_9), 1.66 (H, m, H_{1a}), 1.84 (1H, m, H_{1b}), 1.97 (3H, s, H_6), 2.10 (1H, m, H_7), 2.69 (1H, m, H_5), 3.69 (2H, m, H_2); ^{13}C NMR (100 MHz, $CDCl_3$): δ 16.3 (C_8), 18.2 (C_6), 21.7 (C_9), 22.9 (C_1), 28.0 (C_7), 57.2 (C_5), 59.54 (C_2); IR (neat): ν_{max} 2956m, 2928s, 2860m, 1650s (C=N) cm^{-1}; HRMS: *m/z* calcd for $C_8H_{15}N+H^+$: 126.1217; found: 126.1216.

5-Ethyl-4-pentyl-3,4-dihydro-2H-pyrrole 5h

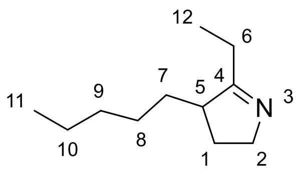

Product **5f** (0.17 mmol, 17%) was obtained by a similar procedure as for **5a**. ^{1}H NMR (400 MHz, $CDCl_3$): δ 0.87 (3H, t, *J* 6.6, H_{11}), 1.13 (3H, t, *J* 7.5, H_{12}), 1.17-1.36 (7H, m, H_{7a}, H_8, H_9 and H_{10}), 1.50 (1H, m, H_{7b}), 1.64 (1H, m, H_{6a}), 2.04 (1H, m, H_{6b}), 2.23 (1H, m, H_{1a}), 2.34 (1H, m, H_{1b}), 2.66 (1H, m, H_5), 3.65 (1H, m, H_{2a}), 3.76 (1H, m, H_{2b}); ^{13}C NMR (100 MHz, $CDCl_3$): δ 10.7 (C_{12}) 14.2 (C_{11}), 22.8 (C_{10}), 25.0 (C_6), 27.7 (C_1), 29.3, 32.0, 32.1 (C_7, C_8 and C_9), 49.9 (C_5), 58.9 (C_2), 182.4 (C_4); LC/MS: *m/z* for $C_{11}H_{21}N+H^+$:168.17 ; found.168.3

5-Propyl-4-pentyl-3,4-dihydro-2H-pyrrole 5i

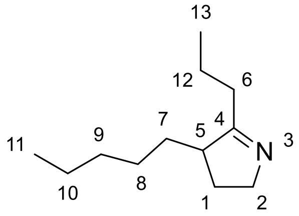

To a stirred solution of iodopropane (52 μl, 0.55 mmol, 1 eq) dry n-pentane-Et_2O (3:2, 5 ml) under N_2 at -78 °C was added dropwise ^{t}BuLi (1.7 M in hexane, 0.7 ml 1.21 mmol, 2.2 eq). The mixture was stirred at -78°C for 5 min and at RT for 1 h, then cooled again to -78 °C. A solution of **8a** (200 mg, 1.1 mmol, 2 eq) in dry Et_2O (0.4 ml) was added dropwise. The mixture was stirred for 1 h at -10 °C and then 1.5 h at room temperature. Hydrochloric acid (1 M) was then added dropwise to pH 2. After being stirred for 20 min, the organic layer was separated and extracted with hydrochloric acid (0.1 M). The combined aqueous layers were washed with Et_2O. Aq. NaOH (1 M) was then added to pH 10 and mixture was extracted with DCM. The combined extracts were dried ($MgSO_4$) and evaporated. Purification by column chromatography (DCM/MeOH, 98:2) gave **5g** (10 mg, 55 µmol, 10%). ^{1}H NMR (400 MHz, $CDCl_3$): δ 0.87 (3H, m, H_{11}), 0.93 (3H, t, *J* 7.3 , H_{13}), 1.26-1.39 (11H, m, H_6, H_{7a}, H_8, H_9 , H_{10}, and H_{12}), 2.13-2.34 (3H, m, H_1 and H_{7b}), 2.63 (1H, m, H_5), 3.57-3.69 (1H, m, H_{2a}), 3.69-3.81 (1H, m, H_{2b}); ^{13}C NMR (125 MHz, $CDCl_3$): δ 14.1 (C_{13}) 14.1 (C_{11}), 21.1 (C_1), 22.5 (C_{10}), 22.6 (C_{12}), 27.5 ,30.8, 31.7, 31.9 (C_6, C_7, C_8 and C_9), 40.2 (C_5), 60.2 (C_2), 171.1 (C_4); LC/MS: *m/z* calcd for $C_{12}H_{23}N+H^+$:182.19 ; found.182.3.

5-Butyl-4-pentyl-3,4-dihydro-2H-pyrrole 5j

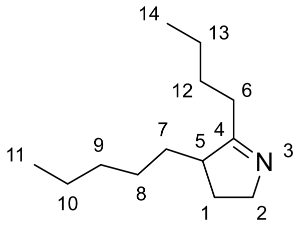

Product **5h** (66 mmol, 33%) was obtained by a similar procedure as for **5a**. ^{1}H NMR (400 MHz, $CDCl_3$): δ 0.90 (6H, m, H_{11} and H_{14}), 1.13-1.42 (9H, m, H_6, H_{7a}, H_8, H_9, H_{10} and H_{13}), 1.13-1.73 (4 H, m, H_6 and H_{12}), 2.02 (1H, m, H_{7b}), 2.19 (1H, m, H_{1a}), 2.28 (1H, m, H_{1b}), 2.62 (1H, m, H_5), 3.64 (1H, m, H_{2a}), 3.75 (1H, m, H_{2b}); ^{13}C NMR (100 MHz, $CDCl_3$): δ 13.9 (C_{11}), 14.1 (C_{14}), 22.6 (C_{10}), 22.7 (C_{13}), 27.5 (C_1), 28.4, 29.0, 31.4, 31.8 and 31.9 (C_6, C_7, C_8, C_9 and C_{12}), 49.8 (C_5), 58.8 (C_2), 181.0 (C_4); LC/MS: *m/z* calcd for $C_{13}H_{25}N+H^+$:196.21 ; found.196.3.

5-Phenyl-4-pentyl-3,4-dihydro-2H-pyrrole 5k

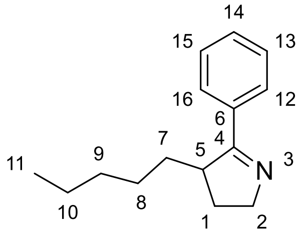

Product **5i** (1.5 mmol, 75%) was obtained by a similar procedure as for **5a.** ^{1}H NMR (400 MHz, $CDCl_3$): δ 0.84 (3H, t, *J* 6.8, H_{11}), 1.19–1.34 (6H, m, H_8, H_9 and H_{10}), 1.64 (2H, m, H_7),1.82 (1H, m, H_{1a}), 2.14 (1H, m, H_{1b}), 3.33 (1H, m, H_5), 3.92 (1H, m, H_{2a}), 4.03 (1H, m, H_{2b}), 7.39 (3H, m, H_{13}, H_{14} and H_{15}), 7.77 (2H, m, H_{12} and H_{16}); ^{13}C NMR (100 MHz, $CDCl_3$): δ 14.0 (C_{11}), 22.6 (C_{10}), 27.5 (C_1), 29.1, 31.8, 32.0 (C_7, C_8 and C_9), 47.3 (C_5), 58.8 (C_2), 127.8 (2C, C_{12} and C_{16}), 128.4 (2C, C_{13} and C_{15}), 130.0 (C_{14}) 176.7 (C_4); LC/MS: *m/z* calcd for $C_{15}H_{21}N+H^+$:216.17 ; found.216.3

Ethyl 2-acetylheptanoate 10

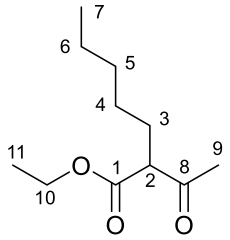

Dry EtOH (6 ml) was added slowly and carefully to sodium metal (177 mg, 8.68 mmol) under argon. After the initial vigorous reaction, the mixture was heated to 60°C until the sodium had reacted completely. Ethyl acetoacetate (1 g, 7.68 mmol) was then added over 10 min followed by iodopentane (1.82 g, 9.22 mmol). The solution was heated at reflux for 4 h, then cooled and evaporated. The residue was partitioned between DCM and water and the combined organic layers were dried ($MgSO_4$) and evaporated. The residue was purified by flash chromatography on silica eluting with 3% EtOAc in hexane to give **10** (1.37 g, 88%) as a colourless oil. R_f 0.50 (acetone:hexane; 2:9); ^{1}H NMR (400 MHz, $CDCl_3$): δ 0.83 (3H, t, J 7.0, H_7), 1.19–1.28 (9H, m, H_4, H_5, H_6 and H_{11}), 1.79 (2H, m, H_3), 2.17 (3H, s, H_9), 3.35 (1H, t, *J* 7.4 Hz, H_2), 4.18 (2H, q, *J* 7.0 Hz, H_{11}); ^{13}C NMR (100 MHz, $CDCl_3$): δ 13.8 (C_7), 14.0 (C_{11}), 22.2 (C_6), 26.9 and 28.1 (C_3 and C_4), 28.6 (C_9), 31.4 (C_5), 60.13 (C_2), 61.40 (C_{10}), 170.12 (C_1), 203.51 (C_8); IR (neat): ν_{max} 2958m, 2932m, 2862m, 1738s (ester C=O), 1715s (ketone C=O), 1193m cm^{-1}; HRMS: *m/z* calcd for $C_{11}H_{20}O_3+Na^+$: 223.1305; found: 223.1294.

Ethyl 2-acetyl-2-allylheptanoate 11

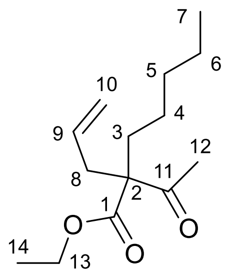

Ethyl 2-acetylheptanoate, **10** (1 g, 4.975 mmol) was added dropwise to a solution of sodium ethoxide at 60 °C (prepared as above, 138 mg of sodium in 5 ml of dry ethanol). After few minutes, allyl bromide (0.54 ml, 6.219 mmol) was added dropwise over 2 min and the mixture was heated at reflux for 2 h, then filtered and evaporated under reduced pressure. The residue was partitioned between DCM and water, and the organic layer was dried ($MgSO_4$) and evaporated to give **11** (871 mg, 73%) as a colourless oil. Rf 0.51 (acetone:hexane; 2:9); ^{1}H NMR (400 MHz, $CDCl_3$): δ 0.83 (3H, t, *J* 7.0, H_7), 0.98-1.17 (2H, m, H_6), 1.20-1.32 (7H, m, H_4, H_5 and H_{14}), 1.73-1.89 (2H, m, H_3), 2.08 (3H, s, H_{12}), 2.49-2.63 (2H, m, H_8), 4.18 (2H, q, *J* 7.2, H_{13}), 5.01 (1H, ddt, *J* 17.0, 4.0 & 1.2, trans-H_{10}), 5.05 (1H, ddd, *J* 10.4, 4.0 & 1.2, cis-H_{10}), 5.55 (1H, ddt, *J* 17.0, 10.4 & 7.2 Hz, H_9); ^{13}C NMR (100 MHz, $CDCl_3$): δ 13.86 (C_7), 14.04 (C_{14}), 22.27 (C_6), 23.20 (C_4 or C_5), 26.69 (C_{12}), 31.23 (C_3), 32.02 (C_8), 35.77 (C_4 or C_5), 61.14 (C_{13}), 63.35 (C_2), 118.58 (C_{10}), 132.55 (C_9), 172.02 (C_1), 204.70 (C_{11}); IR (neat): ν_{max} 3081w (vinyl C-H), 2958m, 2931m, 2863w, 1738m (ester C=O), 1712s (ketone C=O), 918m (vinyl C=C) cm^{-1}; HRMS: *m/z* calcd for $C_{14}H_{25}O_3+H^+$: 241.1804; found: 241.1806.

3-Allyloctan-2-one 12

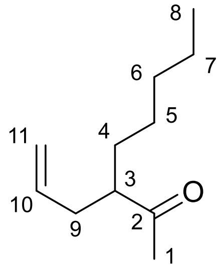

A solution of **11** (0.5 g, 0.208 mmol) in 5% aq. NaOH (5 ml) and MeOH (1 ml) was heated at 80 °C for 24 h and then cooled to 0 °C, acidified to pH 5-6 with sulphuric acid and extracted with DCM. The combined organic layers were dried ($MgSO_4$) and evaporated. The residue was purified by flash chromatography on silica eluting with EtOAc-hexane (1:5) to give **12** (311 mg, 89%) as oil; R_f 0.56 (acetone:hexane; 2:9); ^{1}H NMR (400 MHz,$CDCl_3$): δ 0.82 (3H, t, *J* 7.0, H_8), 1.15–1.26 (6H, m, H_5, H_6 and H_7), 1.38 (1H, m, H_{4a}), 1.54 (1H, m, H_{4b}), 2.06 (3H, s, H_1), 2.13 (1H, m, H_{9a}), 2.27 (1H, m, H_{9b}), 2.49 (1H, tt, *J* 6.0 & 8.0 Hz, H_3), 4.94 (1H, ddt, *J* 17.0, 4.0 & 1.2, trans-H_{11}), 4.97 (1H, ddd, *J* 10.4, 4.0 & 1.2 Hz, cis-H_{11}), 5.65 (1H, ddt, *J* 17.0, 10.4 & 7.2 Hz, H_{10}); ^{13}C NMR (100 MHz, $CDCl_3$): δ 14.56 (C_8), 23.06 (C_7), 27.50, 29.71, 31.74 and 32.48 (C_1, C_4, C_5 and C_6), 36.35 (C_9), 53.31 (C_3), 117.20 (C_{11}), 136.27 (C_{10}), 212.62 (C_2); IR (neat): ν_{max} 3080w (C=C-H), 2956m, 2929m, 2860m, 1703s (C=O) cm^{-1}; HRMS: *m/z* calcd for $C_{11}H_{21}O+H^+$: 169.1587; found: 169.1592.

3-Allyloctan-2-ol 13

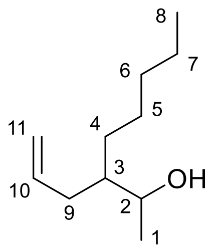

$NaBH_4$ (0.562 g, 14.87 mmol) was added in portions to a stirred solution of **12** (1 g, 5.95 mmol) in dry methanol (8 ml) at 0 °C under argon. The mixture was stirred for 1 h at 0 °C and 1 hr at RT, then poured into a mixture of saturated aqueous NH_4Cl (50 ml) and DCM (50 ml). The organic phase was separated, washed with water, dried ($MgSO_4$), and evaporated to give the 1:1 mixture of diastereoisomers **13** (992 mg, 98%) as oil, R_f 0.38 (acetone:hexane; 2:9); ^{1}H NMR (400 MHz, CD_3OD): δ 0.91 (3H, t, *J* 7.1 Hz, H_8), 1.12 & 1.13 (3H, 2 x d, *J* 6.1 Hz, H_1), 1.27-1.44 (9H, m, H_3, H_4, H_5, H_6 and H_7), 1.96 (0.5H, dt, *J* 14.2, 7.3 Hz), 2.13 (1H, m) & 2.27 (0.5H, dt, *J* 14.2, 5.8 Hz, H_9), 3.77 (1H, m, H_2), 4.94-5.06 (2H, m, H_{11}), 5.81 (1H, m, H_{10}); ^{13}C NMR (100 MHz, CD_3OD): δ 14.6 (C_8), 19.9 & 20.1 (C_1), 23.8 (C_7), 28.2 & 28.4, 30.3 & 30.4, 33.5 & 33.6 (C_4, C_5 and C_6), 35.4 & 35.5 (C_9), 46.09 & 46.12 (C_3), 69.7 (C_2), 116.2 & 116.3 (C_{11}), 139.1 (C_{10}); IR (neat): ν_{max} 3361br (O-H), 2958m, 2926s, 2859m cm^{-1}; HRMS: *m/z* calcd for $C_{11}H_{23}O+H^+$: 171.1743; found: 171.1746.

4-(1-Phthalimidoethyl)non-1-ene 14

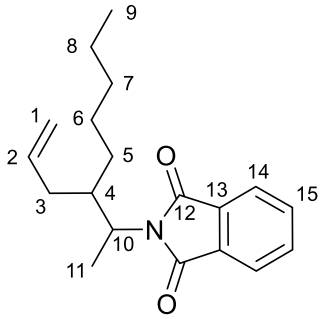

Diethyl azodicarboxylate (930 mg, 5.34 mmol) was added dropwise in the dark to a solution of alcohol **13** (700 mg, 4.11 mmol), phthalimide (786 mg, 5.34 mmol), and PPh_3 (1.4 g, 5.34 mmol) in anhydrous THF (5 ml) at 0°C. After stirring at this temperature for 1 hr the reaction mixture was allowed to warm to RT and stirred for further 13 hr. The reaction mixture was poured into water (20 ml) and the product was extracted with EtOAc (2x25 ml). The combined organic extracts were washed with brine, dried over $MgSO_4$, and filtered. The solvent was evaporated under reduced pressure and the resulting crude product was purified over silica column chromatography (acetone:hexanes; 0.4:9.6) to furnish the diastereoisomeric mixture (*ca.* 1:1) of **14** (530 mg, 43%) as a colourless oil. R_f 0.63 (acetone:hexane; 2:3); ^{1}H NMR (400 MHz, $CDCl_3$): δ 0.70 & 0.82 (3H, 2 x t, *J* 7.1 Hz, H_9), 0.98-1.17 (4H, m) & 1.17-1.35 (4H, m, H_5, H_6, H_7 and H_8), 1.40 & 1.41 (3H, 2 x d, *J* 7.0, H_{11}), 1.87-2.03 (1H, m), 2.03-2.14 (0.5H, m) & 2.20-2.31 (1.5H, m, H_4 & H_5), 4.13 (2H, m, H_{10}), 4.72-4.82 & 4.97-5.06 (2H, 2 x m, H_2), 5.59 (0.5H, ddt, *J* 17, 10 & 7 Hz) & 5.75 (0.5H, m, H_2), 7.60-7.65 (2H, m, H_{15}), 7.71-7.77 (2H, m, H_{14}); ^{13}C NMR (100 MHz, $CDCl_3$): δ 14.0 & 14.1 (C_9), 16.7 & 17.0 (C_{11}), 22.5 & 22.7, 25.3 & 25.7, 29.6 & 30.1, 32.1 & 32.3 and 34.0 & 35.1 (C_3, C_5, C_6,C_7 and C_8), 39.7 & 39.9 (C_4), 50.10 & 50.14 (C_{10}), 116.1 & 116.9 (C_1), 123.10 & 123.12 (C_2), 132.0 & 132.1 (C_{13}), 133.82 & 133.86 (C_{15}), 135.3 & 136.3 (C_{14}), 168.62 & 168.67 (C=O); I.R. (neat): ν_{max} 3080w (C=C-H), 2952m, 2929s, 2859m, 1705s (C=O) cm^{-1}; HRMS: *m/z* calcd for $C_{19}H_{25}NO_2+Na^+$: 322.1778; found: 322.1781; elemental analysis calcd (%) for $C_{19}H_{25}NO_2$: C 76.22, H 8.42, N, 4.68; found: C 76.22, H 8.41, N, 4.63.

3-(1-Phthalimidoethyl)octanal 15

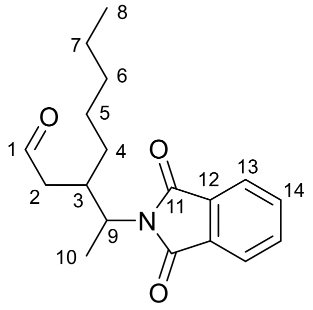

A solution of **14** (500 mg, 1.55 mmol) in DCM (15 ml) at -78 °C was bubbled with ozone/oxygen until the solution turned blue (20 min), at which point the ozonizer was stopped and oxygen was bubbled through the solution until the blue colour had disappeared. The mixture was poured into a stirred suspension of Zn powder (1.5 g) in DCM (15 ml) at 0 °C. Glacial acetic acid (1 ml) was added dropwise to the mixture maintaining the temperature below 10 °C. The mixture was then allowed to warm to RT, stirred for 2 h, and then then filtered through Celite. The filtrate was washed with aq. $NaHCO_3$ and then brine, dried over $MgSO_4$ and evaporated. Purification by silica flash chromatography (acetone:hexane; 3:97) gave the aldehyde **14** (352 mg, 70%) as a mixture of diastereoisomers (*ca.* 5:3) as a colourless oil. R_f 0.53 (acetone:hexane; 2:3); ^{1}H NMR (400 MHz, $CDCl_3$): δ 0.75 (3H, t, *J* 6.9 Hz, $H_{8'}$), 0.84 (3H, t, *J* 6.9, H_8), 1.05-1.40 (32H, m, H_4, H_5, H_6, H_7 both isomers), 1.43 (3H, d, *J* 7.0 Hz, $H_{10'}$),1.47 (3H, d, *J* 7.0 Hz, H_{10}), 2.30 (1H, ddd, *J* 16.9, 6.7 & 2.2 Hz, H_{2a}), 2.38 (1H, ddd, *J* 16.9, 5.6 & 2.3 Hz, H_{2b}), 2.41 (1H, ddd, *J* 17.1, 6.6 & 2.1 Hz, $H_{2a'}$), 2.61 (1H, ddd, *J* 17.1, 5.5 & 1.5 Hz, $H_{2b'}$), 2.66-2.79 (2H, m, H_3 and $H_{3'}$), 4.23 (1H, dq, *J* 9.5 & 7.0 Hz, H_9), 4.28 (1H, dq, *J* 8.2 & 7.1 Hz, $H_{9'}$), 7.67 (4H, m, H_{14} and $H_{14'}$), 7.77 (4H, m, H_{13} and $H_{13'}$), 9.57 (1H, t, *J* 2.2 Hz, H_1), 9.75 (1H, t, *J* 1.8 Hz, $H_{1'}$); ^{13}C NMR (100 MHz, $CDCl_3$): δ 14.05 & 14.15 (C_8), 15.9 & 16.9 (C_{10}), 22.6 & 22.7, 25.7 & 26.05, 31.6 & 31.8, 32.0 & 32.2 and 36.3 & 36.4 (C_3, C_4, C_5,C_6 and C_7), 45.6 & 46.0 (C_2), 50.2 & 50.4 (C_9), 123.33 & 123.37 (C_{14}), 131.9 & 132.0 (C_{12}), 134.13 & 134.17 (C_{13}), 168.7 (C_{11}), 201.90 & 201.95 (C_1); I.R. ν_{max} (neat) 2956m, 2928s, 2858m, 1711s (CH=O), 1701s (N-C=O) cm^{-1}; HRMS: *m/z* calcd for $C_{18}H_{23}NO+Na^+$: 324.1570; found: 324.1582; elemental analysis calcd (%) for $C_{18}H_{23}NO_3$: C 71.73, H 7.69, N 4.65; found: C 71.75, H 7.66, N 4.76.

2-Methyl-3-pentyl-3,4-dihydro-2H-pyrrole 6

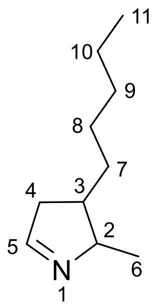

To a degassed solution of phthalimide **14** (70 mg, 0.53 mmol) in absolute EtOH (3 ml) at RT under argon was added $MeNH_2$ (33% in absolute EtOH; 0.3 ml, 2.32 mmol). The mixture was heated for 4 h at 70 °C, then diluted with an equal volume of water, acidified (dil. HCl) to pH 2, and washed with Et_2O. The aqueous layer was basified with solid KOH to pH>10 and extracted with Et_2O. The combined extracts were washed with brine, dried ($MgSO_4$), and evaporated under reduced pressure carefully so as not lose the volatile product. The resulting residue was either passed through a silica column (acetone:hexane 3:97) or distilled using Kugelrohr apparatus to give the diastereo-isomeric mixture (*ca.* 5:3) of dihydroMAP **6** (29 mg, 79%) as a colourless oil. R_f 0.35 (acetone:hexane 3:7); ^{1}H NMR (400 MHz, CD_3COCD_3): δ 0.89 (6H, t, *J* 7.0 Hz, H_{11} and $H_{11'}$), 0.98 (3H, d, *J* 7.0, $H_{6'}$), 1.18 (3H, d, *J* 7.0 Hz, H_6), 1.22-1.36 (16H, m, H_7, H_8, H_9, H_{10} both isomers),1.45 (1H, m, H_3), 1.66 (1H, m, $H_{3'}$), 2.08-2.22 (2H, m, H_{4a} and $H_{4a'}$), 2.56 (1H, m, $H_{4b'}$), 2.74 (1H, m, H_{4b}), 3.56 (1H, m, H_2), 4.02 (1H, m, $H_{2'}$), 7.38 (1H, br s, H_5), 7.44 (1H, br s, $H_{5'}$); ^{13}C NMR (100 MHz, CD_3COCD_3): δ 14.4 (C_{11}), 15.5 & 21.85 (C_6), 23.3, 28.9, 29.3, 29.6, 30.4, 32.8, 32.9 and 35.4 (C_4, C_7, C_8, C_9 and C_{10}), 44.0 & 44.5 (C_3), 69.8 & 74.9 (C_2), 164.6 & 165.2 (C_5); I.R. (neat): ν_{max} 2957m, 2923s, 2856m, 1644m (C=N) cm^{-1}; HRMS: *m/z* calcd for $C_{10}H_{20}N+H^+$ 154.1596; found 154.1590.

NMR spectra

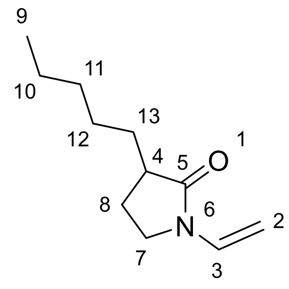

8a

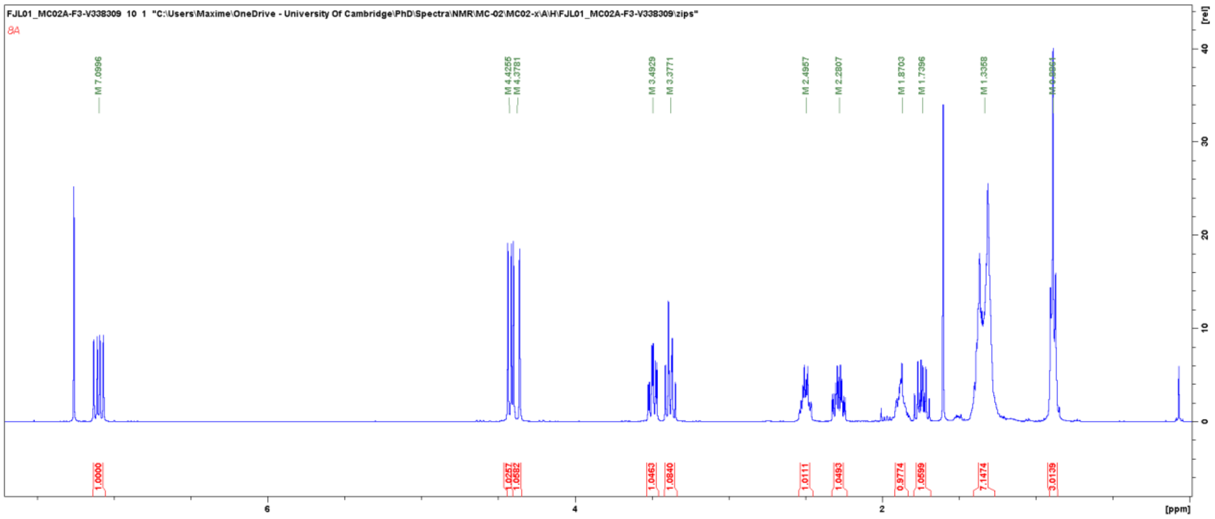

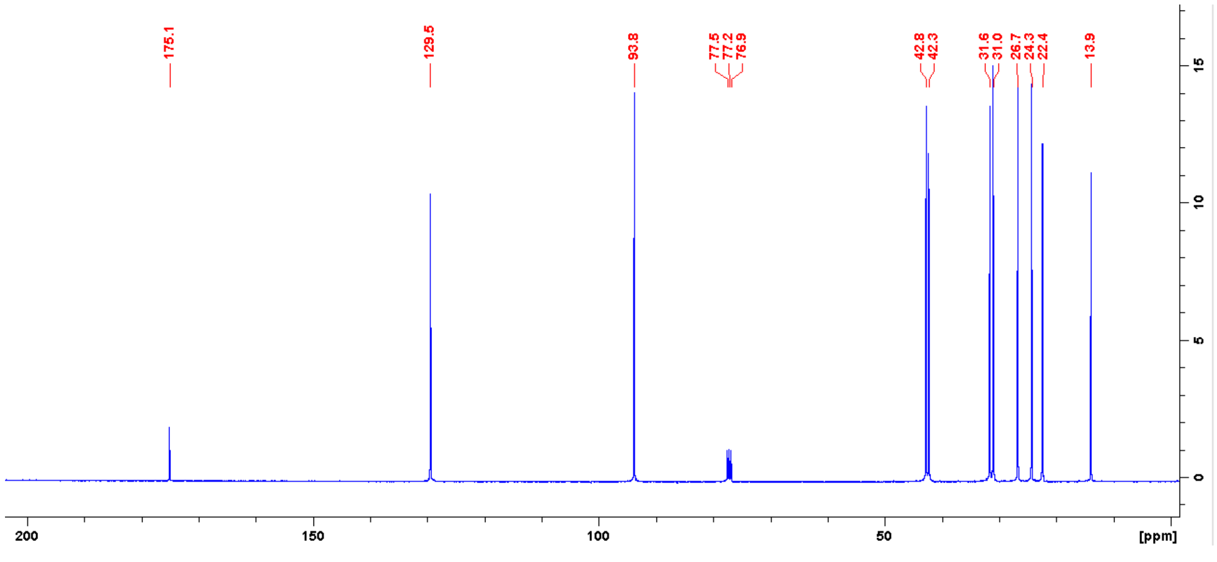

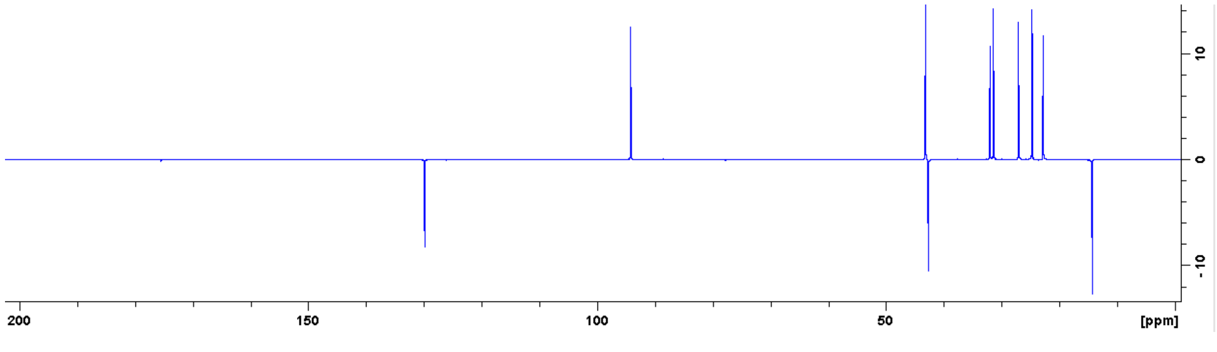

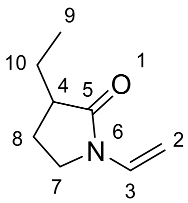
9
10
1
4
5
O
8
6
N
2
7
3

8b

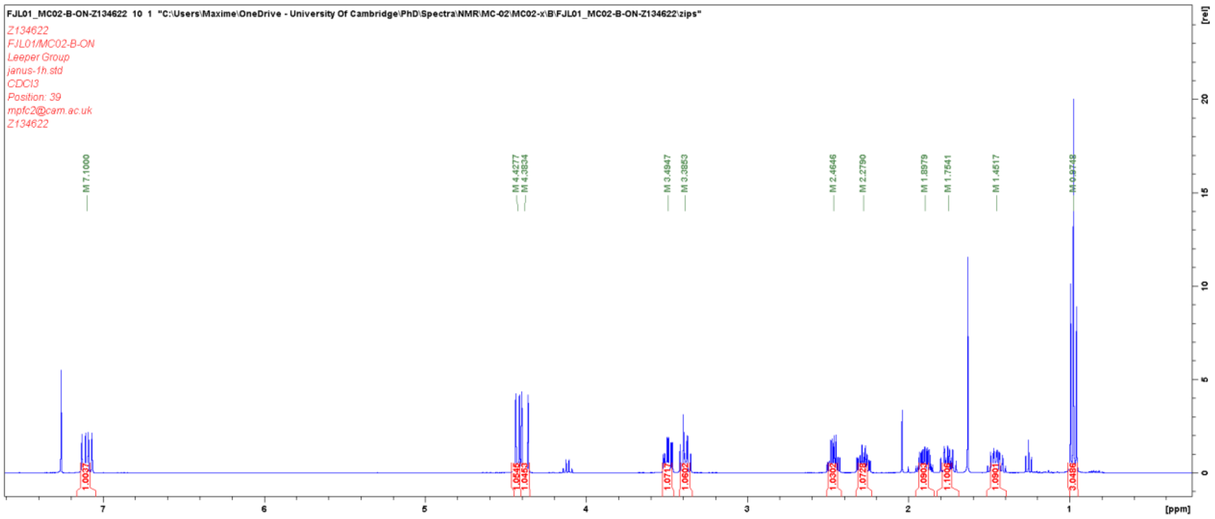
FJL01_MC02-B-ON-Z134622 10 1 "C:\Users\Maxime\OneDrive - University Of Cambridge\PhD\Spectra\NMR\MC-02\MC02-x\B\FJL01_MC02-B-ON-Z134622\zips"
Z134622
FJL01/MC02-B-ON
Leeper Group
janus-1h.std
CDCl3
Position: 39
mpfc2@cam.ac.uk
Z134622
M 7.1000
M 4.4277
M 4.3834
M 3.4947
M 3.3853
M 2.4646
M 2.2790
M 1.8979
M 1.7541
M 1.4517
M 0.9748
[rel]
[ppm]

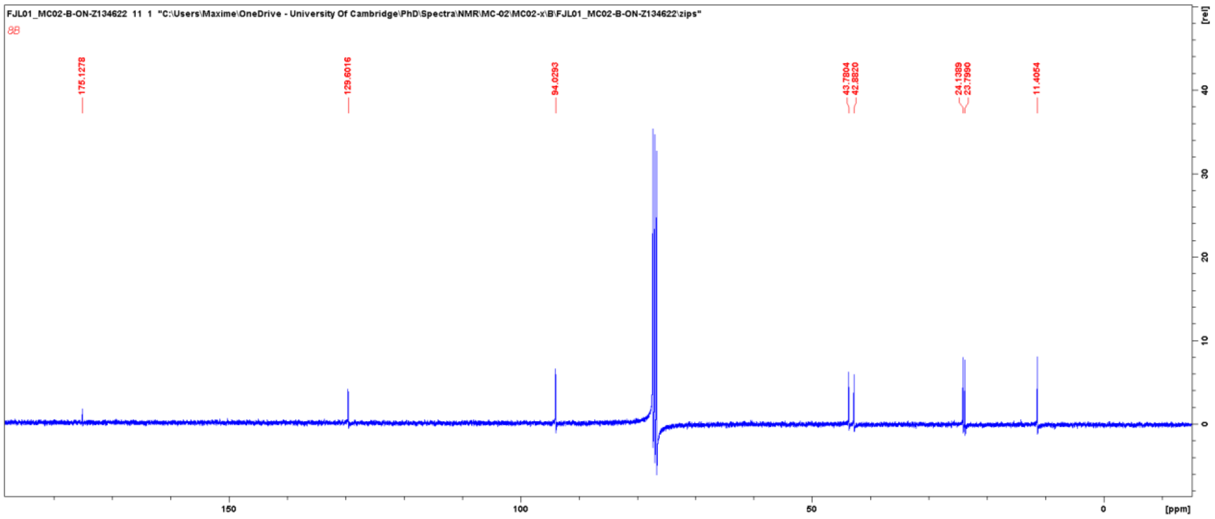
FJL01_MC02-B-ON-Z134622 11 1 "C:\Users\Maxime\OneDrive - University Of Cambridge\PhD\Spectra\NMR\MC-02\MC02-x\B\FJL01_MC02-B-ON-Z134622\zips"
8B
175.1278
129.6016
94.0293
43.7804
42.8820
24.1389
23.7990
11.4054
[rel]
[ppm]

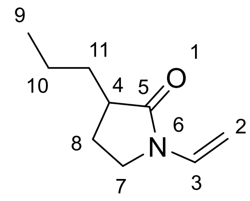
9
11
1
10
4
5
O
8
6
N
2
7
3

8c

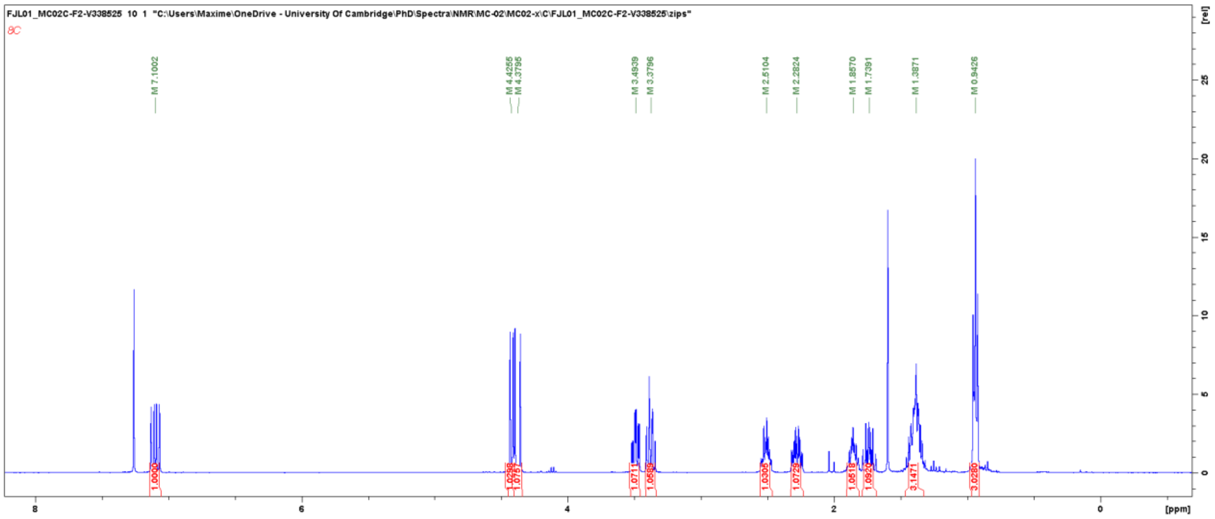
FJL01_MC02C-F2-V338525 10 1 "C:\Users\Maxime\OneDrive - University Of Cambridge\PhD\Spectra\NMR\MC-02\MC02-x\C\FJL01_MC02C-F2-V338525\zips"
8C
M 7.1002
M 4.4255
M 4.3795
M 3.4939
M 3.3796
M 2.5104
M 2.2824
M 1.8570
M 1.7391
M 1.3871
M 0.9426
[ppm]
[rel]

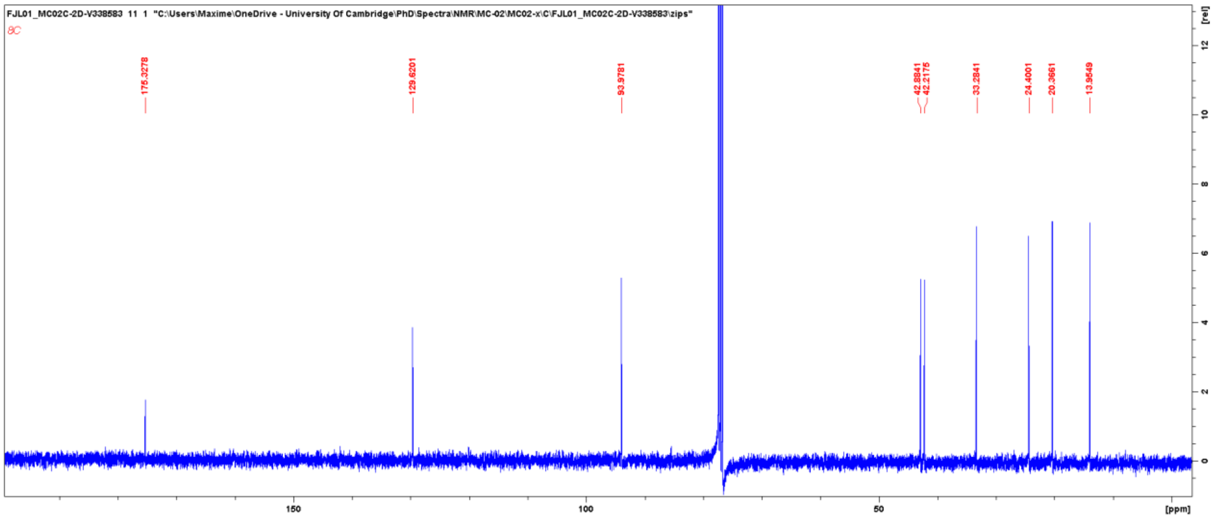
FJL01_MC02C-2D-V338583 11 1 "C:\Users\Maxime\OneDrive - University Of Cambridge\PhD\Spectra\NMR\MC-02\MC02-x\C\FJL01_MC02C-2D-V338583\zips"
8C
175.3278
129.6201
93.9781
42.8841
42.2175
33.2841
24.4001
20.3661
13.9549
[ppm]
[rel]

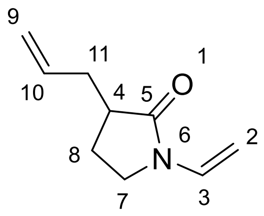
9
11
1
10
4
5
O
6
2
8
N
7
3

8d

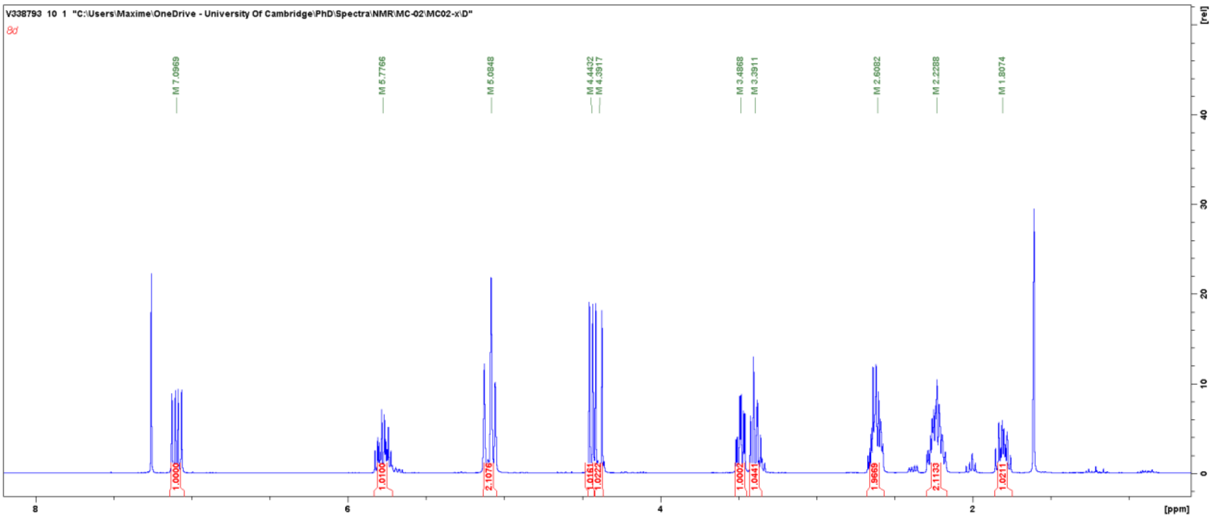
V338793 10 1 "C:\Users\Maxime\OneDrive - University Of Cambridge\PhD\Spectra\NMR\MC-02\MC02-x\D"
8d
M 7.0969
M 5.7766
M 5.0848
M 4.4432
M 4.3917
M 3.4868
M 3.3911
M 2.6082
M 2.2288
M 1.8074
1.0000
1.0100
2.1076
1.0161
1.0222
1.0002
1.0441
1.9669
2.1132
1.0211
[rel]
40
30
20
10
0
8
6
4
2
[ppm]

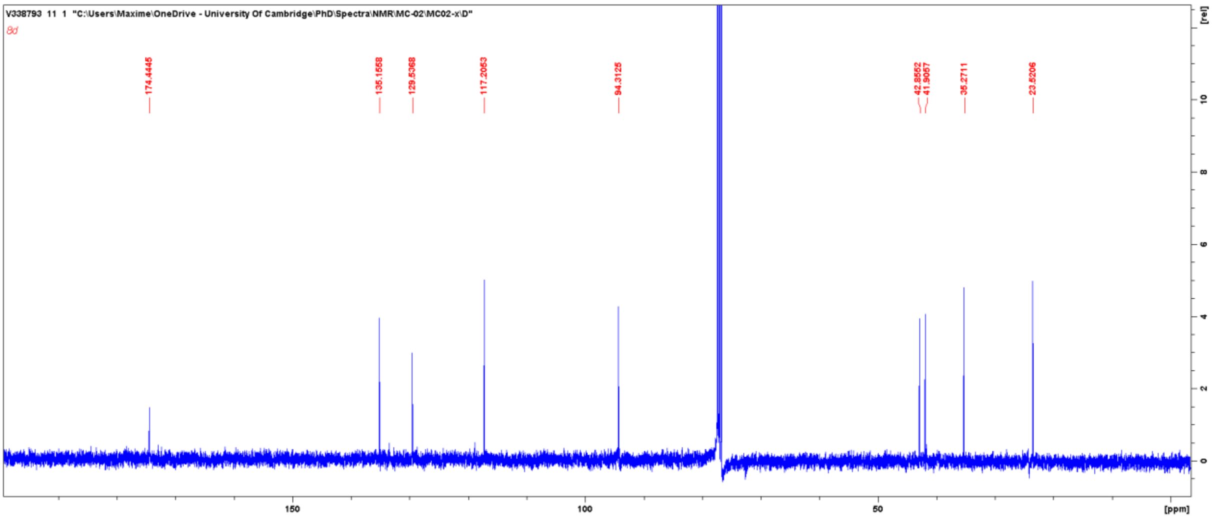
V338793 11 1 "C:\Users\Maxime\OneDrive - University Of Cambridge\PhD\Spectra\NMR\MC-02\MC02-x\D"
8d
174.4445
135.1558
129.5368
117.2063
94.3125
42.8552
41.9057
35.2711
23.5206
[rel]
10
8
6
4
2
0
150
100
50
[ppm]

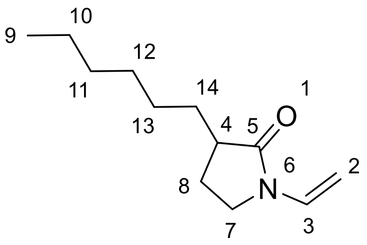
10
9
12
11
14
1
13
4
5
O
6
2
8
N
7
3

8e

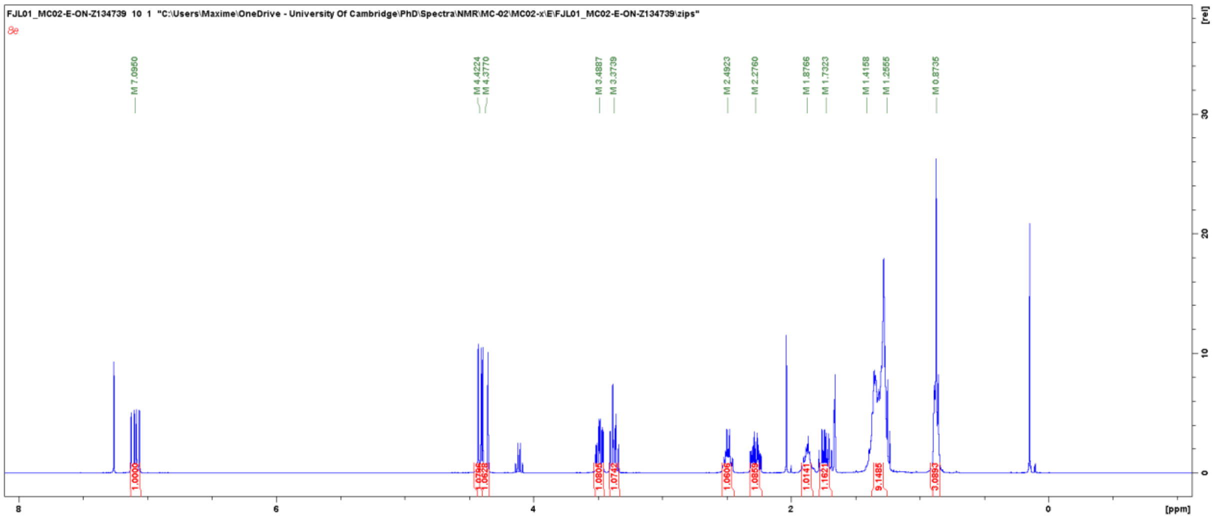
FJL01_MC02-E-ON-Z134739 10 1 "C:\Users\Maxime\OneDrive - University Of Cambridge\PhD\Spectra\NMR\MC-02\MC02-x\E\FJL01_MC02-E-ON-Z134739\zips"
8e
M 7.0950
M 4.4224
M 4.3770
M 3.4887
M 3.3739
M 2.4923
M 2.2760
M 1.8766
M 1.7323
M 1.4158
M 1.2565
M 0.8735
1.0000
1.0742
1.0606
1.0141
9.1485
3.0893
[rel]
[ppm]

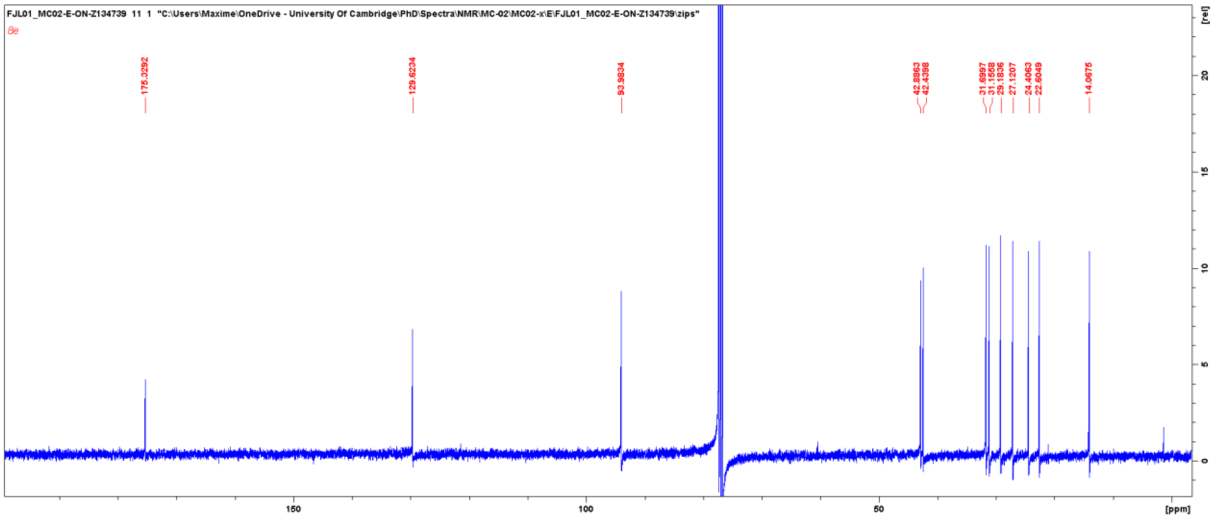
FJL01_MC02-E-ON-Z134739 11 1 "C:\Users\Maxime\OneDrive - University Of Cambridge\PhD\Spectra\NMR\MC-02\MC02-x\E\FJL01_MC02-E-ON-Z134739\zips"
8e
175.3292
129.6234
93.9834
42.8863
42.4398
31.6997
31.1558
29.1836
27.1207
24.4063
22.6049
14.0675
[rel]
[ppm]

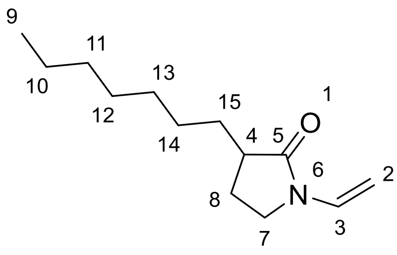
9
11
13
15
1
10
12
4
5
O
14
6
2
N
8
7
3

8f

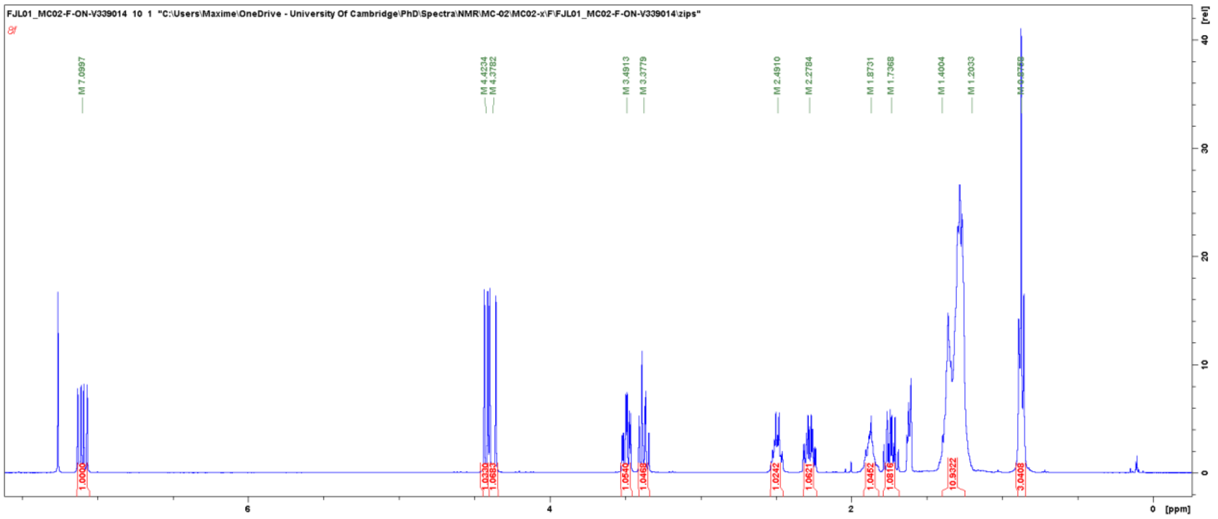
FJL01_MC02-F-ON-V339014 10 1 "C:\Users\Maxime\OneDrive - University Of Cambridge\PhD\Spectra\NMR\MC-02\MC02-x\F\FJL01_MC02-F-ON-V339014\zips"
8f
M 7.0997
M 4.4234
M 4.3782
M 3.4913
M 3.3779
M 2.4910
M 2.2784
M 1.8731
M 1.7368
M 1.4004
M 1.2033
M 0.8758
[rel]
[ppm]

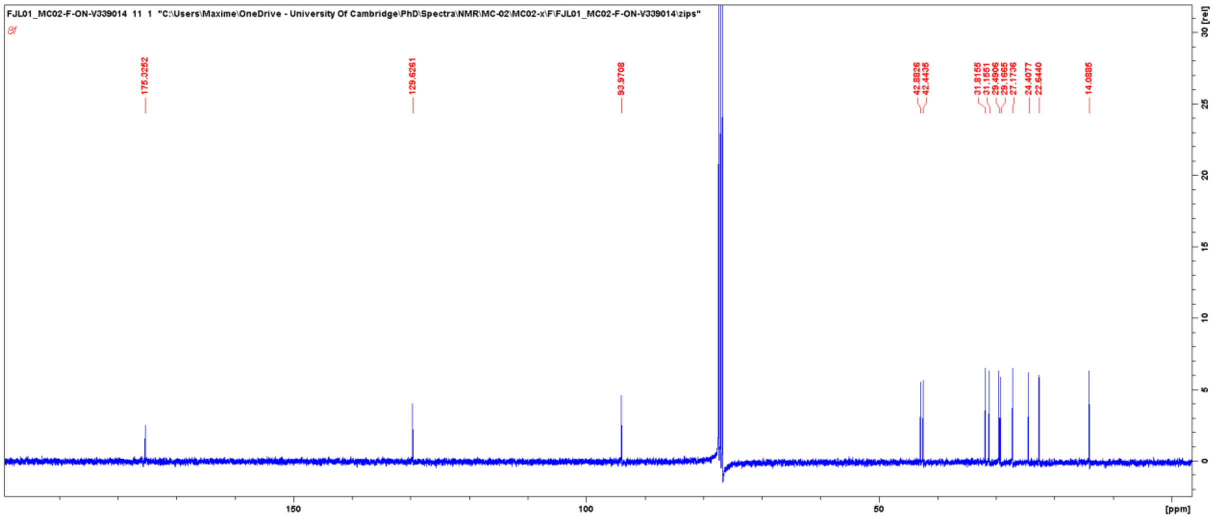
FJL01_MC02-F-ON-V339014 11 1 "C:\Users\Maxime\OneDrive - University Of Cambridge\PhD\Spectra\NMR\MC-02\MC02-x\F\FJL01_MC02-F-ON-V339014\zips"
8f
175.3252
129.6261
93.9708
42.8826
42.4435
31.8155
31.1551
29.4906
29.1665
27.1736
24.4077
22.6440
14.0885
[rel]
[ppm]

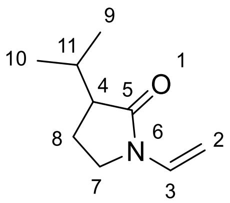

9
10
11
1
4
5
O
8
6
N
2
7
3

8g

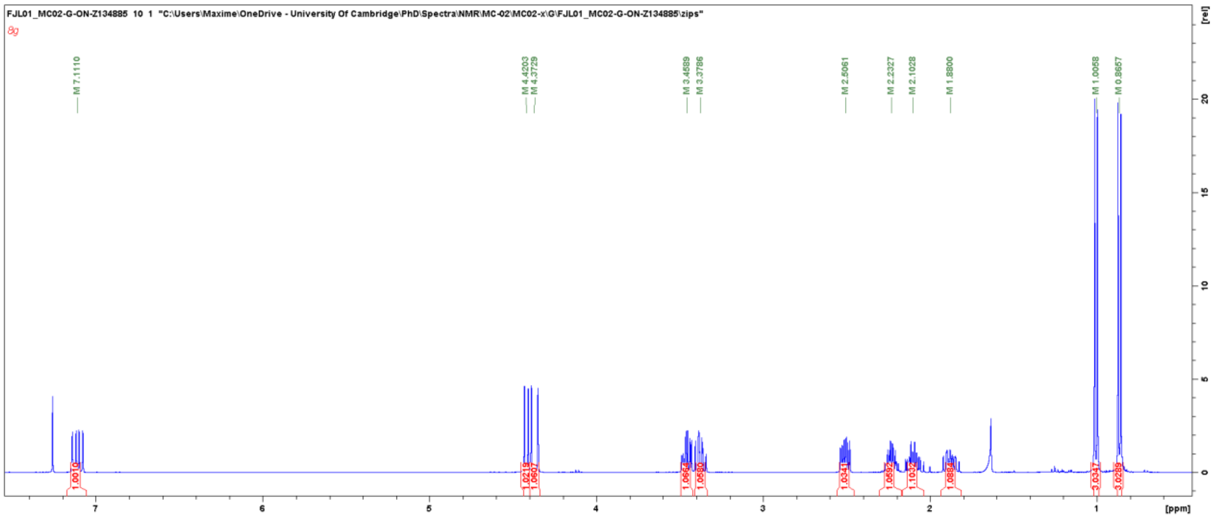

FJL01_MC02-G-ON-Z134885 10 1 "C:\Users\Maxime\OneDrive - University Of Cambridge\PhD\Spectra\NMR\MC-02\MC02-x\G\FJL01_MC02-G-ON-Z134885\zips"
8g
M 7.1110
M 4.4203
M 4.3729
M 3.4589
M 3.3786
M 2.5061
M 2.2327
M 2.1028
M 1.8800
M 1.0058
M 0.8657
[rel]
[ppm]

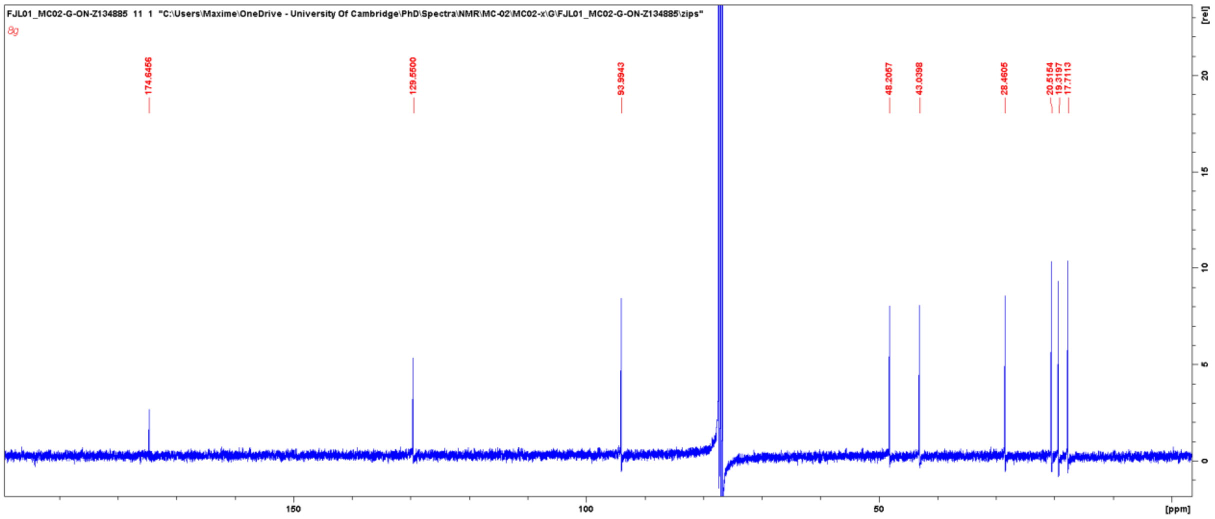

FJL01_MC02-G-ON-Z134885 11 1 "C:\Users\Maxime\OneDrive - University Of Cambridge\PhD\Spectra\NMR\MC-02\MC02-x\G\FJL01_MC02-G-ON-Z134885\zips"
8g
174.6456
129.5500
93.9943
48.2057
43.0398
28.4605
20.5164
19.3197
17.7113
[rel]
[ppm]

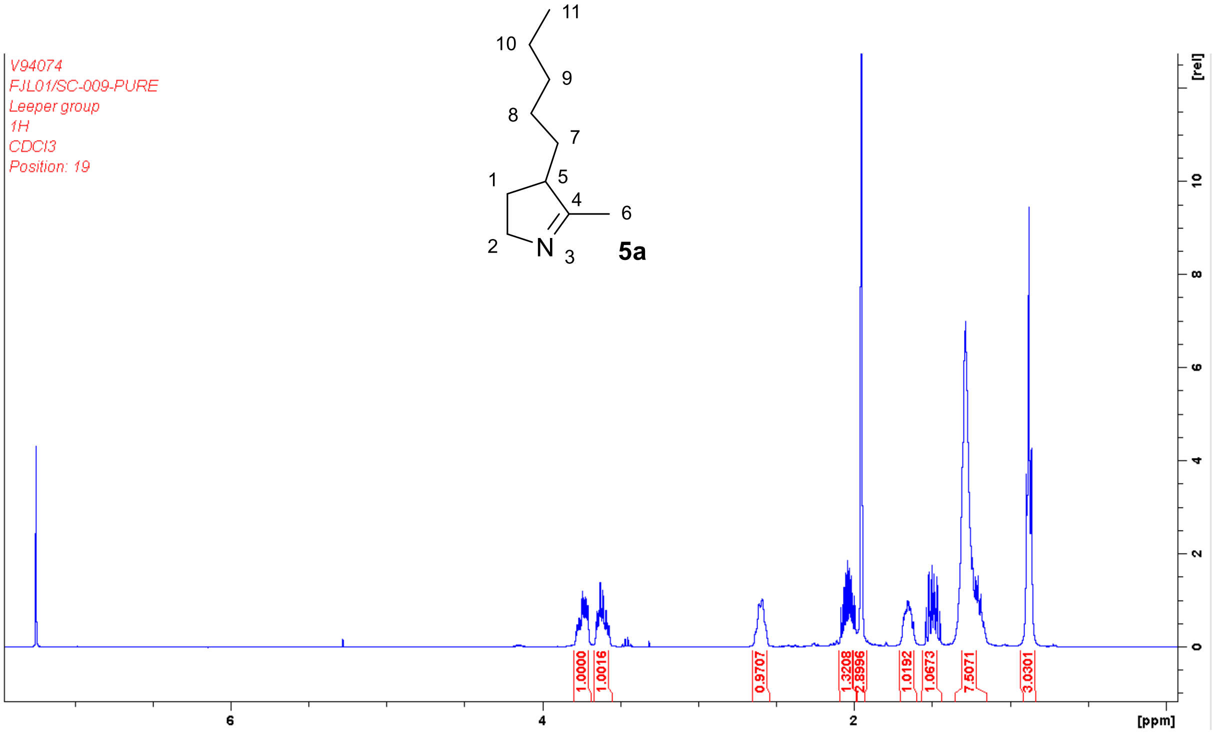

11
10
9
8
7
1
5
4
6
2
N
3
5a
V94074
FJL01/SC-009-PURE
Leeper group
1H
CDCl3
Position: 19
1.0000
1.0016
0.9707
1.3208
2.8996
1.0192
1.0673
7.5071
3.0301
6
4
2
[ppm]
[rel]

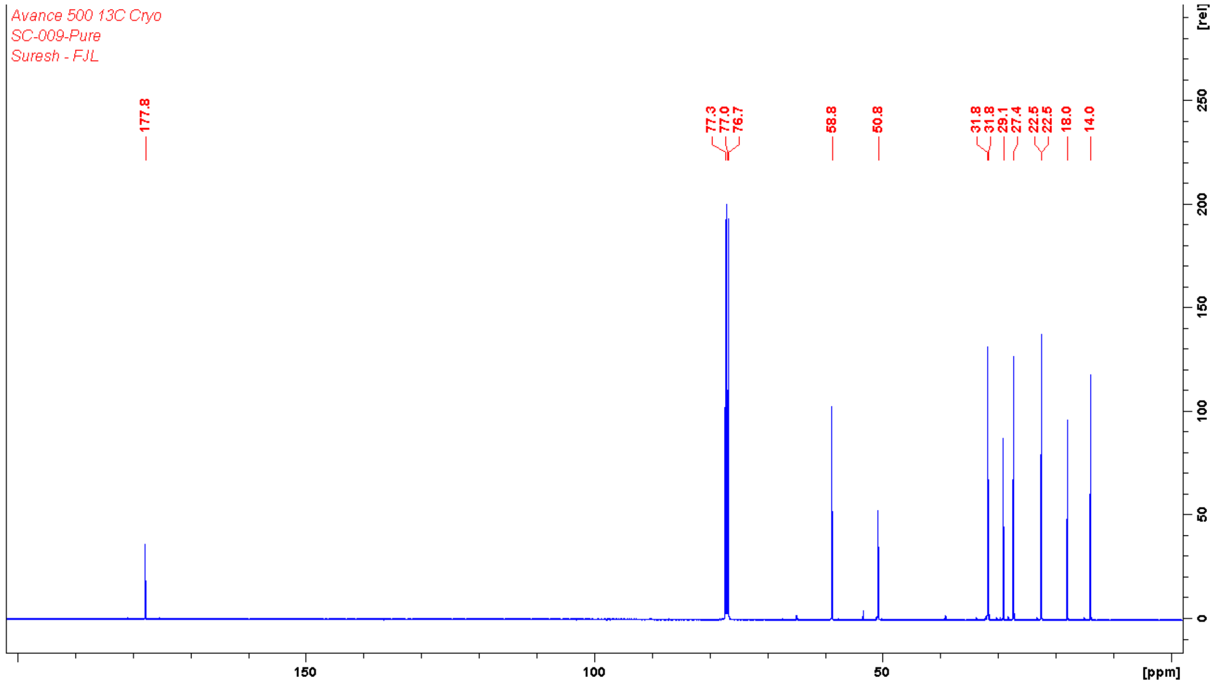

Avance 500 13C Cryo
SC-009-Pure
Suresh - FJL
177.8
77.3
77.0
76.7
58.8
50.8
31.8
31.8
29.1
27.4
22.5
22.5
18.0
14.0
150
100
50
[ppm]
[rel]

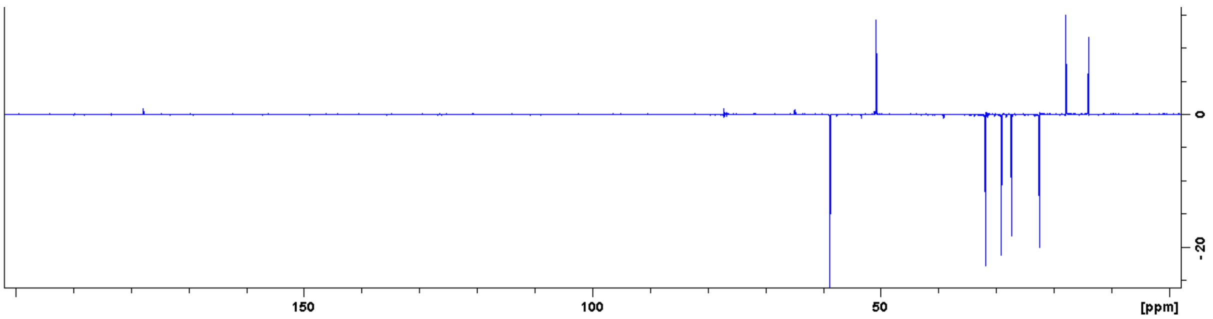

150
100
50
[ppm]

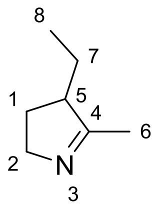

8
7
1
5
4
6
2
N
3

5b

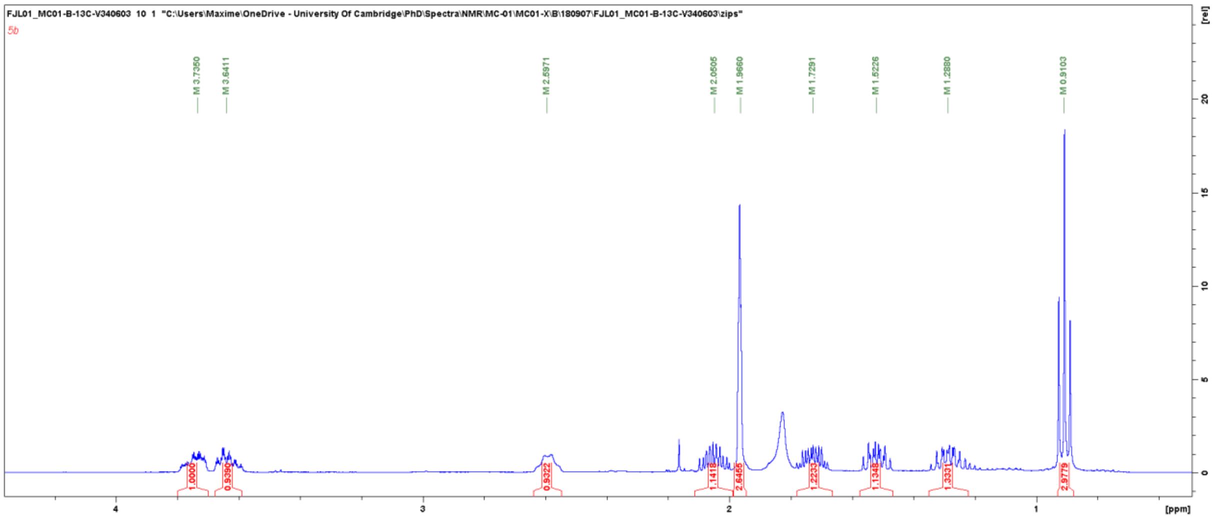

FJL01_MC01-B-13C-V340603 10 1 "C:\Users\Maxime\OneDrive - University Of Cambridge\PhD\Spectra\NMR\MC-01\MC01-X\B\180907\FJL01_MC01-B-13C-V340603\zips"
5b
M 3.7350
M 3.6411
M 2.5971
M 2.0605
M 1.9660
M 1.7291
M 1.5226
M 1.2880
M 0.9103
1.0000
0.9390
0.9322
1.1418
2.6495
2.2233
1.1348
1.3331
2.9779
[ppm]
[rel]

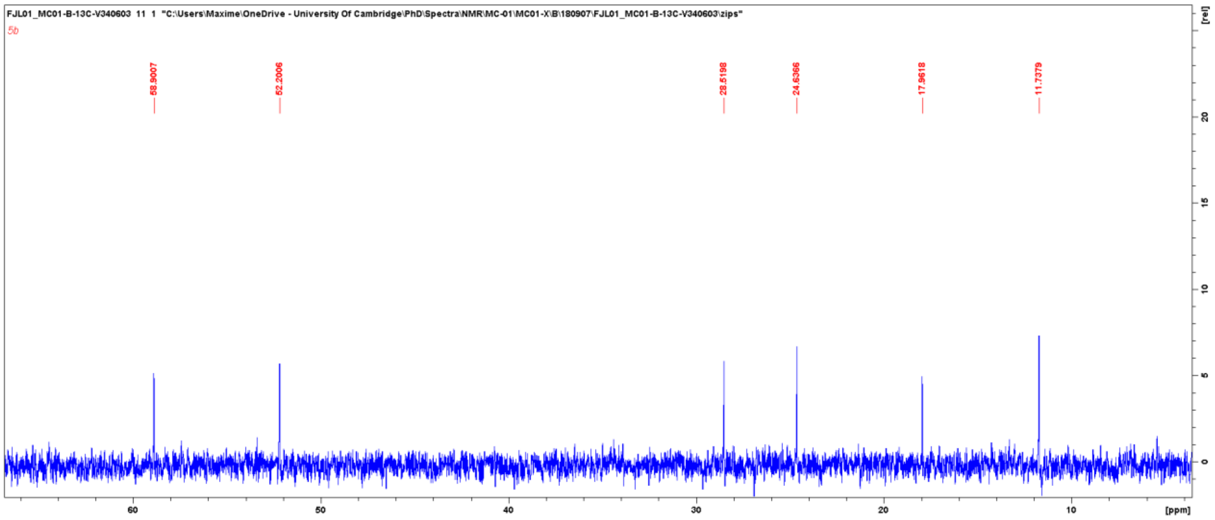

FJL01_MC01-B-13C-V340603 11 1 "C:\Users\Maxime\OneDrive - University Of Cambridge\PhD\Spectra\NMR\MC-01\MC01-X\B\180907\FJL01_MC01-B-13C-V340603\zips"
5b
58.9007
52.2006
28.5198
24.6366
17.9618
11.7379
[ppm]
[rel]

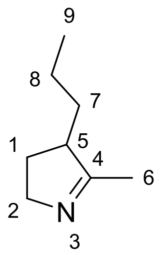
9
8
7
1
5
4
6
2
N
3

5c

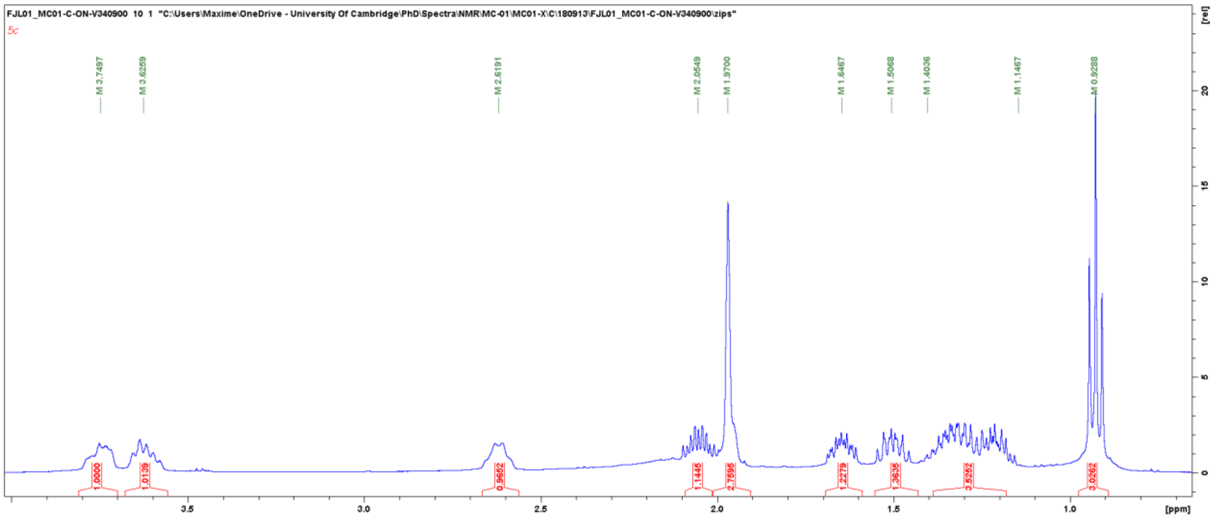
FJL01_MC01-C-ON-V340900 10 1 "C:\Users\Maxime\OneDrive - University Of Cambridge\PhD\Spectra\NMR\MC-01\MC01-X\C\180913\FJL01_MC01-C-ON-V340900\zips"
5c
M 3.7497
M 3.6259
M 2.6191
M 2.0549
M 1.9700
M 1.6467
M 1.5068
M 1.4036
M 1.1467
M 0.9288
1.0000
1.0139
0.9652
1.1445
2.7595
1.2279
1.3635
3.5252
3.0262
[ppm]
[rel]

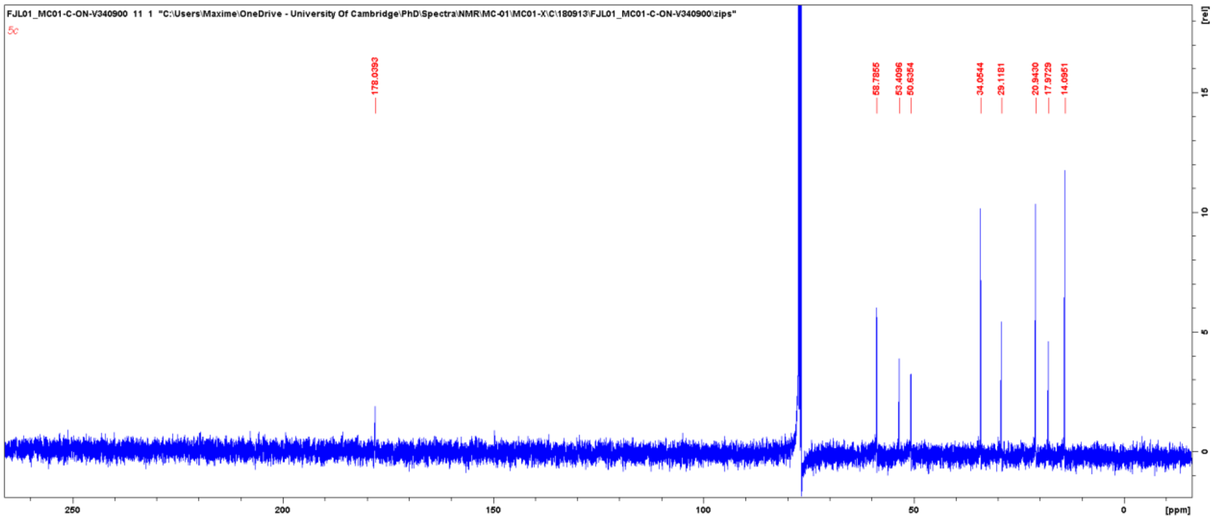
FJL01_MC01-C-ON-V340900 11 1 "C:\Users\Maxime\OneDrive - University Of Cambridge\PhD\Spectra\NMR\MC-01\MC01-X\C\180913\FJL01_MC01-C-ON-V340900\zips"
5c
178.0393
58.7855
53.4096
50.6354
34.0544
29.1181
20.9430
17.9729
14.0951
[ppm]
[rel]

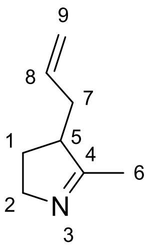

5d

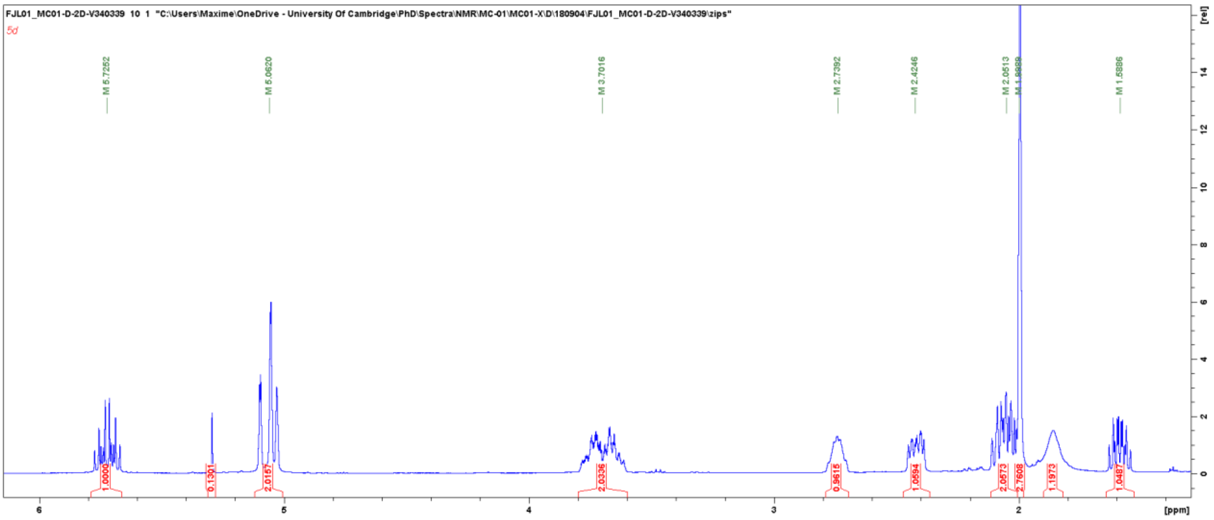

FJL01_MC01-D-2D-V340339 10 1 "C:\Users\Maxime\OneDrive - University Of Cambridge\PhD\Spectra\NMR\MC-01\MC01-X\D\180904\FJL01_MC01-D-2D-V340339\zips"
5d
M 5.7252
M 5.0620
M 3.7016
M 2.7392
M 2.4246
M 2.0613
M 1.5886
1.0000
0.1301
2.0167
2.0336
0.9615
1.0594
2.0673
2.7606
1.1973
1.0487
[ppm]
[rel]

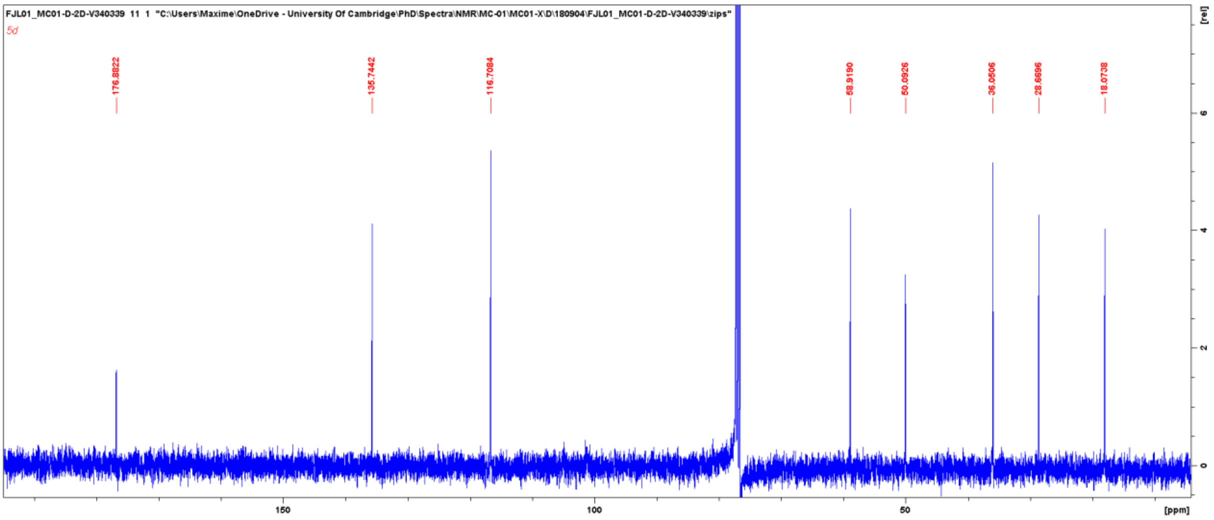

FJL01_MC01-D-2D-V340339 11 1 "C:\Users\Maxime\OneDrive - University Of Cambridge\PhD\Spectra\NMR\MC-01\MC01-X\D\180904\FJL01_MC01-D-2D-V340339\zips"
5d
176.8822
135.7442
116.7084
58.9190
50.0926
36.0506
28.6696
18.0738
[ppm]
[rel]

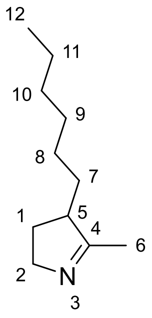
12
11
10
9
8
7
1
5
4
6
2
N
3

5e

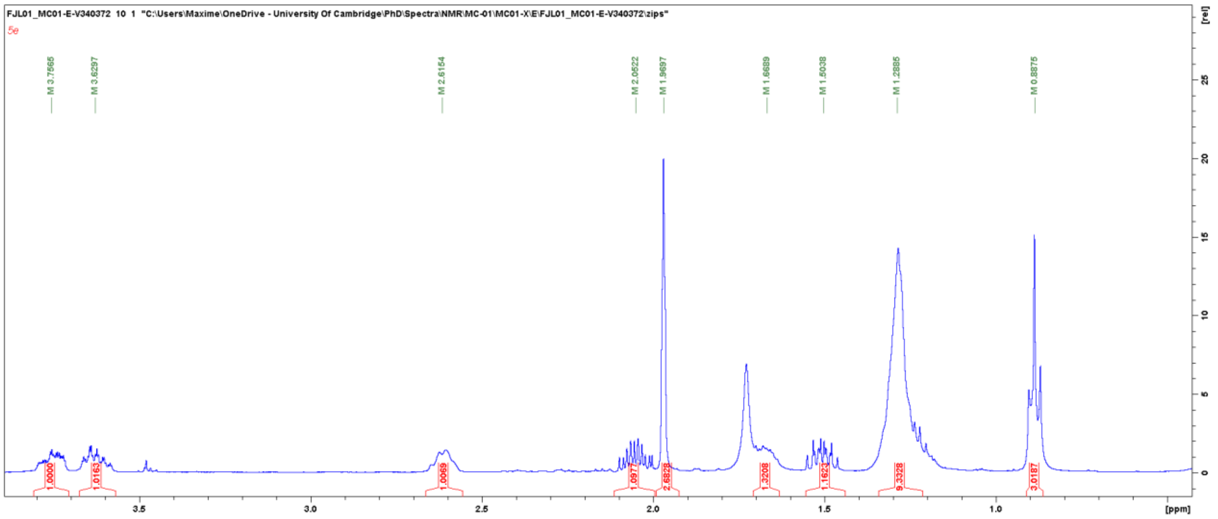
FJL01_MC01-E-V340372 10 1 "C:\Users\Maxime\OneDrive - University Of Cambridge\PhD\Spectra\NMR\MC-01\MC01-X\E\FJL01_MC01-E-V340372\zips"
5e
M 3.7565
M 3.6297
M 2.6154
M 2.0622
M 1.9697
M 1.6689
M 1.5038
M 1.2885
M 0.8875
1.0000
1.0163
1.0069
1.0977
2.6829
1.3208
1.1623
9.3328
3.0187
[ppm]
[rel]

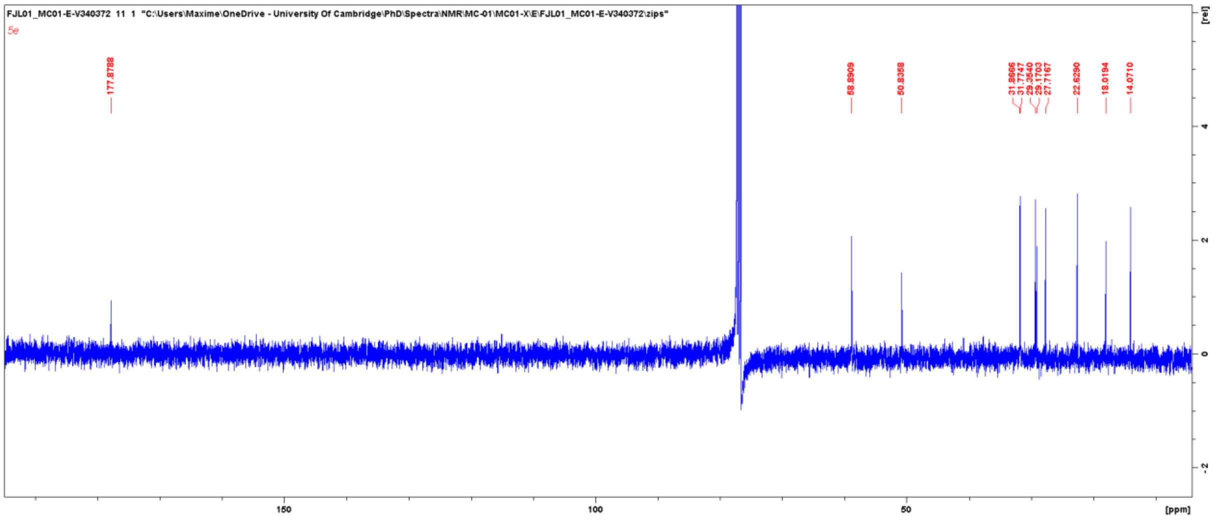
FJL01_MC01-E-V340372 11 1 "C:\Users\Maxime\OneDrive - University Of Cambridge\PhD\Spectra\NMR\MC-01\MC01-X\E\FJL01_MC01-E-V340372\zips"
5e
177.8788
58.8909
50.8358
31.8966
31.7747
29.3540
29.1703
27.7167
22.6290
18.0194
14.0710
[ppm]
[rel]

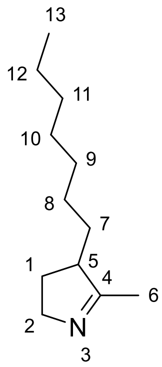
13
12
11
10
9
8
7
1
5
4
6
2
N
3

5f

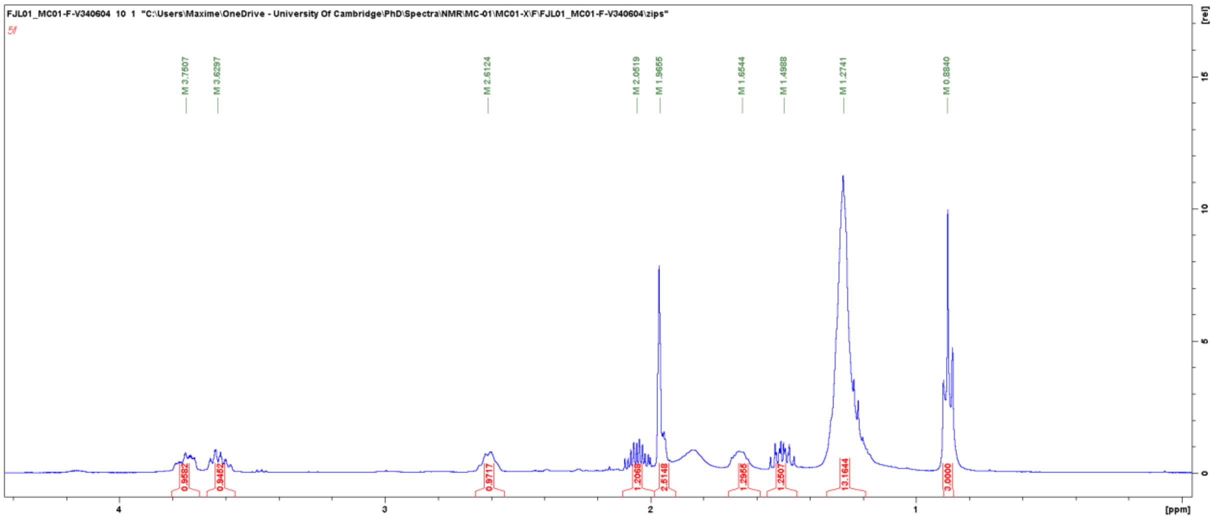
FJL01_MC01-F-V340604 10 1 "C:\Users\Maxime\OneDrive - University Of Cambridge\PhD\Spectra\NMR\MC-01\MC01-X\F\FJL01_MC01-F-V340604\zips"
5f
M 3.7607
M 3.6297
M 2.6124
M 2.0519
M 1.9655
M 1.6544
M 1.4988
M 1.2741
M 0.8840
0.9452
0.9717
2.5148
1.2565
1.2507
13.1644
3.0000
[ppm]
[rel]

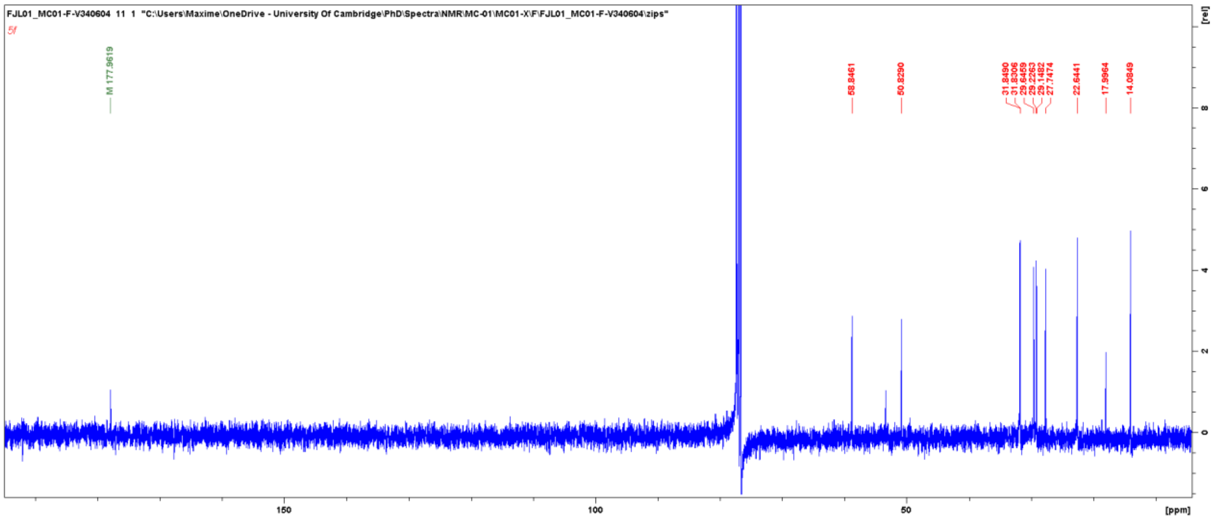
FJL01_MC01-F-V340604 11 1 "C:\Users\Maxime\OneDrive - University Of Cambridge\PhD\Spectra\NMR\MC-01\MC01-X\F\FJL01_MC01-F-V340604\zips"
5f
M 177.9619
58.8461
50.8290
31.8490
31.8306
29.6459
29.2263
29.1482
27.7474
22.6441
17.9964
14.0849
150
100
50
[ppm]
[rel]

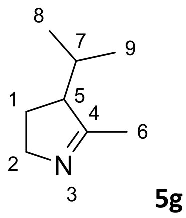
8
7
9
1
5
4
6
2
N
3

5g

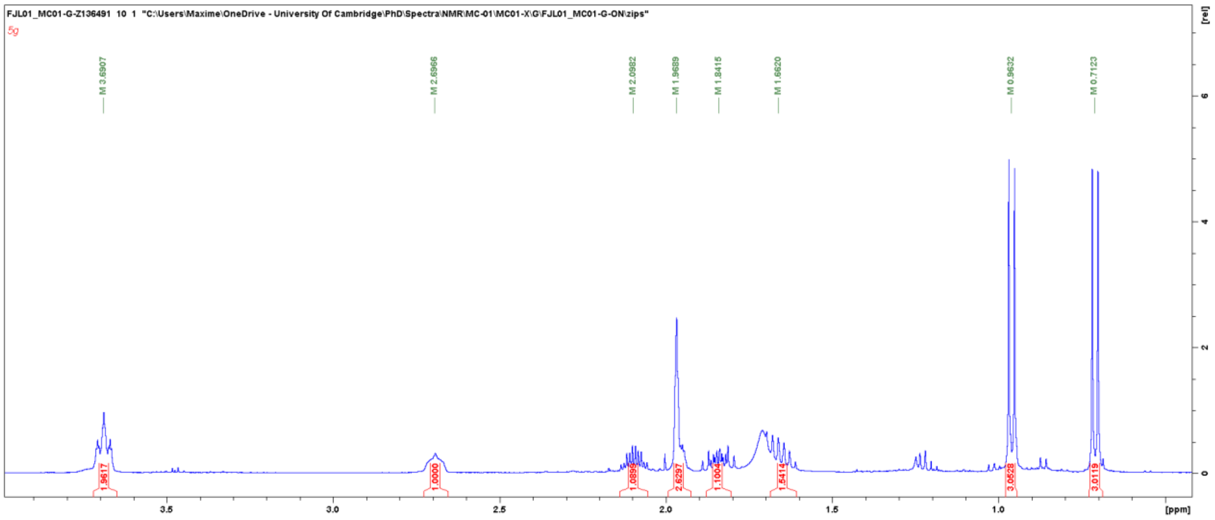
FJL01_MC01-G-Z136491 10 1 "C:\Users\Maxime\OneDrive - University Of Cambridge\PhD\Spectra\NMR\MC-01\MC01-X\G\FJL01_MC01-G-ON\zips"
5g
M 3.6907
M 2.6966
M 2.0982
M 1.9689
M 1.8415
M 1.6620
M 0.9632
M 0.7123
1.9617
1.0000
1.0899
2.6297
1.1004
1.5414
3.0528
3.0119
[rel]
[ppm]

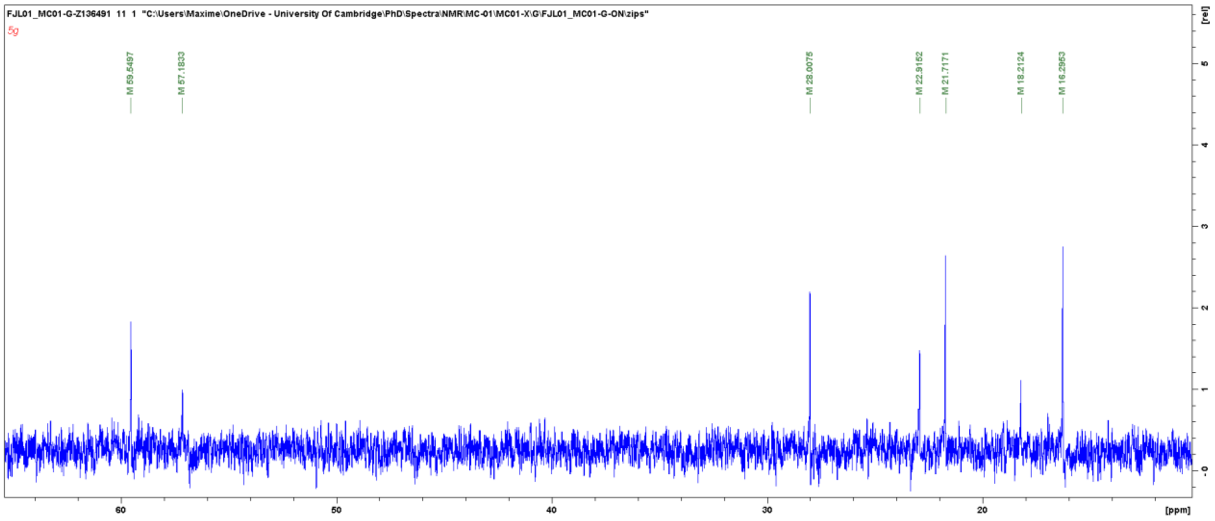
FJL01_MC01-G-Z136491 11 1 "C:\Users\Maxime\OneDrive - University Of Cambridge\PhD\Spectra\NMR\MC-01\MC01-X\G\FJL01_MC01-G-ON\zips"
5g
M 59.5497
M 57.1833
M 28.0076
M 22.9152
M 21.7171
M 18.2124
M 16.2953
[rel]
[ppm]

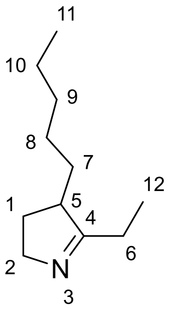

11
10
9
8
7
12
1
5
4
6
2
N
3

5h

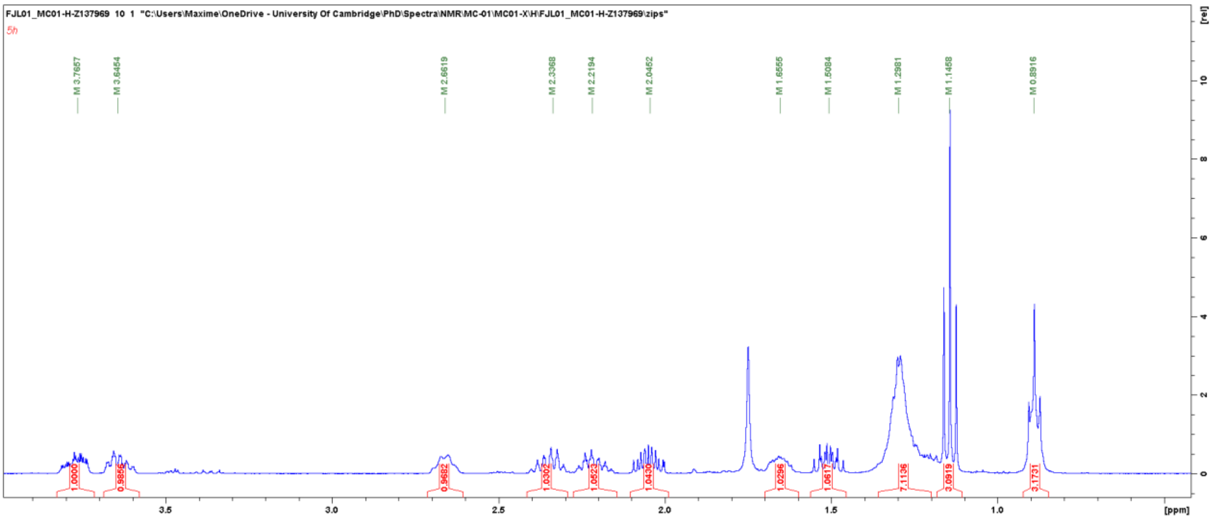

FJL01_MC01-H-Z137969 10 1 "C:\Users\Maxime\OneDrive - University Of Cambridge\PhD\Spectra\NMR\MC-01\MC01-X\H\FJL01_MC01-H-Z137969\zips"
5h
M 3.7657
M 3.6454
M 2.6619
M 2.3368
M 2.2194
M 2.0452
M 1.6555
M 1.5084
M 1.2981
M 1.1458
M 0.8916
1.0000
0.9855
0.9682
1.0302
1.0532
1.0430
1.0296
1.0617
7.1136
3.0919
3.1731
[ppm]
[rel]

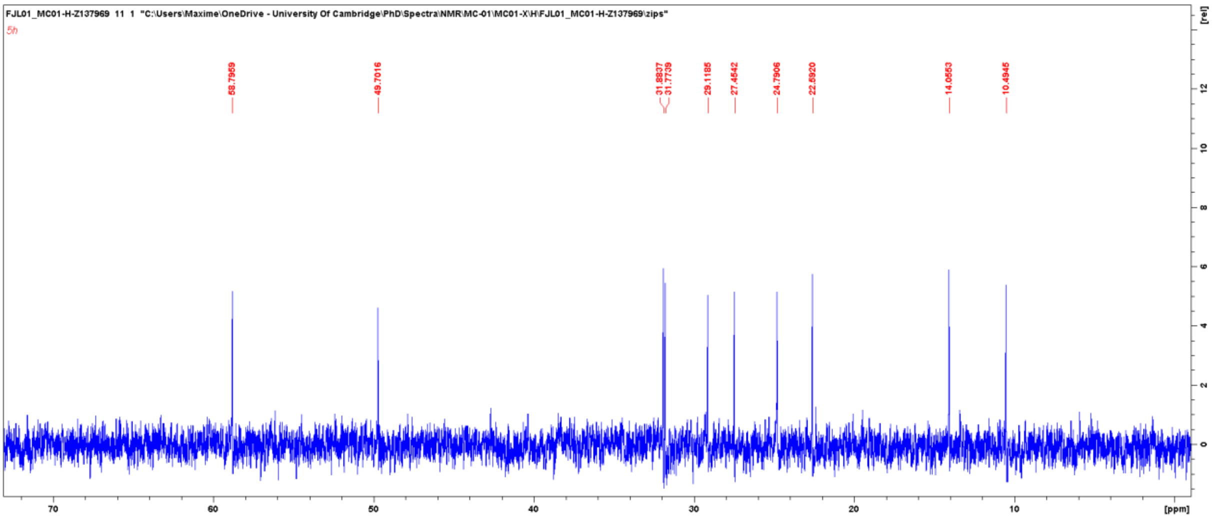

FJL01_MC01-H-Z137969 11 1 "C:\Users\Maxime\OneDrive - University Of Cambridge\PhD\Spectra\NMR\MC-01\MC01-X\H\FJL01_MC01-H-Z137969\zips"
5h
58.7959
49.7016
31.8837
31.7739
29.1185
27.4542
24.7906
22.5920
14.0553
10.4945
[ppm]
[rel]

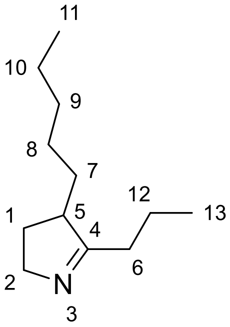
11
10
9
8
7
12
13
1
5
4
6
2
N
3

5i

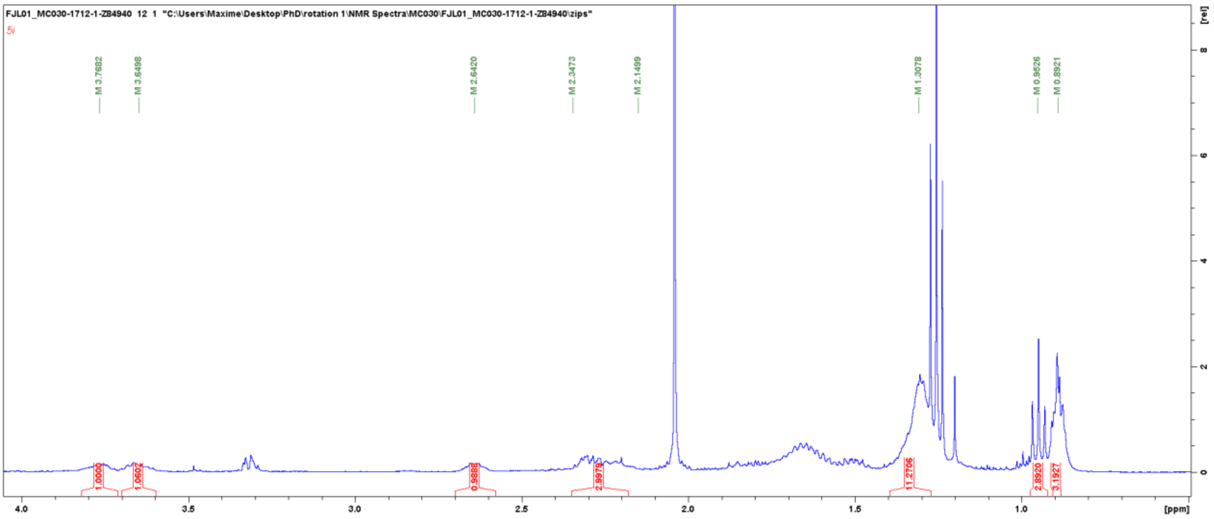
FJL01_MC030-1712-1-Z84940 12 1 "C:\Users\Maxime\Desktop\PhD\rotation 1\NMR Spectra\MC030\FJL01_MC030-1712-1-Z84940\zips"
5i
M 3.7682
M 3.6498
M 2.6420
M 2.3473
M 2.1499
M 1.3078
M 0.9526
M 0.8921
1.0000
1.0602
0.9886
2.9979
11.2706
2.8920
3.1927
[rel]
[ppm]

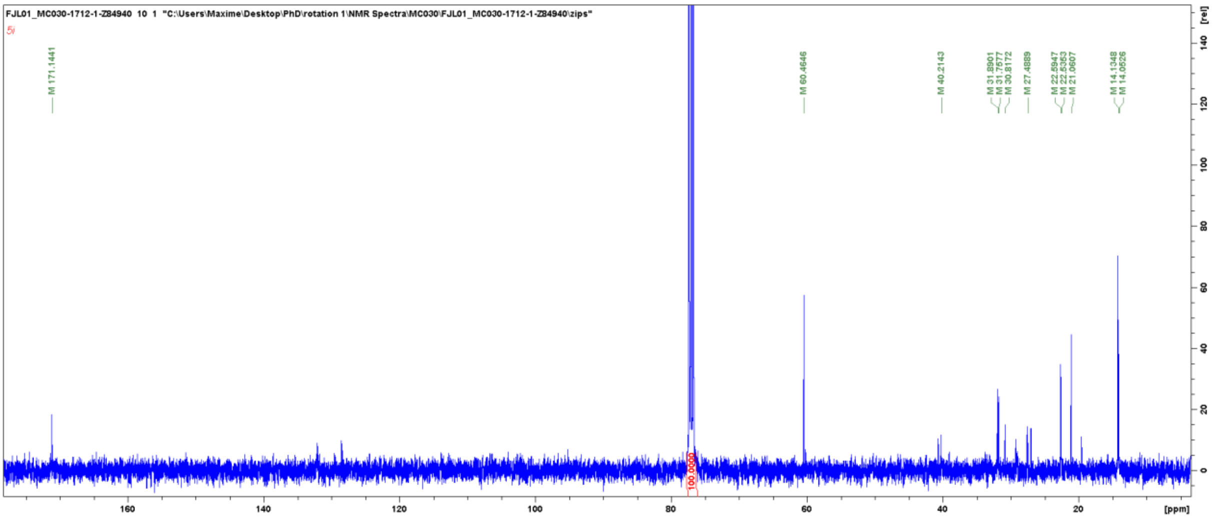
FJL01_MC030-1712-1-Z84940 10 1 "C:\Users\Maxime\Desktop\PhD\rotation 1\NMR Spectra\MC030\FJL01_MC030-1712-1-Z84940\zips"
5i
M 171.1441
M 60.4646
M 40.2143
M 31.8901
M 31.7577
M 30.8172
M 27.4889
M 22.5947
M 22.5353
M 21.0607
M 14.1348
M 14.0526
100.0000
[rel]
[ppm]

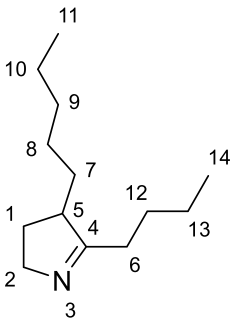
11
10
9
8
7
14
12
1
5
4
13
2
N
6
3

5j

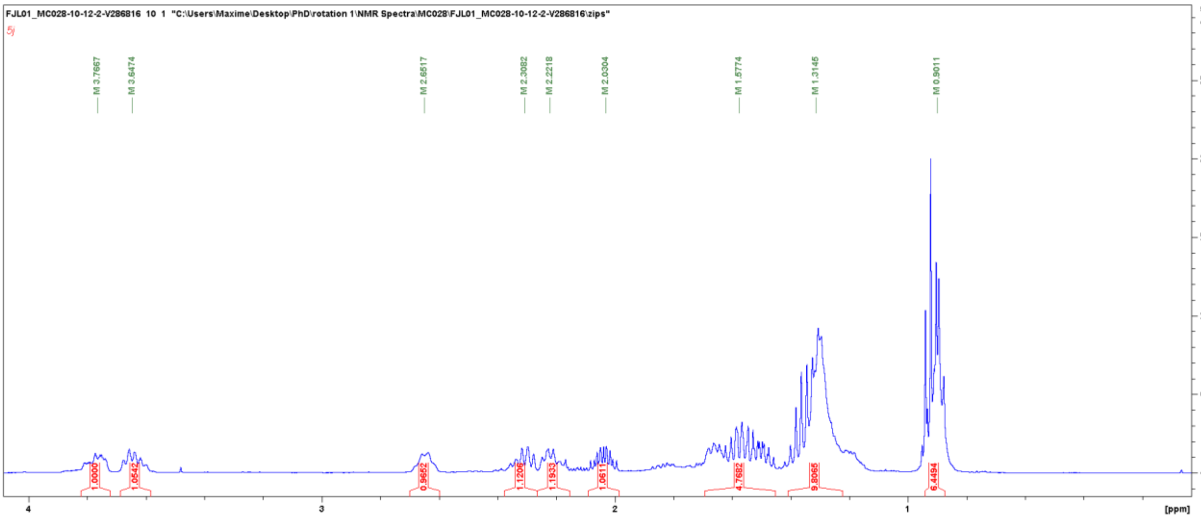
FJL01_MC028-10-12-2-V286816 10 1 "C:\Users\Maxime\Desktop\PhD\rotation 1\NMR Spectra\MC028\FJL01_MC028-10-12-2-V286816\zips"
5j
M 3.7667
M 3.6474
M 2.6517
M 2.3082
M 2.2218
M 2.0304
M 1.5774
M 1.3145
M 0.9011
1.0000
1.0542
0.9652
1.1206
1.1933
1.0611
4.7682
9.8066
6.4494
[ppm]
[rel]

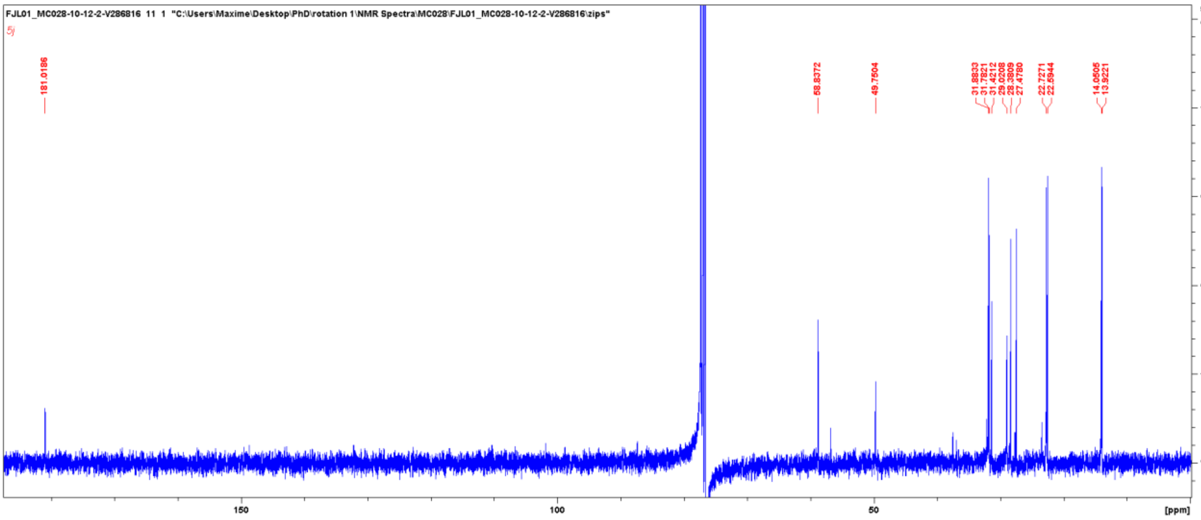
FJL01_MC028-10-12-2-V286816 11 1 "C:\Users\Maxime\Desktop\PhD\rotation 1\NMR Spectra\MC028\FJL01_MC028-10-12-2-V286816\zips"
5j
181.0186
58.8372
49.7504
31.8833
31.7821
31.4212
29.0208
28.3809
27.4780
22.7271
22.5944
14.0505
13.9221
150
100
50
[ppm]
[rel]

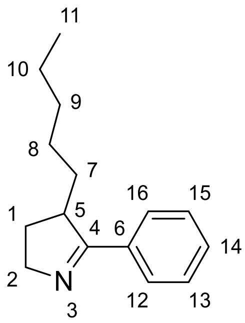
11
10
9
8
7
16
15
1
5
4
6
14
2
N
12
13
3

5k

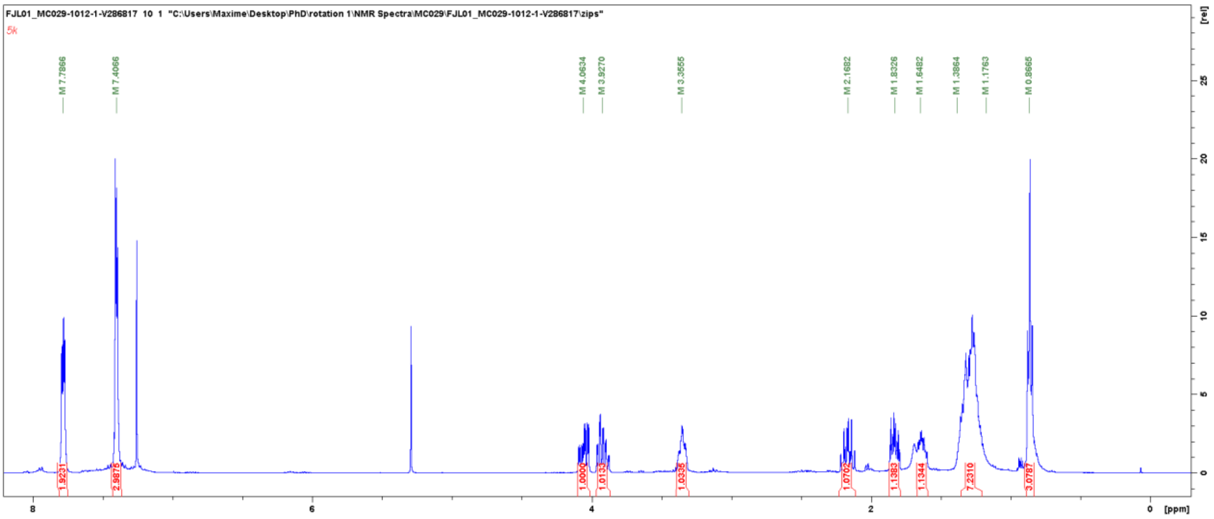
FJL01_MC029-1012-1-V286817 10 1 "C:\Users\Maxime\Desktop\PhD\rotation 1\NMR Spectra\MC029\FJL01_MC029-1012-1-V286817\zips"
5k
M 7.7966
M 7.4066
M 4.0634
M 3.9270
M 3.3555
M 2.1682
M 1.8326
M 1.6482
M 1.3864
M 1.1763
M 0.8665
1.9231
2.9875
1.0000
1.0133
1.0335
1.0702
1.1383
1.1344
7.2310
3.0787
[ppm]
[rel]

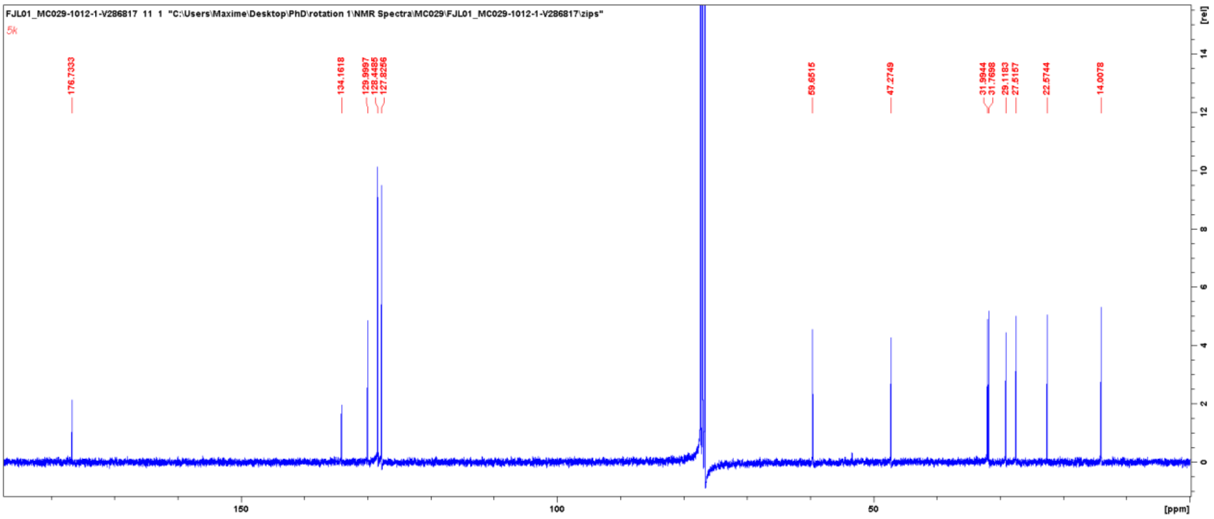
FJL01_MC029-1012-1-V286817 11 1 "C:\Users\Maxime\Desktop\PhD\rotation 1\NMR Spectra\MC029\FJL01_MC029-1012-1-V286817\zips"
5k
176.7333
134.1618
129.9997
128.4485
127.8256
59.6515
47.2749
31.9944
31.7698
29.1182
27.5157
22.5744
14.0078
[ppm]
[rel]

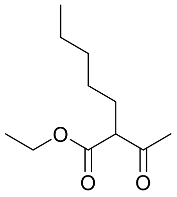
O
O
O

10

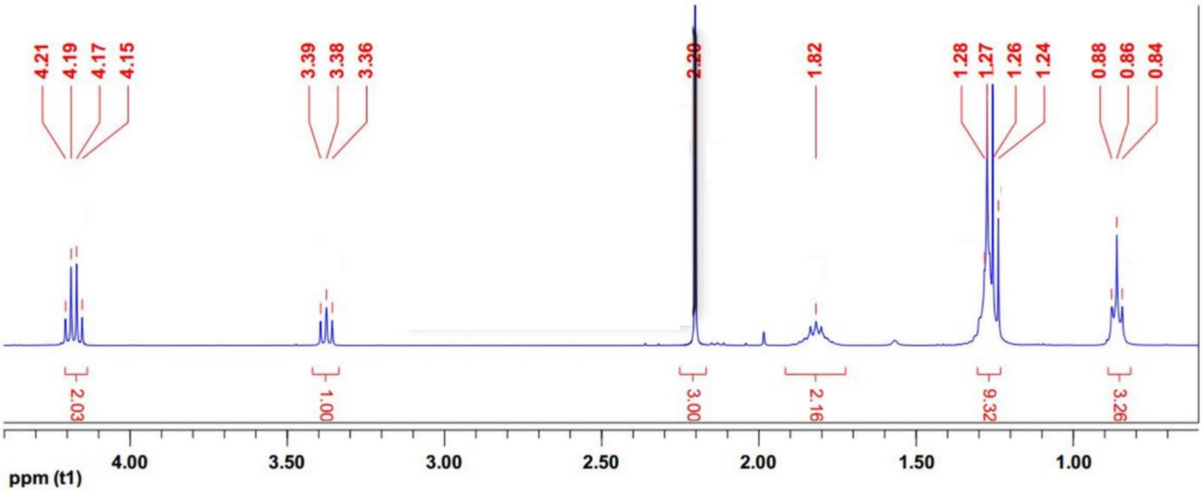
4.21
4.19
4.17
4.15
3.39
3.38
3.36
2.20
1.82
1.28
1.27
1.26
1.24
0.88
0.86
0.84
2.03
1.00
3.00
2.16
9.32
3.26
4.00
3.50
3.00
2.50
2.00
1.50
1.00
ppm (t1)

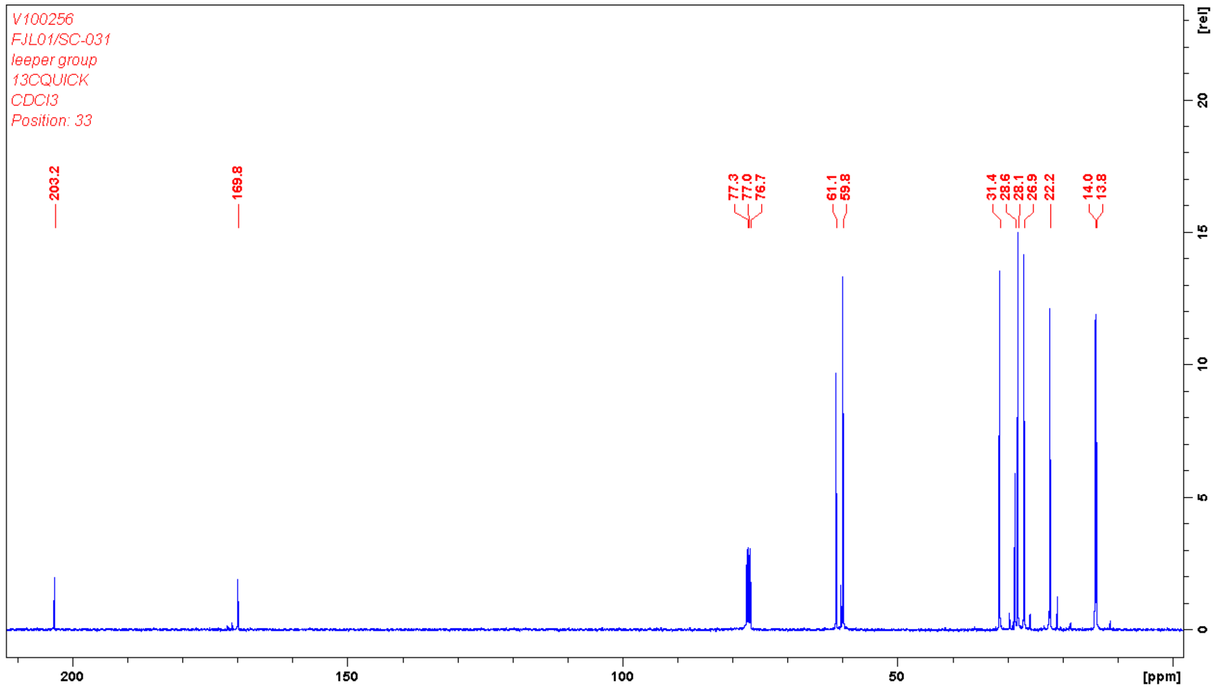
V100256
FJL01/SC-031
leeper group
13CQUICK
CDCl3
Position: 33
203.2
169.8
77.3
77.0
76.7
61.1
59.8
31.4
28.6
28.1
26.9
22.2
14.0
13.8
200
150
100
50
[ppm]
[rel]
20
15
10
5
0

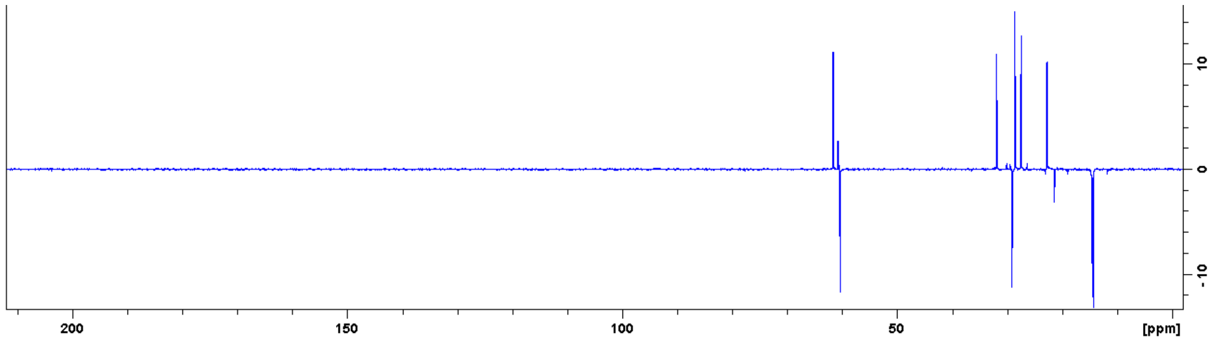
200
150
100
50
[ppm]
10
0
-10

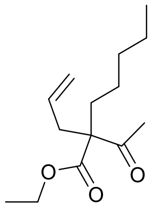

11

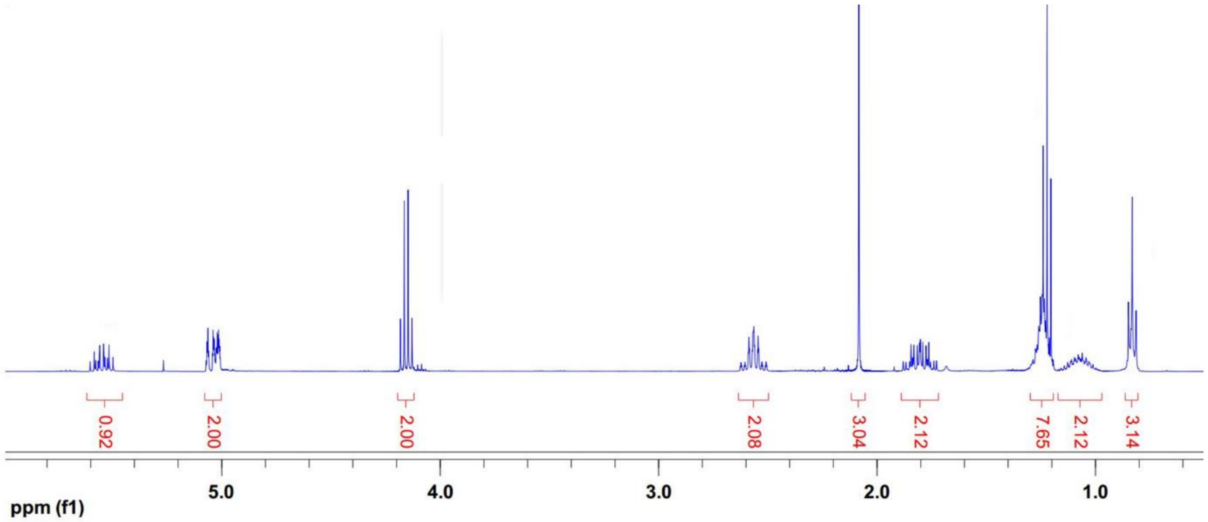
0.92
2.00
2.00
2.08
3.04
2.12
7.65
2.12
3.14
5.0
4.0
3.0
2.0
1.0
ppm (f1)

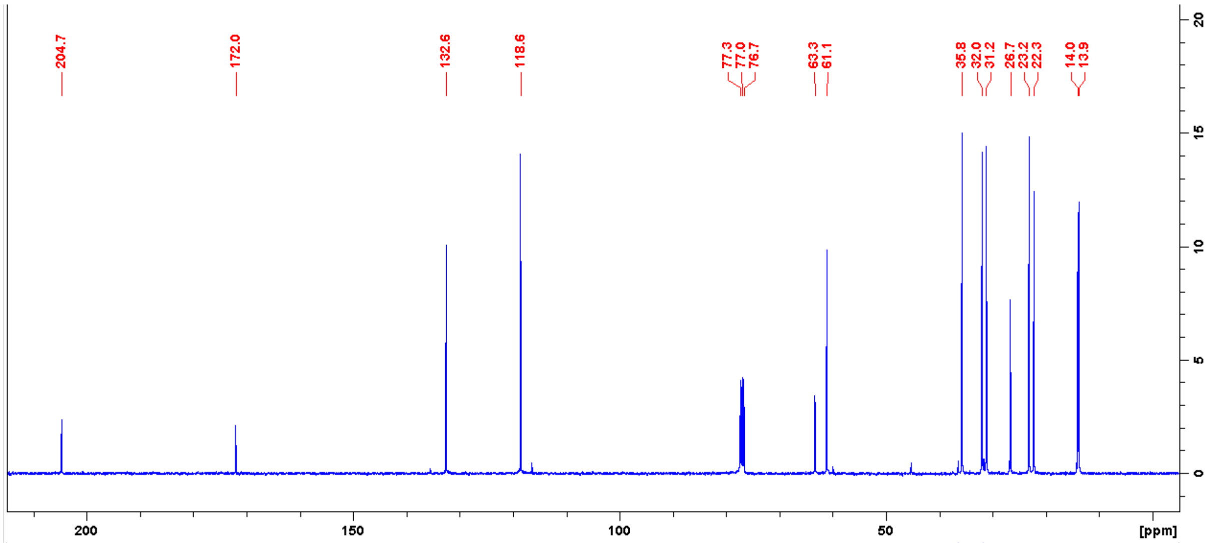
204.7
172.0
132.6
118.6
77.3
77.0
76.7
63.3
61.1
35.8
32.0
31.2
26.7
23.2
22.3
14.0
13.9
20
15
10
5
0
200
150
100
50
[ppm]

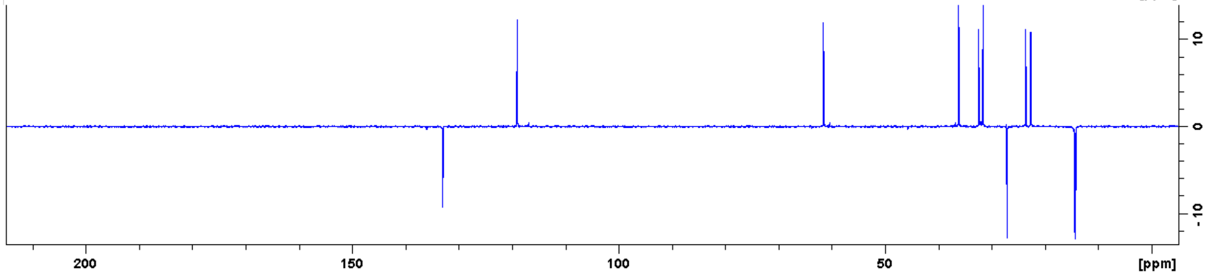
10
0
-10
200
150
100
50
[ppm]

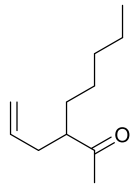

12

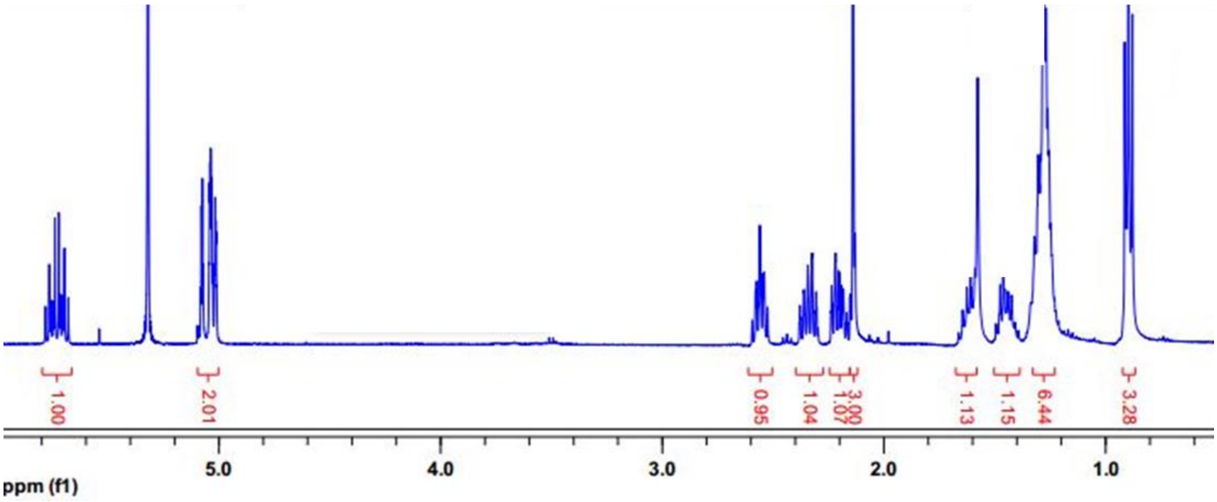

1.00
2.01
0.95
1.04
1.07
3.00
1.13
1.15
6.44
3.28
5.0
4.0
3.0
2.0
1.0
ppm (f1)

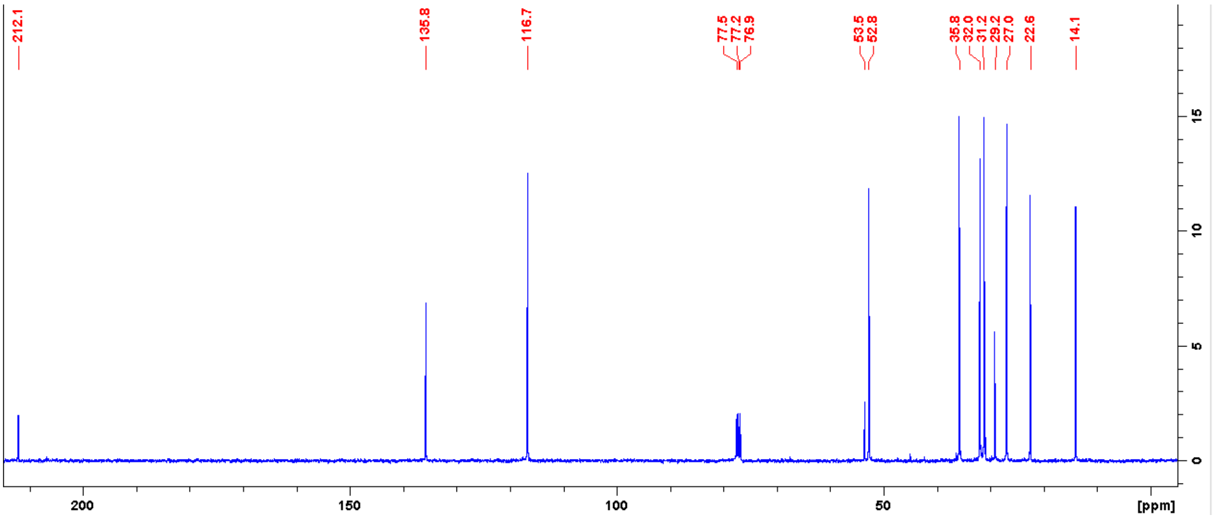

212.1
135.8
116.7
77.5
77.2
76.9
53.5
52.8
35.8
32.0
31.2
29.2
27.0
22.6
14.1
200
150
100
50
[ppm]
15
10
5
0

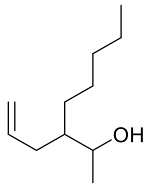

13

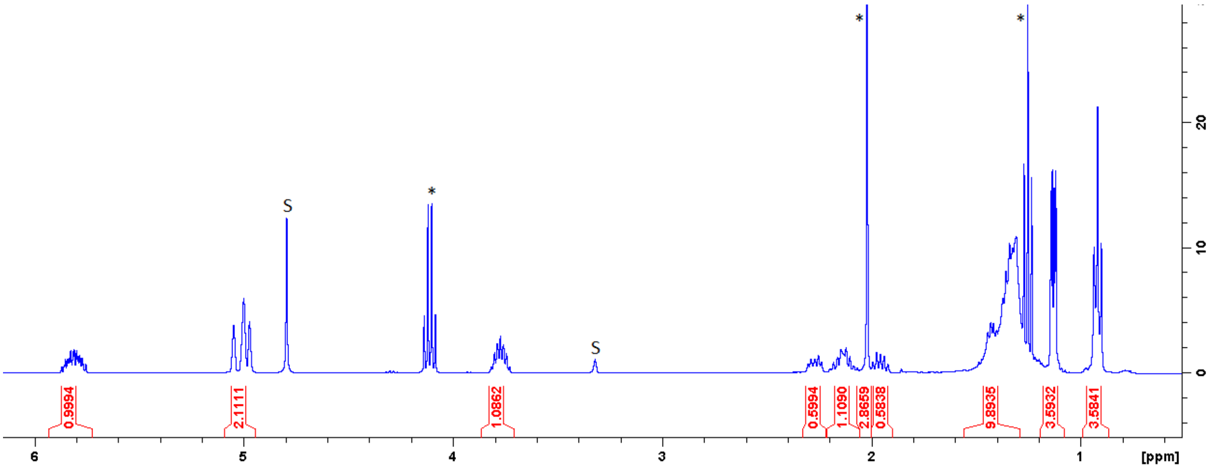

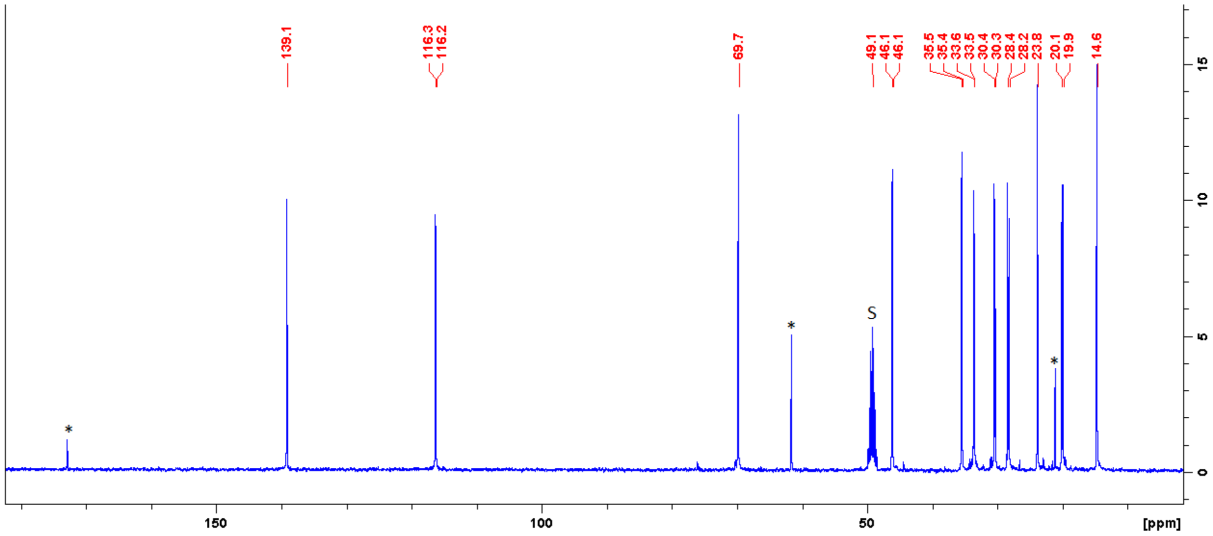

Peaks marked “S” are due to solvent CD_3OD and asterisked peaks are due to residual EtOAc.

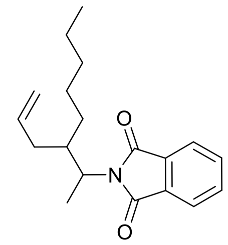
O
N
O

14

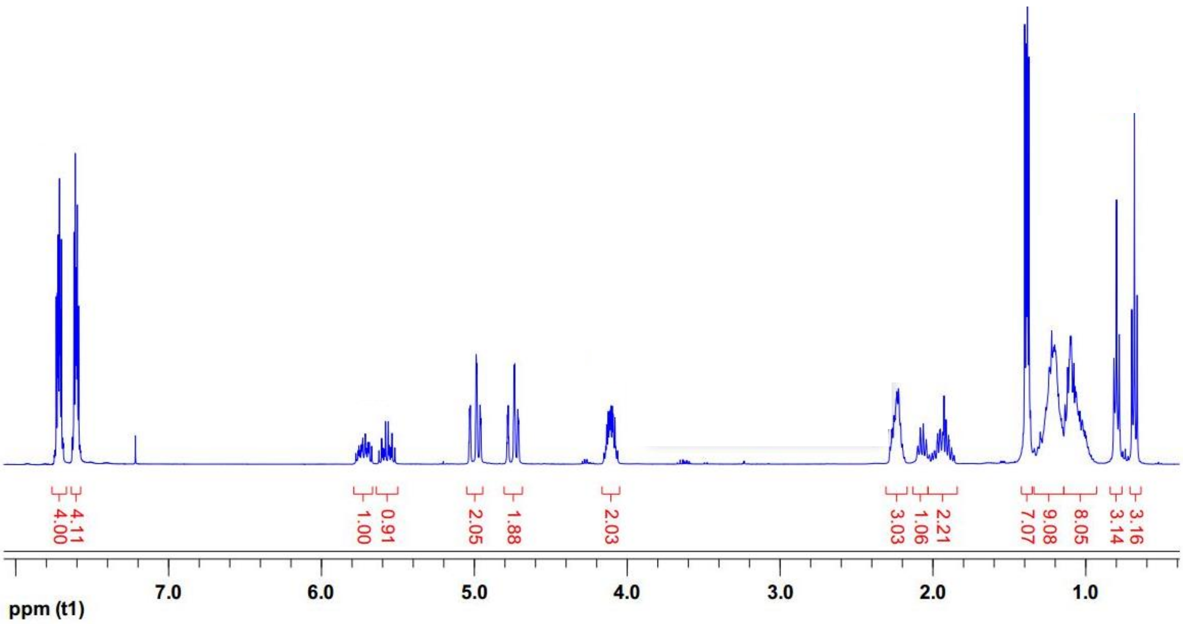
4.00
4.11
1.00
0.91
2.05
1.88
2.03
3.03
1.06
2.21
7.07
9.08
8.05
3.14
3.16
7.0
6.0
5.0
4.0
3.0
2.0
1.0
ppm (t1)

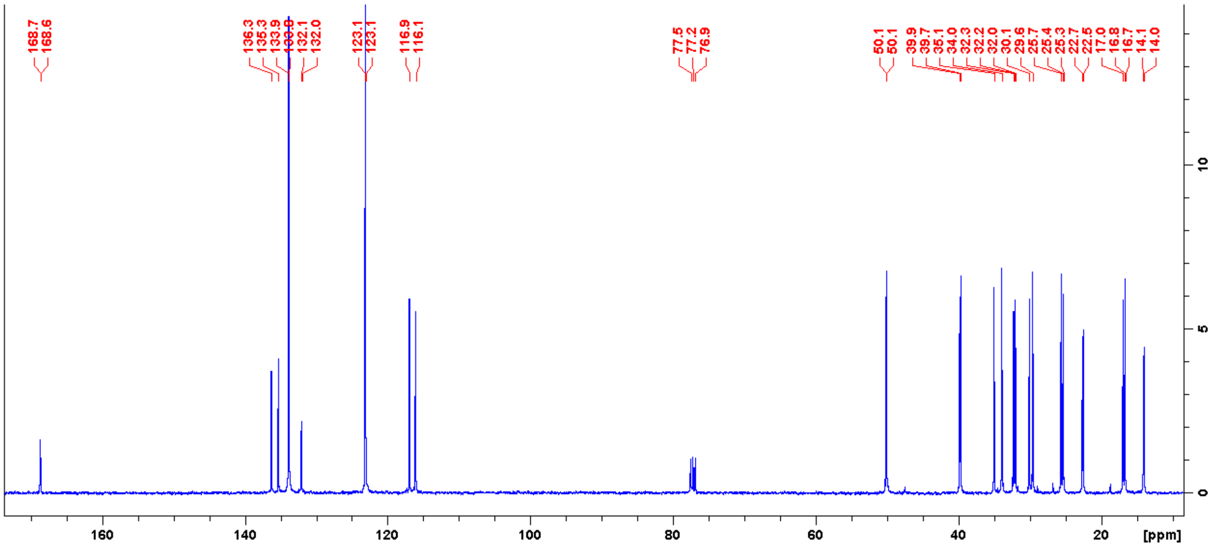
168.7
168.6
136.3
135.3
133.9
132.1
132.0
123.1
123.1
116.9
116.1
77.5
77.2
76.9
50.1
50.1
39.9
39.7
35.1
34.0
32.3
32.2
32.0
30.1
29.6
25.7
25.4
25.3
22.7
22.5
17.0
16.8
16.7
14.1
14.0
160
140
120
100
80
60
40
20
[ppm]
10
5
0

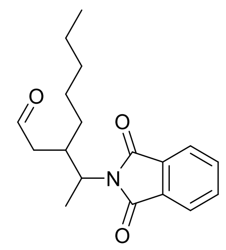

15

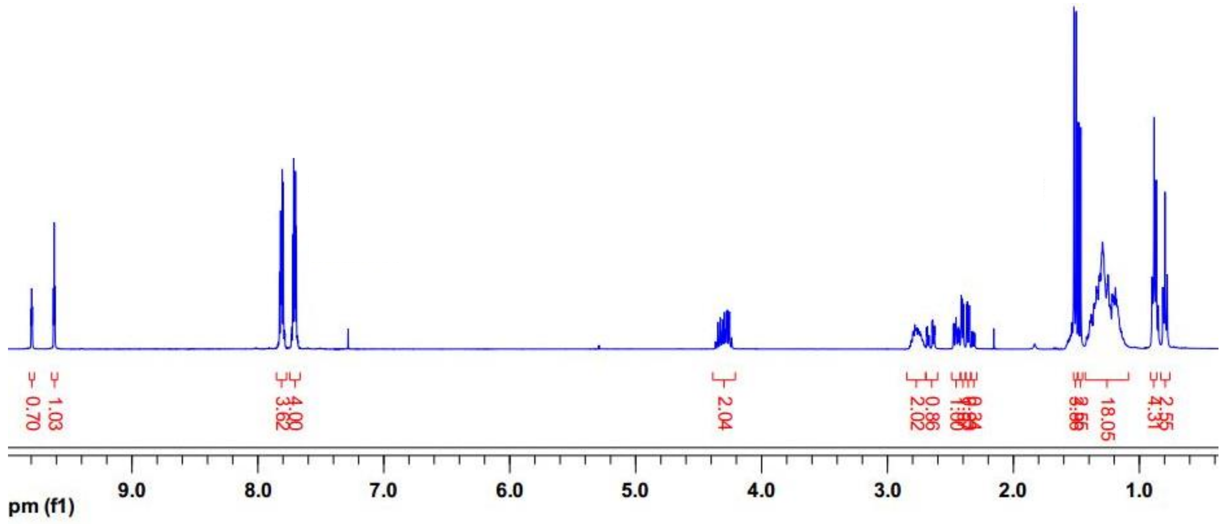

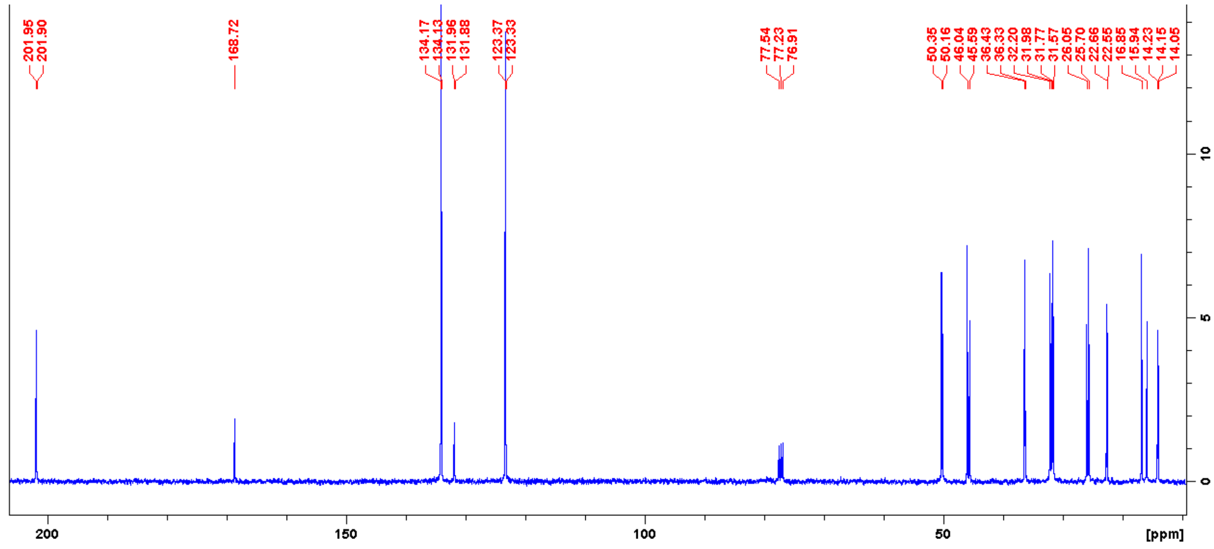

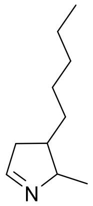

6

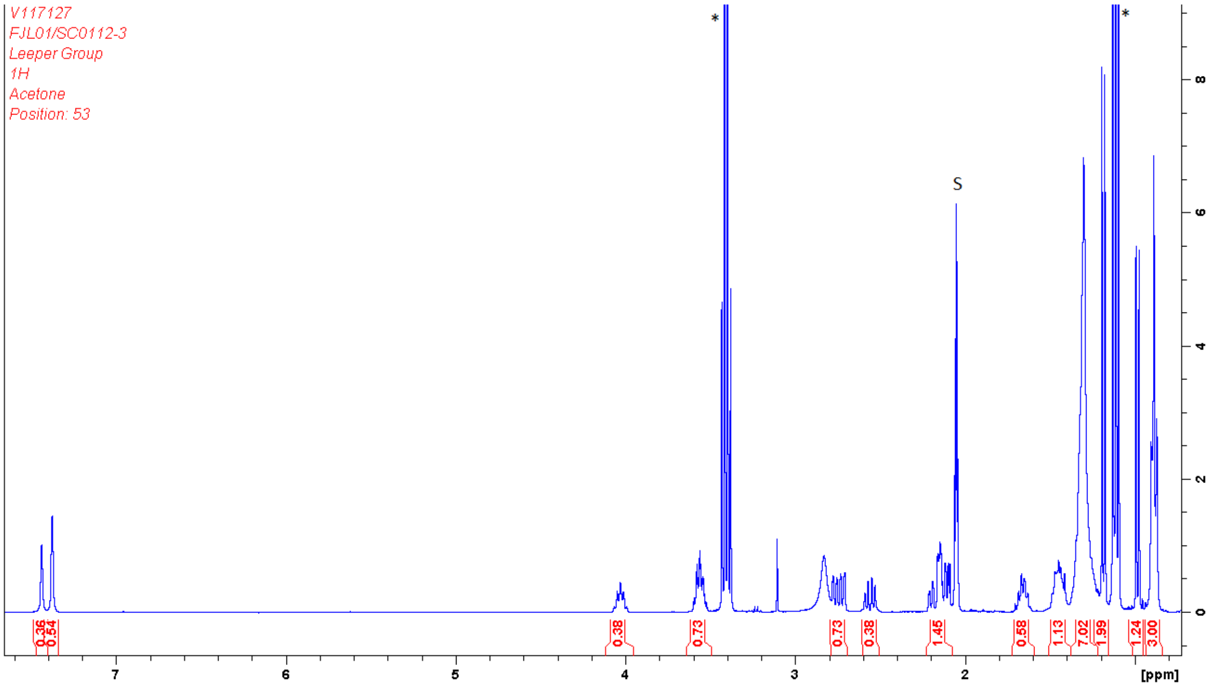

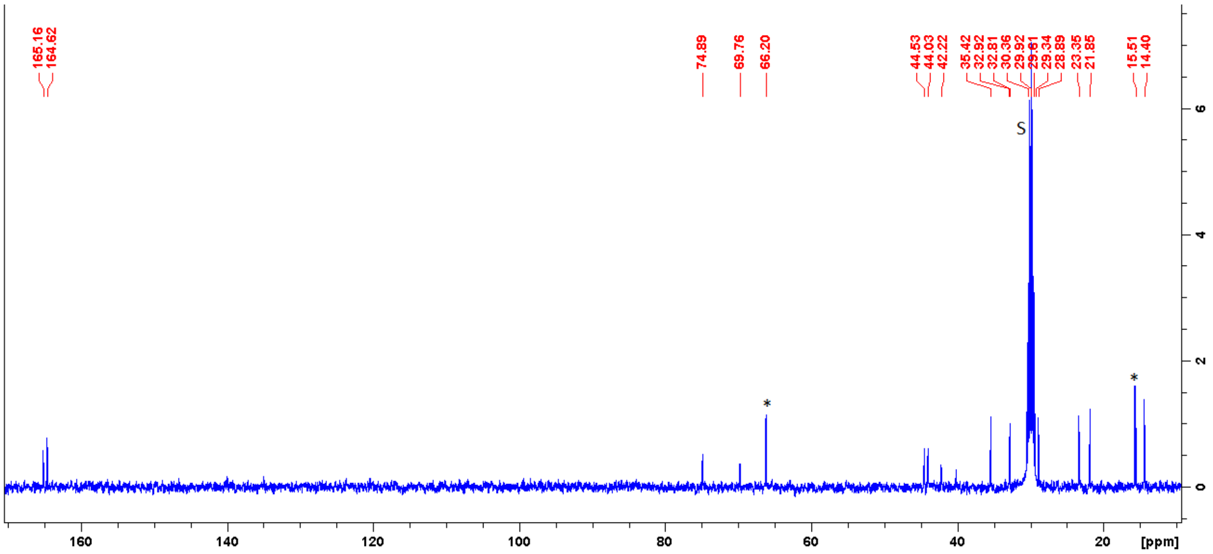

Peaks marked “S” are due to solvent CD_3COCD_3 and asterisked peaks are due to residual Et_2O.
